# Supplementary material for: Particle size distribution: An experimental study using southern African reduction methods and raw materials
Source: PLoS One. 2022 Dec 30;17(12):e0278867. doi: 10.1371/journal.pone.0278867 (PMC9803312; doi:10.1371/journal.pone.0278867)
Supplement: S2 File — (PDF) [file pone.0278867.s003.pdf]

Supplementary Material

Particle size distribution of Southern African rocks and methods of debitage

Paloma de la Pena, Marc Thomas and Tumelo Rufus Molefyane

Statistical information on the Principal Component Analyses implementations

Here we provide more data on the PCA implementations. The results of each PCA are detailed. To ensure the reproducibility of the statistical analyses, the codes needed to perform the principal component analyses are provided in an rmarkdown script accessible in the Supplementary Materials (SM3). Figure reproduction and more data are also available by removing from the figure's chunk code "fig.show = 'hide' " and by running more code chunks (under R).

PCA results description for the SA dataset with the raw material and knapping method as qualitative supplementary variable (same PCA but not same visualisation, figure 10, 11 and 12)

Table 1: Table containing eigenvalues, the percentage of variance and the cumulative percentage of variance

	eigenvalue	percentage of variance	cumulative percentage of variance
comp 1	2.9022907	41.461295	41.46130
comp 2	1.1690684	16.700977	58.16227
comp 3	1.0129851	14.471216	72.63349
comp 4	0.8889459	12.699228	85.33272
comp 5	0.6640289	9.486127	94.81884
comp 6	0.3626810	5.181157	100.00000
comp 7	0.0000000	0.000000	100.00000

Table 2: Table containing the variable coordinates

	Dim.1	Dim.2	Dim.3	Dim.4	Dim.5
p_2	-0.7948065	0.1476608	-0.5432012	0.2131353	-0.0673747
p_4	-0.4751180	0.4336603	0.4065816	-0.5900258	0.2662770
p_5	-0.4102553	-0.3225716	0.6506182	0.5236758	0.1579062
p_10	0.3172041	-0.8291324	-0.1604683	-0.3917895	0.1632008
p_20	0.7757334	0.1999930	0.2480087	-0.0550065	-0.5155892
p_31	0.8459206	0.0734205	0.0287826	0.1661976	0.1846658
p_sup_50	0.6771738	0.3497181	-0.2030222	0.1923698	0.4869122

Table 3: Table containing the variable contributions

	Dim.1	Dim.2	Dim.3	Dim.4	Dim.5
p_2	21.766167	1.865050	29.1285182	5.1101703	0.6836081
p_4	7.777894	16.086422	16.3189569	39.1621598	10.6777653
p_5	5.799191	8.900459	41.7877888	30.8496049	3.7550131
p_10	3.466862	58.804128	2.5420001	17.2675341	4.0110477
p_20	20.734045	3.421288	6.0719880	0.3403706	40.0332402
p_31	24.655756	0.461099	0.0817819	3.1072357	5.1355384
p_sup_50	15.800085	10.461555	4.0689662	4.1629247	35.7037871

PCA results description for the SA and EU dataset comparison with the raw material and knapping method as qualitative supplementary variable (same PCA but not same visualisation, figure 14 and 15)

Table 4: Table containing eigenvalues, the percentage of variance and the cumulative percentage of variance

	eigenvalue	percentage of variance	cumulative percentage of variance
comp 1	2.0988723	29.983890	29.98389
comp 2	1.9108087	27.297268	57.28116
comp 3	1.0100275	14.428965	71.71012
comp 4	0.7797606	11.139437	82.84956
comp 5	0.6366662	9.095232	91.94479
comp 6	0.4706393	6.723419	98.66821
comp 7	0.0932253	1.331790	100.00000

Table 5: Table containing the variable coordinates

	Dim.1	Dim.2	Dim.3	Dim.4	Dim.5
p_2	-0.9027179	-0.3585890	-0.0479784	0.0626491	0.0010896
p_4	0.5322509	-0.3551774	0.5570108	-0.4417261	-0.1874955
p_5	0.6667480	-0.2963243	0.1466766	0.4012535	0.5265489
p_10	0.7273024	0.1837233	-0.4519801	0.1438753	-0.4082048
p_20	0.0695452	0.7164915	-0.1741196	-0.5332817	0.3715117
p_31	-0.0454646	0.8251461	0.0553920	0.2538351	-0.0255140
p_sup_50	-0.1423252	0.5833380	0.6620262	0.2240259	-0.1376726

Table 6: Table containing the variable contributions

	Dim.1	Dim.2	Dim.3	Dim.4	Dim.5
p_2	38.8255912	6.729407	0.2279073	0.5033481	0.0001865
p_4	13.4972966	6.601969	30.7180741	25.0233150	5.5216605
p_5	21.1805586	4.595335	2.1300438	20.6479255	43.5477426
p_10	25.2025241	1.766491	20.2257866	2.6546730	26.1724448
p_20	0.2304350	26.866115	3.0016646	36.4713719	21.6786919
p_31	0.0984828	35.632348	0.3037808	8.2630811	0.1022458
p_sup_50	0.9651117	17.808335	43.3927429	6.4362854	2.9770279

PCA results description for the SA, EU and ESKI dataset comparison with the type of knapping as qualitative supplementary variable (figure 16 from the article)

Table 7: Table containing eigenvalues, the percentage of variance and the cumulative percentage of variance

	eigenvalue	percentage of variance	cumulative percentage of variance
comp 1	2.2505356	56.263389	56.26339
comp 2	1.0248592	25.621480	81.88487
comp 3	0.6176909	15.442274	97.32714
comp 4	0.1069143	2.672857	100.00000

Table 8: Table containing the variable coordinates

	Dim.1	Dim.2	Dim.3	Dim.4
p_2	-0.9543650	0.1664418	0.0297016	0.2461755
p_4	0.6372603	0.5613758	-0.5204802	0.0886385
p_5	0.6996433	0.4025617	0.5836536	0.0882712
p_sup_10	0.6664245	-0.7210808	-0.0725090	0.1751094

Table 9: Table containing the variable contributions

	Dim.1	Dim.2	Dim.3	Dim.4
p_2	40.47092	2.703091	0.1428197	56.683165
p_4	18.04463	30.749863	43.8568332	7.348678
p_5	21.75041	15.812509	55.1491851	7.287896
p_sup_10	19.73404	50.734537	0.8511620	28.680261

PCA results description for the SA and RUNOFF dataset comparison (figure 17 from the article)

Table 10: Table containing eigenvalues, the percentage of variance and the cumulative percentage of variance

	eigenvalue	percentage of variance	cumulative percentage of variance
comp 1	2.2904881	45.809762	45.80976
comp 2	1.5511838	31.023677	76.83344
comp 3	0.6135143	12.270286	89.10372
comp 4	0.4132875	8.265750	97.36948
comp 5	0.1315262	2.630525	100.00000

Table 11: Table containing the variable coordinates

	Dim.1	Dim.2	Dim.3	Dim.4	Dim.5
p_2	-0.9297931	-0.2095754	-0.1263886	0.0353974	0.2726461
p_4	0.0382188	0.8482070	0.5033241	0.1187902	0.1078792
p_5	0.4555949	0.6991018	-0.4883354	-0.2366367	0.0960292
p_10	0.8352289	-0.2223862	-0.1474239	0.4668503	0.1151257
p_sup_20	0.7206512	-0.4996078	0.2898271	-0.3521041	0.1519108

Table 12: Table containing the variable contributions

	Dim.1	Dim.2	Dim.3	Dim.4	Dim.5
p_2	37.7437119	2.831505	2.603700	0.3031728	56.517911
p_4	0.0637713	46.381033	41.292466	3.4143565	8.848373
p_5	9.0621172	31.507759	38.869752	13.5491428	7.011229
p_10	30.4567127	3.188251	3.542512	52.7354992	10.077025
p_sup_20	22.6736868	16.091452	13.691570	29.9978287	17.545463
