# Supplementary material for: Particle size distribution: An experimental study using southern African reduction methods and raw materials
Source: PLoS One. 2022 Dec 30;17(12):e0278867. doi: 10.1371/journal.pone.0278867 (PMC9803312; doi:10.1371/journal.pone.0278867)

PLOS ONE

Particle size distribution of Southern African rocks and methods of debitage

--Manuscript Draft--

**Manuscript Number:**	PONE-D-22-10277
**Article Type:**	Research Article
**Full Title:**	Particle size distribution of Southern African rocks and methods of debitage
**Short Title:**	Particle size distribution of Southern African rocks and methods of debitage
**Corresponding Author:**	Paloma de la Peña, Ph.D<br>Cambridge University & University of the Witwatersrand<br>Johannesburg, Gauteng SOUTH AFRICA
**Keywords:**	Lithics, site formation, Experimental Archaeology, particle size analysis
**Abstract:**	We performed a particle size analysis database focusing on reduction sequences and raw materials typical of the Middle and Later Stone Age record of Southern Africa. We reduced 117 cores of quartz, quartzite, jasper, chalcedony, hornfels, and rhyolite. The knapping methods selected were unidirectional, discoidal, Levallois recurrent, and bipolar flaking. In this article we compare this new particle size distribution dataset on Southern African raw materials and knapping methods with previous experiments. The Southern African dataset on knapping we have produced show a wider size distribution. Differences among knapping methods and raw materials seem to explain this variability.<br>Finally, we show an overlap between the distributions of the Southern African experimental knapping dataset and the sorting experiments carried out by A. Lenoble on flint artifacts in runoff contexts. This article shows that a size analysis of a lithic assemblage must be combined with a technological analysis to specify its implementation and to properly interpret its results. We also show that the size distribution analysis of lithic material is not sufficient on its own to assess the perturbation of an archaeological assemblage and must be coupled with other analytical tools.
**Order of Authors:**	Paloma de la Peña, Ph.D
	Marc Thomas
	Tumelo Rufus Molefyane
**Additional Information:**	
**Question**	**Response**
**Financial Disclosure**<br><br>Enter a financial disclosure statement that describes the sources of funding for the work included in this submission. Review the submission guidelines for detailed requirements. View published research articles from *PLOS ONE* for specific examples.<br><br>This statement is required for submission and **will appear in the published article** if the submission is accepted. Please make sure it is accurate.	PdlP received CoE-NRF (South Africa) funding

Powered by Editorial Manager® and ProduXion Manager® from Aries Systems Corporation

**Unfunded studies**<br>Enter: *The author(s) received no specific funding for this work.*<br><br>**Funded studies**<br>Enter a statement with the following details:<br>• Initials of the authors who received each award<br>• Grant numbers awarded to each author<br>• The full name of each funder<br>• URL of each funder website<br>• Did the sponsors or funders play any role in the study design, data collection and analysis, decision to publish, or preparation of the manuscript?<br>• **NO** - Include this sentence at the end of your statement: *The funders had no role in study design, data collection and analysis, decision to publish, or preparation of the manuscript.*<br>• **YES** - Specify the role(s) played.<br><br>* typeset	
**Competing Interests**<br><br>Use the instructions below to enter a competing interest statement for this submission. On behalf of all authors, disclose any competing interests that could be perceived to bias this work—acknowledging all financial support and any other relevant financial or non-financial competing interests.<br><br>This statement is required for submission and **will appear in the published article** if the submission is accepted. Please make sure it is accurate and that any funding sources listed in your Funding Information later in the submission form are also declared in your Financial Disclosure statement.<br><br>View published research articles from *PLOS ONE* for specific examples.	The authors have declared that no competing interests exist.

Powered by Editorial Manager® and ProduXion Manager® from Aries Systems Corporation

**NO authors have competing interests**<br><br>Enter: *The authors have declared that no competing interests exist.*<br><br>**Authors with competing interests**<br><br>Enter competing interest details beginning with this statement:<br><br>*I have read the journal's policy and the authors of this manuscript have the following competing interests: [insert competing interests here]*<br><br>* typeset	
**Ethics Statement**<br><br>Enter an ethics statement for this submission. This statement is required if the study involved:<br><br>• Human participants<br>• Human specimens or tissue<br>• Vertebrate animals or cephalopods<br>• Vertebrate embryos or tissues<br>• Field research<br><br>Write "N/A" if the submission does not require an ethics statement.<br><br>General guidance is provided below. Consult the submission guidelines for detailed instructions. **Make sure that all information entered here is included in the Methods section of the manuscript.**	N/A

**Format for specific study types**<br><br>**Human Subject Research (involving human participants and/or tissue)**<br>• Give the name of the institutional review board or ethics committee that approved the study<br>• Include the approval number and/or a statement indicating approval of this research<br>• Indicate the form of consent obtained (written/oral) or the reason that consent was not obtained (e.g. the data were analyzed anonymously)<br><br>**Animal Research (involving vertebrate animals, embryos or tissues)**<br>• Provide the name of the Institutional Animal Care and Use Committee (IACUC) or other relevant ethics board that reviewed the study protocol, and indicate whether they approved this research or granted a formal waiver of ethical approval<br>• Include an approval number if one was obtained<br>• If the study involved *non-human primates*, add *additional details* about animal welfare and steps taken to ameliorate suffering<br>• If anesthesia, euthanasia, or any kind of animal sacrifice is part of the study, include briefly which substances and/or methods were applied<br><br>**Field Research**<br><br>Include the following details if this study involves the collection of plant, animal, or other materials from a natural setting:<br>• Field permit number<br>• Name of the institution or relevant body that granted permission	
**Data Availability**<br><br>Authors are required to make all data underlying the findings described fully available, without restriction, and from the time of publication. PLOS allows rare exceptions to address legal and ethical concerns. See the PLOS Data Policy and FAQ for detailed information.	Yes - all data are fully available without restriction

Powered by Editorial Manager® and ProduXion Manager® from Aries Systems Corporation

A Data Availability Statement describing where the data can be found is required at submission. Your answers to this question constitute the Data Availability Statement and **will be published in the article**, if accepted.<br><br>**Important:** Stating 'data available on request from the author' is not sufficient. If your data are only available upon request, select 'No' for the first question and explain your exceptional situation in the text box.<br><br>Do the authors confirm that all data underlying the findings described in their manuscript are fully available without restriction?	
**Describe where the data may be found in full sentences. If you are copying our sample text, replace any instances of XXX with the appropriate details.**<br><br>• If the data are **held or will be held in a public repository**, include URLs, accession numbers or DOIs. If this information will only be available after acceptance, indicate this by ticking the box below. For example: *All XXX files are available from the XXX database (accession number(s) XXX, XXX.*).<br>• If the data are all contained **within the manuscript and/or Supporting Information files**, enter the following: *All relevant data are within the manuscript and its Supporting Information files.*<br>• If neither of these applies but you are able to provide **details of access elsewhere**, with or without limitations, please do so. For example:<br><br>*Data cannot be shared publicly because of [XXX]. Data are available from the XXX Institutional Data Access / Ethics Committee (contact via XXX) for researchers who meet the criteria for access to confidential data.*<br><br>*The data underlying the results presented in the study are available from (include the name of the third party*	The data will be available in a public repository after acceptance

Powered by Editorial Manager® and ProduXion Manager® from Aries Systems Corporation

and contact information or URL).<br>• This text is appropriate if the data are owned by a third party and authors do not have permission to share the data.<br><br>* typeset	
Additional data availability information:	Tick here if your circumstances are not covered by the questions above and you need the journal’s help to make your data available.

Powered by Editorial Manager® and ProduXion Manager® from Aries Systems Corporation

Click here to access/download;Manuscript;text_6_4_22_dlP_T_M.docx

Particle size distribution of Southern African rocks and methods of debitage

de la Peña, P.[1,2,3¶*], Thomas, M.[4¶]; Molefyane, T.R.[5]

1 Evolutionary Studies Institute, University of the Witwatersrand, South Africa
2 McDonald Institute for Archaeological Research, University of Cambridge, United Kingdom
3 Center of Exploration of the Deep Human Journey, University of the Witwatersrand, South Africa
4 UMR 5608 TRACES, Université Toulouse Jean Jaurès, Toulouse, France
5 Departamento de Arqueología. Guías de Espeleología y Montaña (GEM), Spain

*Corresponding author:

E-mail: paloma.delapenya@gmail.com (PdlP)

Abstract

We performed a particle size analysis database focusing on reduction sequences and raw materials typical of the Middle and Later Stone Age record of Southern Africa. We reduced 117 cores of quartz, quartzite, jasper, chalcedony, hornfels, and rhyolite. The knapping methods selected were unidirectional, discoidal, *Levallois* recurrent, and bipolar flaking. In this article we compare this new particle size distribution dataset on Southern African raw materials and knapping methods with previous experiments. The Southern African dataset on knapping we have produced show a wider size distribution. Differences among knapping methods and raw materials seem to explain this variability. Finally, we show an overlap between the distributions of the Southern African experimental knapping dataset and the sorting experiments carried out by A. Lenoble on flint artifacts in runoff contexts. This article shows that a size analysis of a lithic assemblage must be combined with a technological analysis to specify its implementation and to properly interpret its results. We also show that the size distribution analysis of lithic material is not sufficient on its own to assess the perturbation of an archaeological assemblage and must be coupled with other analytical tools.

Introduction

Stone Age sites are complex sedimentary accumulations formation of which depends on a combination of anthropogenic and natural processes [24, 39, 42, 49]. Natural processes, including sedimentary processes, have often altered the spatial organization, composition, and chronological signal of the archaeological record [6]. To measure this loss of information on sites, disturbance assessment studies need to be carried out. Any archaeological remains can undergo the effect of the sedimentary processes, governed by physical laws [7, 24]. Thus, particle shape, size and density are the main physical criterion to measure the degree of perturbation of the archaeological record [39].

The particle size analysis of lithic artefacts was first applied in Africa by K. Schick [39]. She pointed out that in many cases the sites are associated with sedimentary contexts affected by water bodies (alluvial environments, lake margins, basins, marine beaches or even spring deposits). In her seminal work, K. Schick [39] conducted experiments to understand the spatial dispersion and size-class distribution (maximal length of artefacts considered) of lithic industries affected by water flow. She concluded that water flow can substantially, if not radically, affect archaeological assemblages [35, 36]. In addition, other natural processes are likely to have an impact on the particle size distribution of lithic assemblages, such as solifluction [3, 25] trampling [9, 16, 19, 20, 21, 27, 55, 57, among others] or eolian processes [43].

Other studies have extended and followed a similar methodology to Schick's work, applying it, mainly, to European Palaeolithic contexts, particularly on the Southwest of France [2, 5, 24]. To allow systematic approaches on sites, A. Lenoble and P. Bertran set up a method based on sieving instead of manually measuring all lithic artifacts [2, 5, 24, among others]. Based on the observation that rock knapping consistently causes the same particle size distribution, A. Lenoble and P. Bertran and colleagues [2, 5, 24] proposed to compare experimental knapping assemblages (from [5] called "EU database" for ease of reading in this article) to experimental ones subject to overland flow (from [24] called "RUNOFF dataset" for ease of reading in this article). The particle size distribution of these two sets of experiments: "EU dataset" and the "RUNOFF dataset" show separated distributions in a ternary plot, (Fig 1). The knapping experiments were done mainly on flint and quartzite and included blade production, *Levallois* and Discoid flaking and bifacial shaping. The authors also added microlithic reduction sequences inspired of Paleoeskimo knapping methods from Eastern Canadian Artic (called "ESKI dataset" for ease of reading in this article, from [52].

In all knapping experiments, the distribution sizes follow a Weibull function being long tailed and positively skewed [44] with a proportion of largest pieces tending towards 0 [2, 5, 24, 39, 44, 52].

Fig 1: Particle size evolution of a lithic assemblage affected by overland flow from the experiment of A. Lenoble [24]. The hatched area is the distribution of the EU dataset [2, 5]. The dash areas correspond to the RUNOFF experiments size class

distribution [24]. The “A” triangle summit corresponds to the [2;4[mm width size class percentage, the “B” triangle summit to the [4;10[mm width size class percentage and “C” to the [10;+∞[mm width size class percentage. From [5].

Questions and objective

In this paper, we offer experimental research focusing, strictly, on Southern Africa knapping methods and raw materials. For this purpose, we have performed a large experiment on particle size analysis focusing on Southern African rock varieties (automorphic and xenomorphic quartz, quartzite, jasper, chalcedony, hornfels and rhyolite) and knapping methods typical of the Middle and Later Stone Age records (unidirectional, bipolar knapping, discoid, recurrent *Levallois*). The reason for developing this specific experiment relies on the fact that most of the recent analyses have focused on European reduction sequences (such as laminar, Discoid and *Levallois*) and raw materials, mainly flint [2, 5, 24]. This new experimental dataset (called “SA dataset” in this article) will be useful for our own research on Middle and Later Stone Age sites and offer a shareable dataset for other archaeologists working in Africa. Additionally, this new investigation serves as a control experiment that is comparable to previous European publications on this type of research [2, 5, 24, 52].

The research questions are:

(1) What is the particle size distribution of Southern African raw materials and knapping methods?

(2) Are there statistically significant differences regarding raw materials?

(3) Are there statistically significant differences regarding the knapping methods?

(4) Is the particle size distribution of the SA dataset different to the EU dataset?

(5) Is the particle size distribution of the SA dataset different to the RUNOFF dataset?

The null hypotheses are that there is no statistical difference between, on one hand, the different raw material and knapping method datasets compared in this study and, on the other hand, with previous published datasets [2, 5, 24].

Material and methods

One of the authors (PdlP) did all the experiments, having several years of stone tool making competence. The experiment includes the most common Southern Africa rocks and lithic reductions used during the Middle and the Later Stone Age. They are summarized in Table 1 with the number of cores reduced for each one of the knapping methods and raw material. In Fig 2, we show schematically all the knapping methods deployed in this analysis. In total 117 cores were knapped for this experiment. In Fig 3 the main raw materials are illustrated, alongside a selection of tools made during the experiments.

Table 1: Synthesis of the knapping methods and raw materials used in this experiment (A-quartz = Automorphic quartz, X-quartz = Xenomorphic quartz)

	Chalcedony	A-quartz	Hornfels	Jasper	Quartzite	Rhyolite	X-quartz	Total
**Bipolar**	8	4		10			10	**32**
**Discoidal**			10		10	10	12	**42**
**Levallois**	4		16					**20**
**Unidirectional**	10		8	5				**23**
**Total**	**22**	**4**	**34**	**15**	**10**	**10**	**22**	**117**

Fig 2: Examples of Middle and Later Stone Age reductions sequences done in this knapping experiment: discoidal, Levallois recurrent, unidirectional, and axial bipolar knapping. The figures are modified from [15, 40, 48, 53]. On the left freehand variants and on the right bipolar axial variant towards miniaturization. Photos: Paloma de la Peña

Fig 3: Raw materials used for this experiment. A. Hornfels (Unidirectional core and flake). B. Jasper (Unidirectional core and flake). C. Chalcedony (chunk). D. Rhyolite (core fragment) and flake (ventral view). E. Quartzite (Discoidal core and flake, dorsal view). F. Xenomorphic quartz (Bipolar core). G. Xenomorphic quartz (core fragment). H. Automorphic quartz (Bipolar core). I. Selection of blanks and cores in hornfels during one of the Unidirectional reduction sequences. J. Core and blanks in the middle of the reduction through Bipolar axial knapping of one of the automorphic cores. All scales are 1 cm except for H, which is 5 mm. All the material presented is from the experiments except C and H rocks. Photos: Paloma de la Peña.

During the experiments, we tried to reduce the core until exhaustion, in other words, until no more flakes could be extracted. For the knapping technique in this experiment, we used a hard mineral percussion with a banded iron pebble from Kwa-Zulu Natal and a soft mineral hammer, a sandstone pebble from the Eastern Cape.

The specific knapping methods selected are:

- Bipolar knapping (i.e., knapping on an anvil) is a method in which the core is placed on an anvil and held with the bare hand. The rock is hit from above with a hammer held in the other hand, causing blanks to fly off from the top and from the edge that is in direct contact with the anvil [13, 14]. Bipolar knapping has been reported in the Middle Stone Age and Later Stone Age [5].
- Discoidal Knapping: In this experiment we used what Terradas named multifacial discoidal knapping [48] (called "Discoidal knapping" but equivalent to "Centripetal knapping" according to V. Mourre [30]. In this sub-variant the knapper searches for 45 degrees angles to obtain flakes until the core is exhausted, changing the striking platform almost every time and using the previous removals as such.
- Unidirectional knapping: In this method, we tried to exploit, as much as possible, from one striking platform and following a unidirectional sequence of removals. Once the knapping surface from the first striking platform is exhausted, we rotated the core and continued knapping, from a different striking platform, following the same strategy if possible.
- *Levallois* recurrent (also referred in the African literature as 'prepared cores'): In our experiments we followed the guidelines explained by E. Boëda [8]. Sometimes during reduction, we could extract two series of *Levallois* flakes. In some cases, we finished the knapping of these cores using discoidal reduction. This was done to obtain the most out of the volume of the rock in the final stage, and only when there was no possibility to prepare two hierarchical knapping surfaces in a *Levallois* fashion.

The raw materials used for the experiments (Fig 3) are:

- Hornfels is a fine- to medium- grained metamorphic rock that is formed by contact metamorphism on the margins of igneous intrusions that crosscut the country rock [12]. The two varieties of hornfels used for this study come from two different dolerite dykes that intruded on shales, one in the Eastern Cape and another one in the Northern Cape. The variety used from the Eastern Cape was obtained from a river cutting of a massive hornfels contact metamorphism layer. Big chunks of the material were obtained and broken into smaller blocks for the experiments. The hornfels of Northern Cape came in small slabs, usually of less than 10 cm for their longest axis.
- Chalcedony is a cryptocrystalline quartz [12]. The one we use is not banded, and in South Africa it mostly occurs in white, gray, black, or blue shades. Chalcedonies fill cavities and line fissures in rocks. In the Drakensberg and Lebombo Group, they occur in both basalts and rhyolites. Sometimes chalcedony contains a monoclinic polymorph called moganite [12]. The chalcedony of this experiment comes entirely from Siberia Farm, near a Rock Art site also known in the area as Franshoek. It is an outcrop with varieties of rocks ranging in colour from green to orange.
- Jasper is a chert colored by the presence of iron [12]. The variety used for this experiment was banded red jasper from the Eastern Cape.
- Automorphic quartz (Crystal quartz, called A-quartz in this article, following [29] is a euhedral mineral with the shape of a big hexagonal single crystal terminated alternately by major and minor rhombohedral faces [47]. Its flat faces are well developed around a symmetrical axis and bounded by rectilinear ridges [37, 38]. The crystal quartz used for this experiment come from a small vein in Limpopo, from the surroundings of Mwulu's Cave [17], specifically from the Black Reef formation.
- Xenomorphic quartz (following [29], called X-quartz in this article) is a solid polycrystalline aggregate with an anhedral texture formed by a close-packed mass of crystals that are not usually bounded by their own crystal faces but have their outlines impressed on them by the adjacent crystals [1, 38]. All the xenomorphic quartz used for this experiment come from the surroundings of the Cradle of Humankind (Gauteng), near the famous Swartkrans Cave.

- Quartzite is a metamorphosed sandstone. All the Quartzite used in this experiment comes from the Black Reef formation in the surroundings of Mwulu's Cave [17].
- Rhyolite is an extrusive igneous rock with a high silica content. Rhyolite is made up of quartz, plagioclase, and sanidine, with minor amounts of hornblende and biotite. Trapped gases often produce vugs in the rock. These often contain crystals, opal, or glassy material. The rhyolite used for this experiment comes from the immediate surrounding of Border Cave in the Lebombo mountains (Kwa-Zulu Natal, South Africa).

To allow comparison with this Southern African experimental knapping dataset with the RUNOFF dataset [24] and the EU dataset [2, 5] we keep the same size class. They used meshes of 2, 4, 5, 10, 20, 31.5 and 50 mm. We hand sieved the experiments the same way recommended in Bertran et al [2, 5] after gathering all the particles produced during the knapping process.

To describe our dataset and to compare it with previous data we used ternary plots made with Past3. Additional statistics were made thanks to the method developed by Weaver et al [56] to obtain 95% confidence intervals in ternary plots (Fig 4). To compare the different datasets, we use Principal Component Analysis (PCA) performed under R using the FactoMineR package [23].

Raw material and reduction strategies choices has been realized to stick as much as possible to the Southern African archaeological context. Thus, all knapping methods are not applied to all raw materials. We assume statistical bias in the comparisons we performed as each raw material have not been knapped through each knapping method. Then it is not possible to measure the exact effect of these two last variables on particle size distribution. The aim being to compare archaeological compatible options.

Fig 4: Illustration of how we applied the Weaver et al method to create 95% confidence ellipses intervals in the ternary plots.

Results

What is the particle size distribution of Southern African raw materials and knapping methods?

In the supplementary material 1 (S1) we present the particle size composition of the SA dataset, describing the size class and their weight counterpart. As particle size analysis is time consuming, to speed up the counting for material rich assemblages, weights of small particles (2 and 4 mm) are useful to obtain mass/counts ratios. We counted all the particle per size class and weighed them. In this article we use only counts in the statistics and in the figures.

The histogram of the percentages per size classes considering knapping methods and raw materials clearly show that for all the raw materials and knapping methods there is a gradual decrease of particle numbers, as size classes increase. In all the series, the 5 mm particle class is slightly more abundant than the 4 mm size class (Fig 5).

In all the experiments, either considering raw material or knapping method, the size classes 2 to 4 mm constitutes around 40-60% of the particles (Fig 6).

Fig 5: Histograms comparing mean distributions of percentage of particles per size class (counts).

Fig 6: Box plots comparing the proportion of the [2:4[mm size class (counts) depending on the raw material and the knapping method.

The ternary diagrams (Fig 7) show a broad distribution of the SA dataset on experimental debitage size class distribution, which are predominantly composed of particles between 2 and 4 mm and between 4 and 10 mm. The 95% confidence interval calculated for each debitage considerably increases the range of distribution for each raw material and knapping method.

In the ternary plots (Fig 8) rhyolite, quartzite and hornfels have a similar distribution towards the center of the charts. Regarding knapping methods, unidirectional, discoidal, and *Levallois* seem to overlap

partially, independently of the raw material considered (Fig 9). Furthermore, bipolar seems to produce the smallest fractions (between 2 and 4 mm) independently of the raw material.

Freehand knapping (unidirectional, discoidal and *Levallois*) and bipolar knapping distributes slightly separately in the ternary plot.

Fig 7: Ternary plots per raw material and knapping methods of the SA dataset (counts). Shaded areas are 95% confidence intervals.

Fig 8: Ternary plots of the particle size distribution of SA dataset per raw material (counts). Shaded areas are 95% confidence intervals.

Fig 9: Ternary plots of the particle size distribution of the SA dataset per knapping method (counts). Shaded areas are 95% confidence intervals.

Are there statistically significant differences regarding the raw material compare?

The factorial space of the "Raw material" principal component analysis (Fig 10) shows a clear opposition (mostly first axis, 39%) between, on one hand, chalcedony, jasper, X-quartz and A-quartz and, on the other hand, hornfels, quartzite and rhyolite. The latter producing larger pieces but have not been reduced through bipolar technique. These differences are confirmed by a MANOVA test using a pairwise comparison) with the raw material as an independent variable and the size distributions as dependent variable Table 2. A group composed of chalcedony, jasper and x-quartz differs to hornfels, quartzite and rhyolite.

Table 2. Results of the pairwise comparison from the MANOVA test. Raw material is the independent variable, Size class are the dependant variables.

	**Chalcedony**	**A-Quartz**	**Jasper**	**X-quartz**	**Hornfels**	**Quartzite**	**Rhyolite**
**Chalcedony**		18,09	0,16	1,51	***0,00***	***0,00***	***0,02***
**A-Quartz**	18,09		11,61	12,17	0,14	1,22	3,50
**Jasper**	0,16	11,61		9,63	***0,00***	***0,00***	***0,01***
**X-quartz**	1,51	12,17	9,63		***0,00***	***0,00***	***0,00***
**Hornfels**	***0,00***	0,14	***0,00***	***0,00***		***0,01***	13,83
**Quartzite**	***0,00***	1,22	***0,00***	***0,00***	***0,01***		9,40

**Rhyolite**	***0,02***	3,50	***0,01***	***0,00***	13,83	9,40	

Are there statistically significant differences regarding the knapping methods?

The factorial space of the “Knapping method” principal component analysis (Fig 11) shows a clear opposition on the first dimension (39% of the variability) between industries producing smaller particles (2 to 4 and 4 to 5 mm width size class) and industries producing larger particles (over 10 mm wide). Bipolar knapping is best described by the variables 2 to 4 mm and 4 to 5 mm and are opposed to *Levallois*, unidirectional and discoidal the distribution of which seems similar and produces larger remains. These differences are confirmed by a MANOVA test with “Kanpping method” as an independent variable (Table 3). Bipolar differs to all other knapping methods which size distribution tends to be the same.

Table 3. Results of the pairwise comparison from the MANOVA test. Knapping method is the independent variable, Size class are the dependant variables.

	bipolar	discoidal	Levallois	unidirectional
**bipolar**		***0,00***	***0,00***	***0,00***
**discoidal**	***0,00***		2,48	0,11
**Levallois**	***0,00***	2,48		2,41
**unidirectional**	***0,00***	0,11	2,41	

When combining knapping methods and raw material (Fig 12), three groups appear on the factorial space. The first one is best described by small particles (2 to 4 mm), and is composed by X-quartz Discoidal knapping, all Bipolar on anvil and all jasper knapping experiments. The second group is best described by larger particles. It is composed of all hornfels, quartzite and rhyolite raw material regardless of the associated knapping method. A third group composed of chalcedony associated to *Levallois* and unidirectional flaking is described by intermediate width particles (5 to 10 mm size class).

Fig 10: PCA of the SA dataset with the raw material as a supplementary variable.

Fig 11: PCA of the SA dataset with the knapping method as a supplementary variable.

Fig 12: PCA combining knapping and raw material as a supplementary variable (SA dataset).

Is the particle size distribution of the SA dataset different to the EU dataset?

On the ternary diagram we see great differences between the three experimental knapping datasets on particle size distributions (Fig 13).

This variability is confirmed by the principal component analysis including the EU dataset on particle size distribution from the European archaeological context [2, 5] and the SA dataset (Figs 14 and 15). When considering the knapping methods (Fig 14), three main groups appear on the factorial space. The first one is correlated to small particles (i.e., 2 to 4 mm) and is composed of Bifacial shaping and Laminar knapping systems. A second group best described by intermediate particle sizes is composed of Bipolar blank production. The third group is best described by larger particle sizes and is composed of the flaking systems (Discoidal, *Levallois* and Unidirectional flaking).

When considering raw materials as a supplementary variable (Fig 15), flint is opposed on the factorial space to A-quartz, X-quartz, jasper, and chalcedony which are all opposed to a group composed of rhyolite, quartzite and hornfels. These three clusters are best described respectively by small particle sizes (2 to 4 mm), intermediate sizes (4 to 10 mm) and larger sizes (20 to 50 mm).

When adding the ESKI dataset in the principal component analysis with the data divided according to type of reduction, such as “knapping”, “shaping” and “retouching” (Fig 16), we see a clear opposition on the factorial space between “retouching”, producing smaller remains, and “knapping” producing larger remains. “Shaping” falls between these two.

Fig 13: Ternary plots comparing the SA dataset presented in this paper, the EU and the ESKI datasets published in Bertran et al. [2, 5] and the RUNOFF dataset [24]. The ternary plots contain 95% confidence intervals.

Fig 14: PCA of the SA dataset compared to the EU dataset [2, 5]. The PCA has been made considering the knapping method as a variable.

Fig 15: PCA of the SA dataset and the EU dataset [2, 5], having 'raw material' as a supplementary variable.

Fig 16: PCA comparing debitage, shaping and retouch from the SA dataset presented in this paper and the EU dataset and the ESKI dataset [2, 5].

Is the particle size distribution of the SA dataset different to the RUNOFF dataset?

The factorial space of the SA dataset compared to the RUNOFF model [24] (Fig 17) shows a clear opposition on the first dimension (53% of the variability) between, on one hand, the fine accumulation area composed of smaller remains (2 to 4 mm size class) and, on the other hand, industries (SA dataset) and runoff areas as residual and transit (in and off channel) described by particles over 4 mm wide. The SA experiments overlap with most of the runoff accumulations of large particles.

Fig 17: PCA comparing the SA dataset to the RUNOFF dataset [24].

Discussion

Variability within the SA dataset

Patterson [33: p. 550] highlighted the need of 'simple analytical technics' for analysing lithic assemblages that can be used for any archaeologist (and not only lithic specialist). This seems still today a valid point as lithic assemblages have the advantage of preservation, and they suppose the most evident continuous cultural record still available from the past [40, 41, 46].

The results of our experiment highlight two main points within the SA dataset: a distinction between raw materials and the difference between freehand and bipolar sets.

Concerning the raw materials, we see, on the one hand, that in our experimental sample chalcedony, X-quartz, A-quartz and jasper groups more or less together and produce smaller sizes; on the other hand, we found rhyolite, quartzite and hornfels producing wider sizes (Figs 8 and 10). Other authors like K. Schick [39] have also highlighted differences regarding raw materials. Our results prove that experiments with specific raw materials of the areas of study of archaeological assemblages are needed to understand particle size analysis.

Regarding the distinction between freehand and bipolar sets, from a particle size analysis point of view, it seems possible to distinguish these two big variants of reduction. This is in accordance with previous characterizations to distinguish quantitatively freehand from bipolar axial knapping [19, 31, 32].

Regarding the different freehand knapping methods in our sample, in the factorial space, it does not seem possible to differentiate unidirectional, discoidal and *Levallois* flaking systems.

However, as we have not knapped each raw material through all knapping methods, it remains difficult to know what variable (either the knapping method or the raw material) causes these differences. Our results show that the size distribution in Southern African archaeological assemblages is knapping method specific and raw material specific.

Due to the variability expressed by the SA dataset, the selection of a single raw material and knapping method appropriate to the site context provides a more accurate model for performing a particle size analysis. Indeed, when combining a specific raw material and a specific knapping method, the distribution areas tend to reduce (with and without 95% confidence intervals) in both, ternary plots, and principal component analysis.

Differences between SA, EU and ESKI datasets

Southern African lithic industries produce a greater proportion of small particles whatever the raw material and the knapping method (Fig 13). In the Southern African dataset, the smallest particles (2 to 4 mm) always constitute 50 to 60% of the reduction sequences. In the EU dataset [2, 5] (see Fig 13 therein) the percentage of this fraction was slightly higher, between 60-70% in all their variants.

It seems that our knapping and raw material particle size distribution differs from the ones published in Bertran and colleagues [2, 5]. In the case of the EU dataset [2, 5], the different experiments overlap remarkably in the multivariate analysis and in the ternary plots (Fig 11). The EU, ESKI and SA datasets are clearly separated from each other.

Some technical behaviors produce more small particles. This is particularly the case of microlithic production and retouching activities (ESKI dataset) and to a lesser degree, for bipolar knapping on anvil (SA dataset) and shaping (EU and ESKI datasets). Similar results have been pointed out in other experimental studies [2, 5, 33, 39].
The differences between Bertran and colleagues [2, 5] and our sample regarding freehand variants could be a result of the level of the skill of the knappers, due to the specific knapper gestures and procedures or due to raw material variability. As the datasets are dependent on different knappers, we cannot exclude the hypothesis that knapper ability and experience have an impact on particle size distribution. These different levels of knapping performance enrich the models, as it is expected than in the past there would also have been variability in knapping skills within cultural traditions and periods. Moreover, despite similar knapping goals, knapper-specific gestures, habits, and procedures (such a platform preparation or removing striking platform overhang) could also impact size distribution.

Raw material specificities also induce different patterns in terms of particle size distribution. Whereas flint knapping experiments have a narrow distribution, other types of raw material induce specific distributions with various dispersions features. Regardless of the knapping method, flint tends to produce finer particles than A-quartz, X-quartz, jasper, and chalcedony, which in turn produce finer particles than quartzite, rhyolite and hornfels. Finer raw materials (i.e., flint, jasper, and chalcedony),

A-quartz and X-quartz produce finer grain sizes in contrast to coarser metamorphic and volcanic raw materials like quartzite, hornfels and rhyolite.

Moreover, considering previous knapping datasets [2, 5] and our knapping experiments, it seems clear that blank transformation (“retouching”) produces smaller particles than “shaping” and blank production (“débitage”). The intensity of retouch and site function [34] also influence the particle size distribution of industries as it is already shown in Bertran et al. [2, 5, 52]. When a great proportion of the blank is transformed on a site, we could expect a greater proportion of the small fraction (2 to 4 mm).

SA knapping dataset versus RUNOFF experiments

Finally, as already exposed, the comparison of the SA dataset distribution and the RUONFF model [24] (for flint) shows a remarkable overlapping.

As discussed before, while taken as a whole, the SA dataset shows a broad distribution (contra EU dataset). At a site scale, it is nevertheless possible to reduce that distribution by considering only a single knapping method on a single raw material. This will reduce the overlapping with the RUNOFF model and allow a more accurate comparison to assess the archaeological record perturbation degree. This means to obtain the most accurate particle size model (i.e., a single knapping method used on a single material in a ternary diagram or in a PCA), we must perform the particle size analysis on a raw material that has been reduced by a single knapping method according to the technological analysis. Obviously, the distribution size analysis of lithic remains can only be applied to a site if there has been an act of debitage within it.

Also, when a lithic assemblage is sorted because of a water flow, an extreme caution needs to be taken when considering faunal remains that are more mobile in these contexts [51].

This article also stresses the need to combine several tools to assess site perturbation as the particle size distribution characterization is not sufficient to determine assemblage perturbation. Along a sedimentary

analysis [49], fabric analysis [22, 4, 28] refitting [10, 11, 45, 54, 55], spatial analysis [18, 26] material proportions [51] or dating [6] are all tools that can help characterize the impact of natural processes on archaeological assemblages. When all of them are combined [18, 51], we can reach a very detailed site formation model and its associated assemblage perturbation degree.

Conclusion

While all knapping experiments show the same general pattern of size distribution (following a Weibull function, i.e., proportion decreasing as size increases, [44]), we have demonstrated that the knapping method, blank transformation activity intensity, and raw material explain most of the variability.

The wide distribution of the SA dataset leads to a significant overlap with the RUNOFF database. To reduce this overlap, the experimental debitage model compared to the RUNOFF dataset should be knapping method and raw material specific. This means that the performance of a particle size analysis at a site must be done in combination with a technological study of the lithic material. The latter guides the choice of the knapping method and raw material to be included in the comparison model between experimental knapping and runoff experiences on sorting [24].
Both analyses are clearly dependent on one another (i.e., particle size distribution and technology). The first conditions the interpretation of the second and the second makes the first more accurate [50, 51].

Acknowledgments

We thank Gary Trower and Ben Maclennan for indicating us raw material outcrops and providing us some of the raw material we have used in our knapping experiments. This work has been supported by the Center of Excellence of Paleosciences - National Research Foundation of South Africa. Our thanks

also go to Tammy Hodgskiss, Matt Caruana and Matt Lotter whose reading and correction of the article was very valuable to us.

References

1. Allaby M. Oxford Dictionary of Earth Sciences, third ed. Oxford University Press. 2008.

2. Bertran P, Claud É, Detrain L, Lenoble A, Masson B, Vallin L. Composition granulométrique des assemblages lithiques, application à l'étude taphonomique des sites paléolithiques. Paléo. 2006; 18. pp. 7-36.

3. Bertran P, Klaric L, Lenoble A, Masson B, Vallin L. The impact of periglacial processes on Palaeolithic sites: The case of sorted patterned grounds. Quaternary International. 2010; Volume 214, Issues 1–2. pp. 17-29.

4. Bertran P, Lenoble A. Fabriques des niveaux archéologiques: méthode et premier bilan des apports à l'étude taphonomique des sites paléolithiques. Paléo. 2002; 14. 19 p.

5. Bertran P, Lenoble A, Todisco D, Desrosiers P, Sorensen M. Practicle size distribution of lithic assemblages and taphonomy of Palaeolithics sites. Journal of Archaeological Science. 2012; 39(10). pp. 3148-3166.

6. Bertran P, Todisco D, Bordes J-G, Discamps E, Vallin L. Perturbation assessment in archaeological sites as part of the taphonomic study: a review of methods used to document the impact of natural processes on site formation and archaeological interpretations. Paléo. 2019; 30-1. pp. 52-75.

7. Bishop WW. Paleogeomorphology and continental taphonomy, In: Behrensmeyer A.K. and Hill A.P. (éd.), Fossils in the making – Vertebrate taphonomy and paleoecology. The University of Chicago Press, Prehistoric Archeology and Ecology series. 1980. pp. 20-40.

8. Boeda E. Le concept Levallois: variabilité des méthodes. CNRS éd. 1994. 280 p.

9. Bordes F, Bourgon M. "Le Complexe Moustérien: Moustériens, Levalloisien et Tayacien." l'Anthropologie. 1951; 55(1–2). pp. 1–23.

10. Bordes J-G. Lithic taphonomy of the Châtelperronian/Aurignacian interstratifications in Roc de Combe and Le Piage (Lot, France). The chronology of the Aurignacian and of the transitional technocomplexes. Dating, stratigraphies, cultural implications. 2003. pp. 223-244.

11. Bordes J-G. À propos de taphonomie lithique: fondements épistémologiques et outils analytiques. Réflexions à partir du thème de la transition du Paléolithique moyen au Paléolithique supérieur. Habilitation à Diriger des Recherches. Université de Bordeaux I. 2012. 114 p.

12. Cairncross B. Field guide to rocks & minerals of Southern Africa. Penguin Random House South Africa. 2011.

13. Crabtree D. E. An introduction to flint working. Pocatello: Occasional Papers of the Idaho State University Museum. 1972.

14. de la Peña Alonso P. Sobre la identificación macroscópica de las piezas astilladas: propuesta experimental. Trabajos de Prehistoria. 2011; 68(1). pp.79-98.

15. de la Peña P. The interpretation of bipolar knapping in African Stone Age studies. Current Anthropology. 2015; 56(6). pp. 911-23.

16. de la Peña P, Witelson D. Trampling vs. Retouch in a Lithic Assemblage: A Case Study from a Middle Stone Age Site, Steenbokfontein 9KR (Limpopo, South Africa). Journal of Field Archaeology. 2018; 43(7). pp. 522-537. DOI: 10.1080/00934690.2018.1524219

17. de la Peña P, Val A, Stratford DJ, Colino F, Esteban I, Fitchett JM, Hodgskiss T, Matembo J, Moll R. Revisiting Mwulu's Cave: new insights into the Middle Stone Age in the southern African savanna biome. Archaeological and Anthropological Sciences. 2019; 11(7). pp.3239-3266.

18. Discamps E, Bachellerie F, Baillet M, Sitzia L. 2019 - The use of spatial taphonomy for interpretating Pleistocene palimpstests: an interdisciplinary approach to the Châtelperronian and carnivore occupations at Cassenade (Dordogne, France), PaleoAnthropology, pp. 362-388.

19. Eren MI, Durant A, Neudorf C, Haslam M, Shipton C, Bora J, Korisettar R, Petraglia M. "Experimental Examination of Animal Trampling Effects on Artifact Movement in Dry and Water Saturated Substrates: A Test Case from South India." Journal of Archaeological Science. 2010; 37(12). pp. 3010–3021.

20. Gifford-Gonzalez DP, Behrensmeyer AK. "Observed Formation and Burial of a Recent Human Occupation Site in Kenya." Quaternary Research. 2010; 8(3). pp. 245–266.

21. Gifford-Gonzalez DP. "Ethnoarchaeological Observations of Natural Processes Affecting Cultural Materials." In Explorations in Ethnoarchaeology, edited by R. Gould, 77–101. Albuquerque: University of New Mexico Press. 1978.

22. Isaac G. Toward the interpretation of occupation debris: some experiments and observations, Kroeber Anthopological Society Papers. 1967; 37. pp. 31-57.

23. Lê S, Josse J, Husson F. FactoMineR: An R Package for Multivariate Analysis. Journal of Statistical Software. 2008; 25(1). pp. 1-18.

24. Lenoble A. Ruissellement et formation des sites préhistoriques: référentiel actualiste et exemples d'application au fossile. Oxford, British Archaeological Report International Series. 2005; 1363. 212 p.

25. Lenoble A, Bertran P, Boulogne S, Masson B, Vallin L. Évolution des niveaux archéologiques en contexte périglaciaire. Les nouvelles de l'archéologie. 2009; 118, pp. 16-20.

26. Lenoble A, Texier J-P. Processus géologiques de formation du site d'Isturitz (Sud-Ouest de la France). Paléo. 2016; 27, pp. 235-252.

27. Marwick B, Hayes E, Clarkson C, Fullagar R. "Movement of Lithics by Trampling: An Experiment in the Madjedbebe Sediments, Northern Australia." Journal of Archaeological Science. 2017; 79. pp. 73–85.

28. McPherron SP. Additional statistical and graphical methods for analyzing site formation processes using artifact orientations. PloS One. 2018; 13, 21p.

29. Mourre V. Les industries en quartz au Paléolithique. Terminologie, méthodologie et technologie. Paléo. 1996; 8. pp. 205-223. doi: 10.3406/pal.1996.1160

30. Mourre V. Discoïde ou pas Discoïde? Réflexions sur la pertinence des critères techniques définissant le débitage Discoïde. In: Peresani, M. (éd.), Discoid Lithic Technology -Advances and implications. Oxford, BAR International Series. 2003; 1120. 18 p.

31. Pargeter J. de la Peña P. Milky quartz bipolar reduction and lithic miniaturization: Experimental results and archaeological implications. Journal of Field Archaeology. 2017; 42(6). pp.551-565.

32. Pargeter J, Eren MI. Quantifying and comparing bipolar versus freehand flake morphologies, production currencies, and reduction energetics during lithic miniaturization. Lithic Technology. 2017; 42(2-3). pp.90-108.

33. Patterson LW. Characteristics of bifacial-reduction flake size distribution. American Antiquity. 1990; 55(3). pp. 550-558.

34. Perlès C. Économie des matières premières et économie du débitage: deux conceptions opposes? 25 ans d'Études technologiques en Préhistoire. XIe Rencontres Internationales d'Archéologie et d'Histoire d'Antibes. ADPCA. Juan les-Pins. 1991. pp. 35-45.

35. Petraglia MD, Nash DT. The impact of fluvial processes on experimental sites. In: Nash, D., Petraglia, M. (Eds.). Natural Formation Processes and the Archaeological Record, B.A.R. International series. Oxford. 1987; 352. pp. 108-130.

36. Petraglia MD, Potts R. Water flow and the formation of early Pleistocene artifact sites in Olduvai Gorge, Tanzania. Journal of Anthropological Archaeology. 1994; 13. pp. 228-254.

37. Rodríguez-Rellán C. La anisotropía y el clivaje del cuarzo automorfo y sus posibles efectos sobre la talla: Una revisión bibliográfica. Journal of Lithic Studies. 2015; 2(2). pp.189-207.

38. Rodríguez-Rellán C. Variability of the rebound hardness as a proxy for detecting the levels of continuity and isotropy in archaeological quartz. Quaternary International. 2016; 424. pp.191-211.

39. Schick KD. Sites in the Making, Experiments in the Formation and Transformation of Archaeological Occurrences. Oxford. BAR International Series. 1986; 319. 313 p.

40. Shea JJ, Prehistoric stone tools of Eastern Africa: A guide. Cambridge University Press. 2020.

41. Shott MJ. Size and form in the analysis of flake debris: review and recent approaches. Journal of Archaeological Method and Theory. 1994; 1(1). pp. 69-110.

42. Schiffer MB. Toward the identification of Formation Processes. Society for American Archaeology. 1983; 48(4), pp. 675-706.

43. Sitzia L. Chronostratigraphie et distribution spatiale des dépôts éoliens quaternaires du basin aquitain. Université Sciences et Technologies, Bordeaux I. 2014. 344 p.

44. Stahle DW, Dunn JE. 1982 - An analysis and application of the size distribution of waste flakes from the manufacture of bifacial stone tools. World Archaeology 14 (1), pp. 84-97.

45. Staurset S, Coulson S. Sub-surface movement of stone artefacts at White Paintings Shelter, Tsodilo Hills, Botswana: Implications for the Middle Stone Age chronology of central southern Africa. Journal of Human Evolution. 2014; 75, pp. 153-165.

46. Stout D. Stone toolmaking and the evolution of human culture and cognition. Philosophical Transactions of the Royal Society B: Biological Sciences, 2011; 366(1567). pp.1050-1059.

47. Sunagawa I. Crystals. Growth, Morphology and Perfection, Interfaces. Cambridge University Press. New York. 2005.

48. Terradas X. Discoid flaking method: conception and technological variability. In: Peresani, M. (Ed.), Discoid Lithic Technology -Advances and implications. Oxford, BAR International Series. 2003; 1120. pp. 19-32.

49. Texier J-P. À propos des processus de formation des sites préhistoriques. Paléo. 2000; 12. pp. 379-386.

50. Thomas M, Gravina B. Analyse techno-économique des vestiges lithiques de la zone B de l'abri inférieur du Moustier (Dordogne, France). Paléo. 2019; 30-1. pp. 300-317.

51. Thomas M, Discamps E, Gravina B, Texier J-P. Analyse taphonomique et spatiale de palimpsestes d'occupations moustériennes de l'abri inférieur du Moustier (Dordogne, France). Paléo. 2019; 30-1. pp. 278-299.

52. Todisco D, Bhiry N, Desrosiers P. Paleoeskimo Site Taphonomy: An Assessment of the Integrity of the Tayara Site, Qikirtaq Island, Nunavik, Canada, Geoarchaeology: An International Journal. 2009; 24(6). pp. 743–791

53. Tryon CA, McBrearty S, Texier PJ. Levallois lithic technology from the Kapthurin formation, Kenya: Acheulian origin and Middle Stone Age diversity. African Archaeological Review. 2005, 4, pp. 199-229.

54. Villa P. Conjoignable pieces and site formation processes. American Antiquity. 1982; 47. pp. 276-290.

55. Villa P, Courtin J. The Interpretation of Stratified Sites: A View from Underground. Journal of Archaeological Science. 1983; 10, pp. 267-281.

56. Weaver TD, Boyco RH, Steele TE. Cross-platform program for likelihood-based statistical comparisons of mortality profiles on a triangular graph. Journal of Archaeological Science. 2011; 38. pp. 2420-2423.

57. Yellen JE. Archaeological Approaches to the Present: Models for Reconstructing the Past. Vol. 1. New York: Academic Press. 1977.

Click here to access/download;Figure;fig. 1.jpg

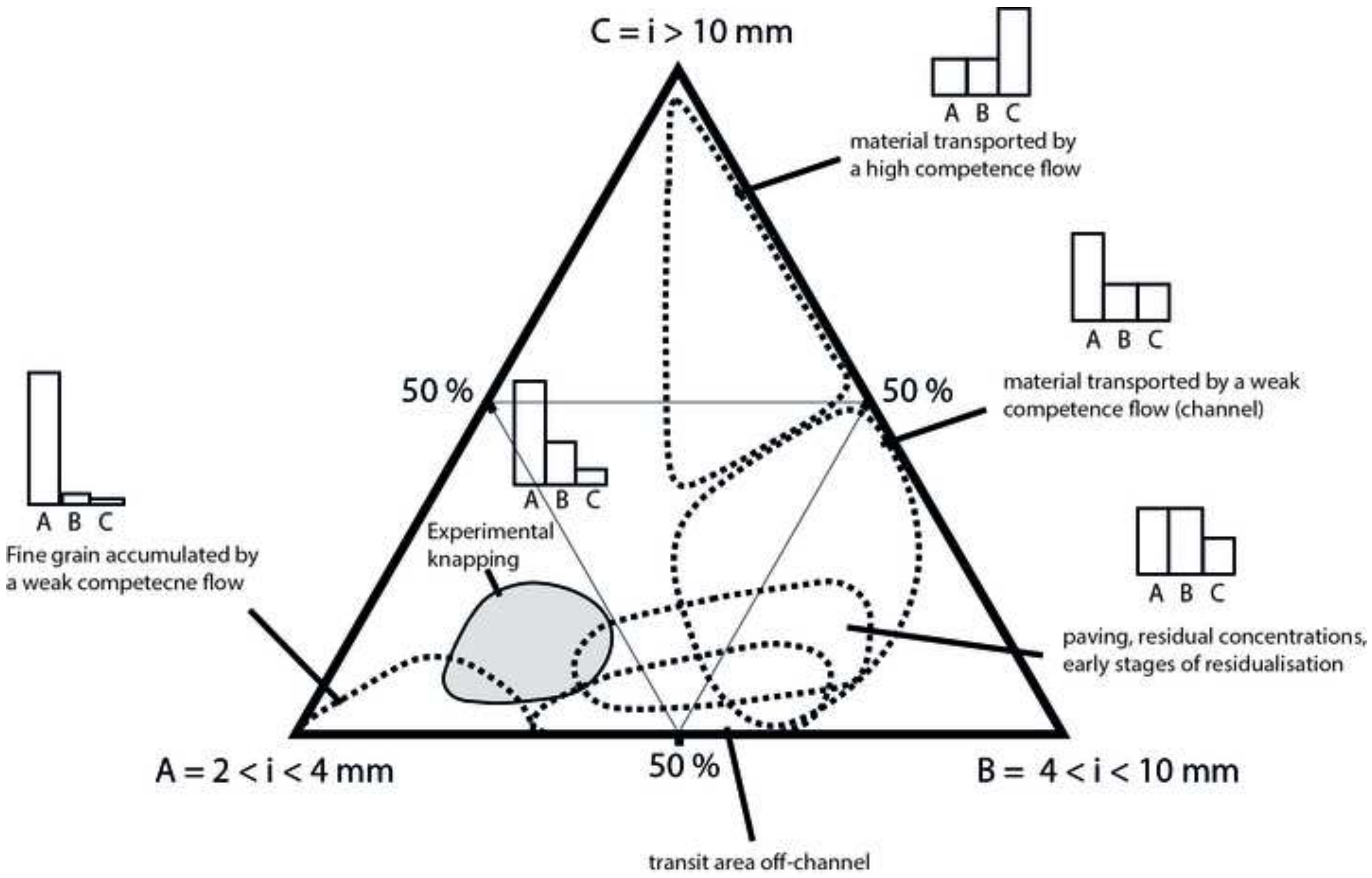

Click here to access/download;Figure;fig. 2.jpg

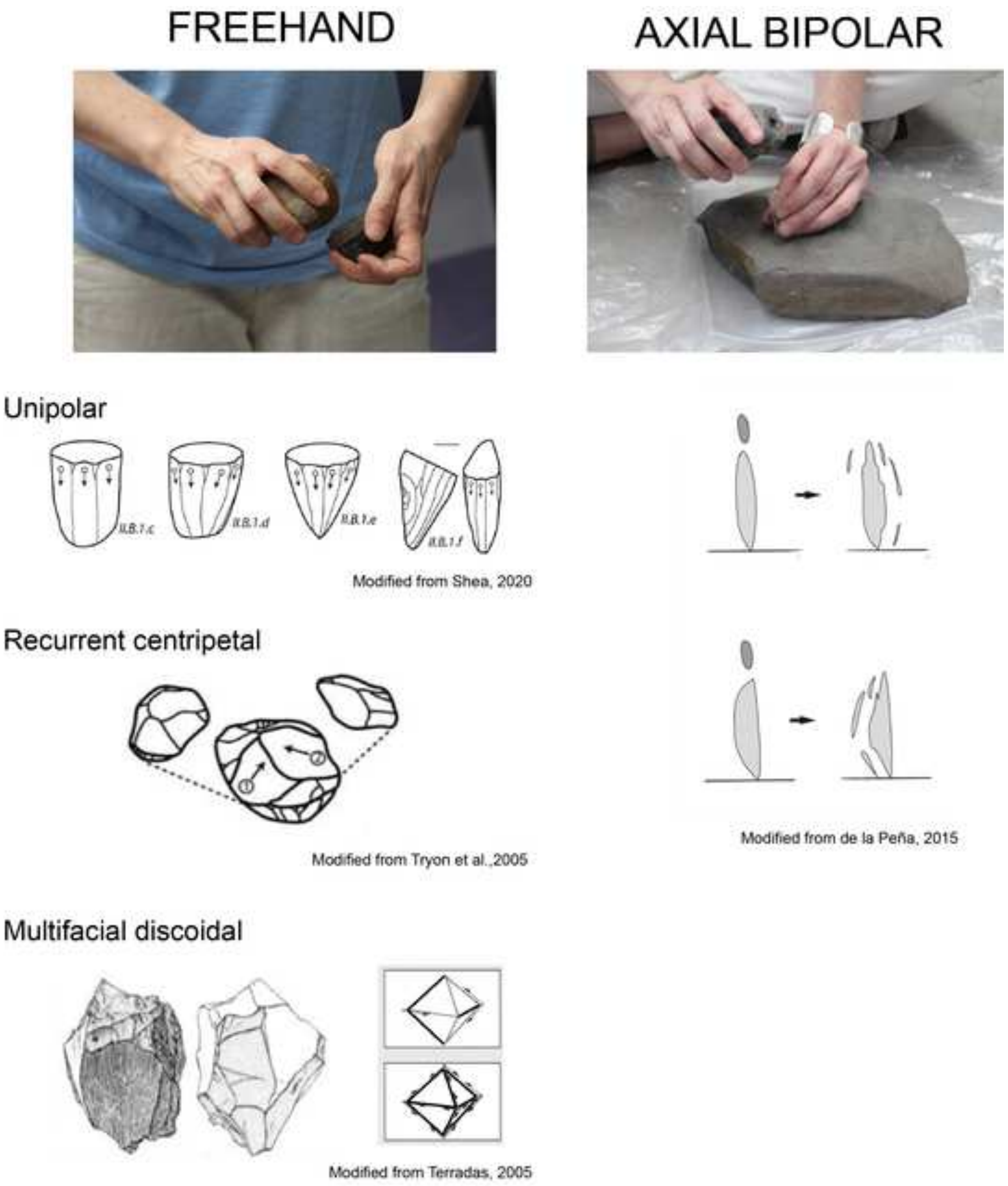

Click here to access/download;Figure;fig. 3.jpg

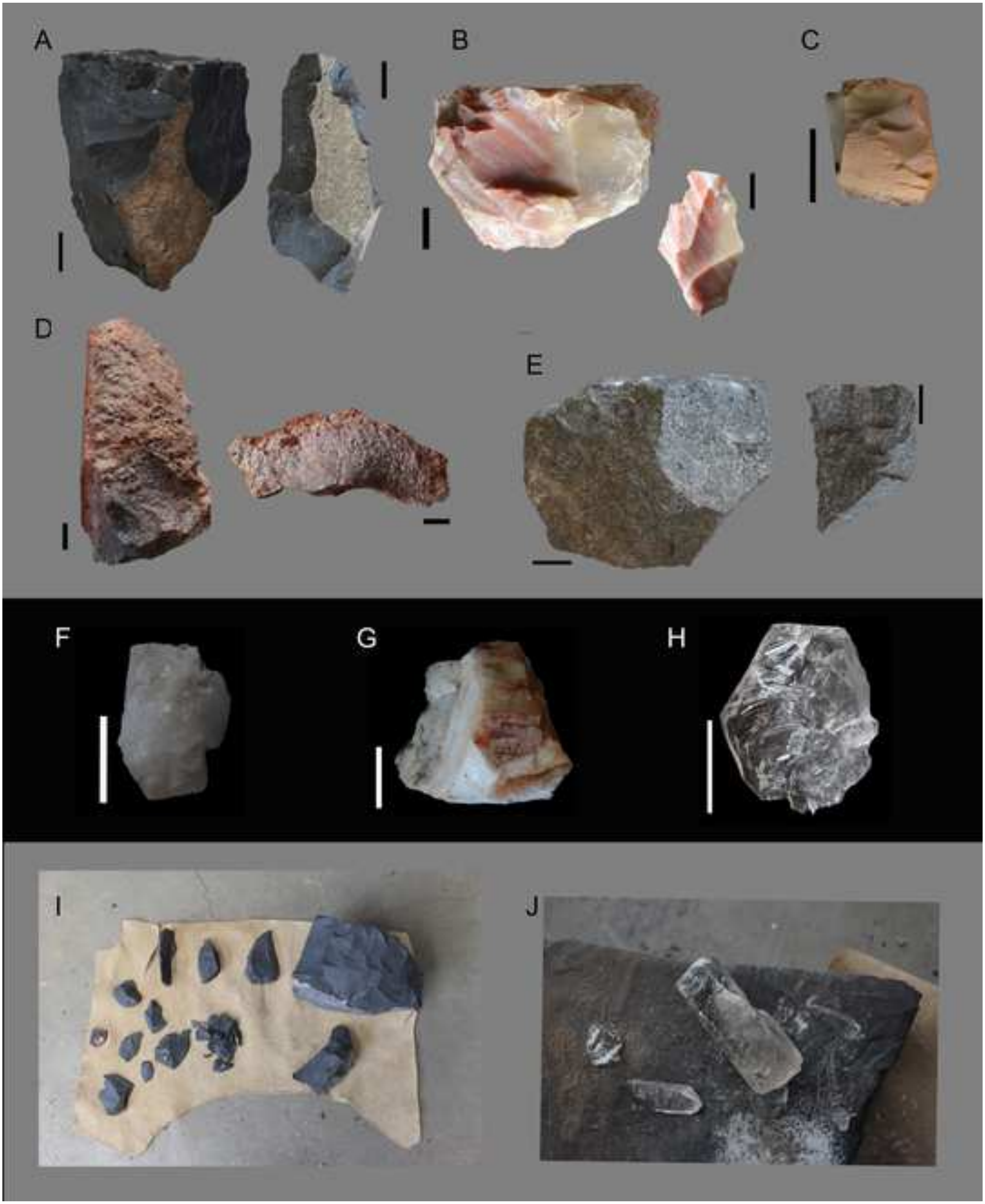

Click here to access/download;Figure;fig. 4.jpg

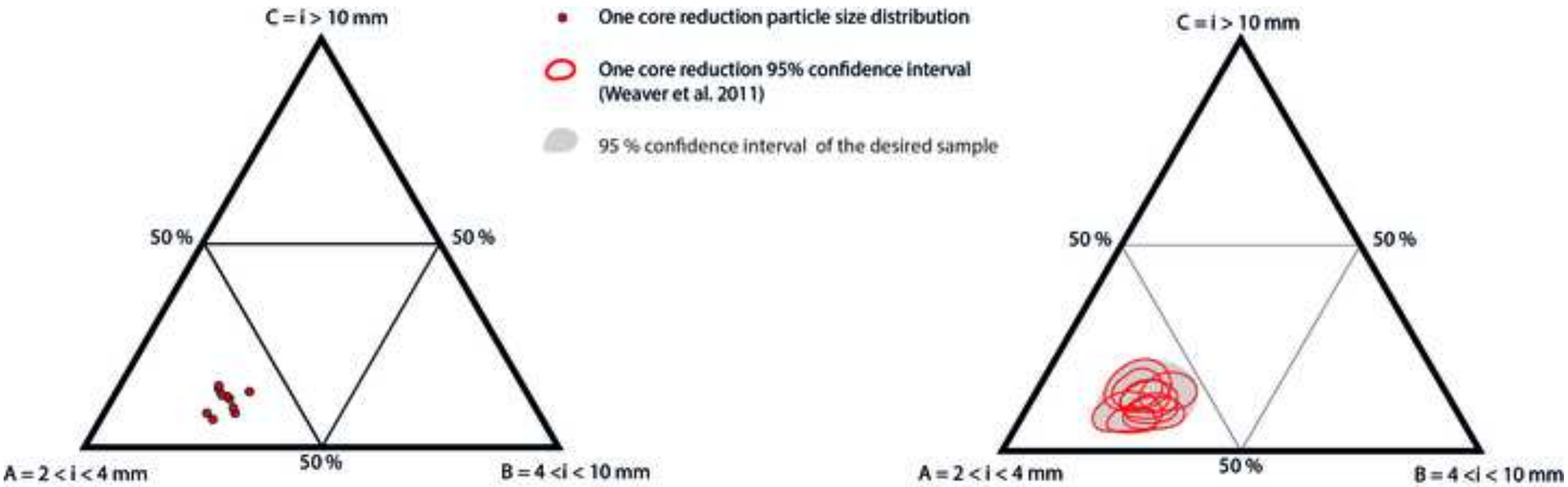

Click here to access/download;Figure;Fig5.tif

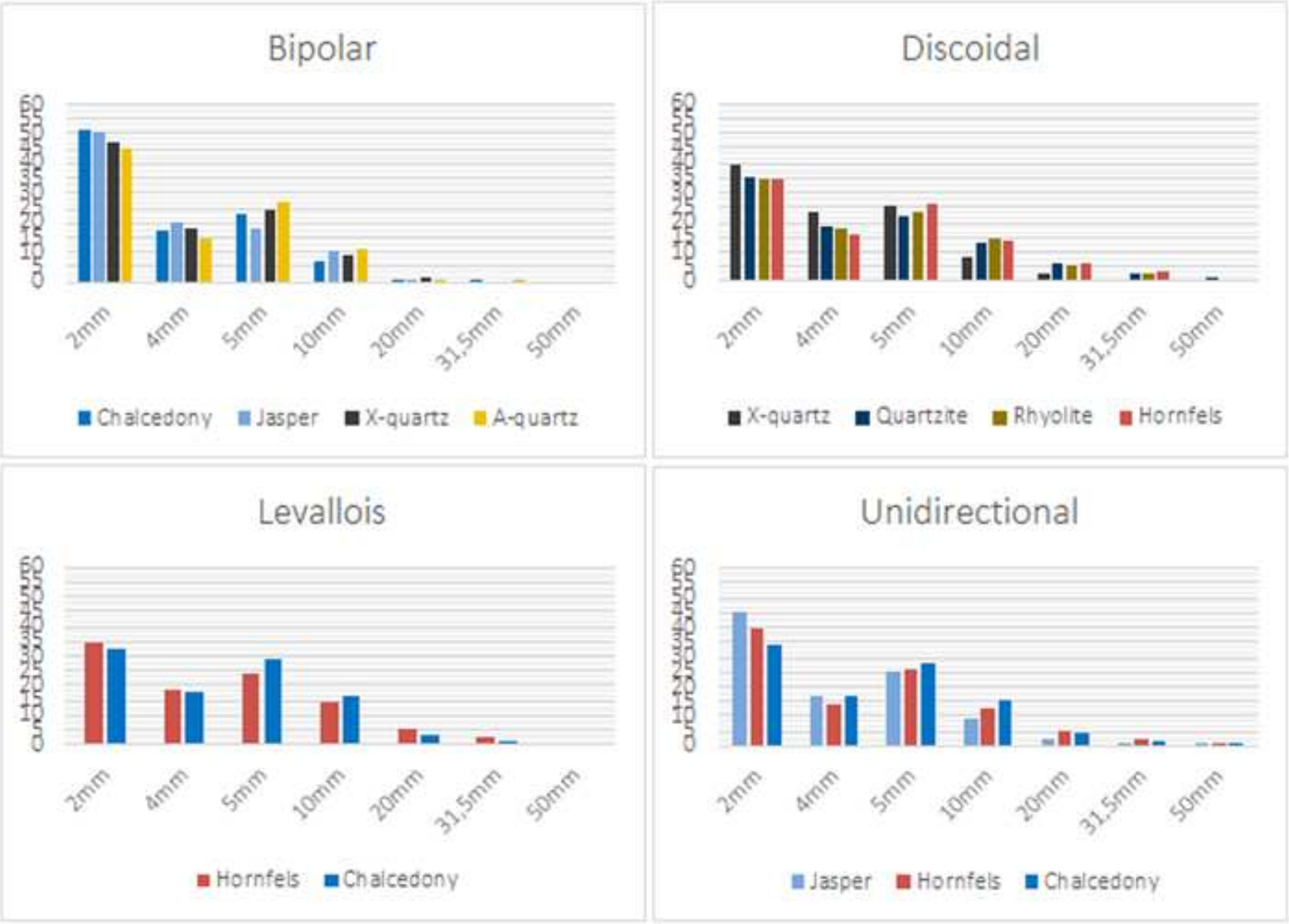

Click here to access/download;Figure;fig. 6.jpg

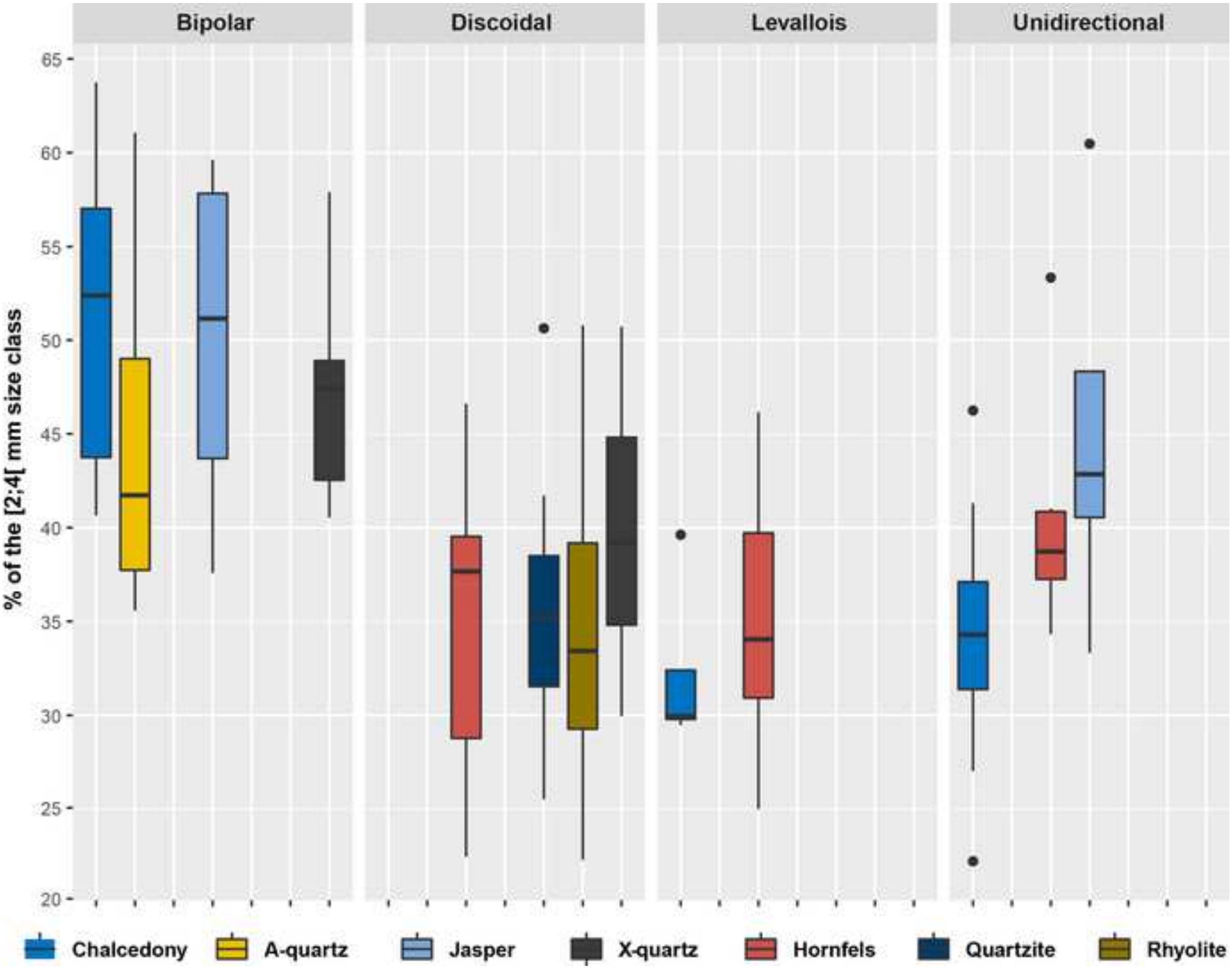

Click here to access/download;Figure;fig. 7.jpg

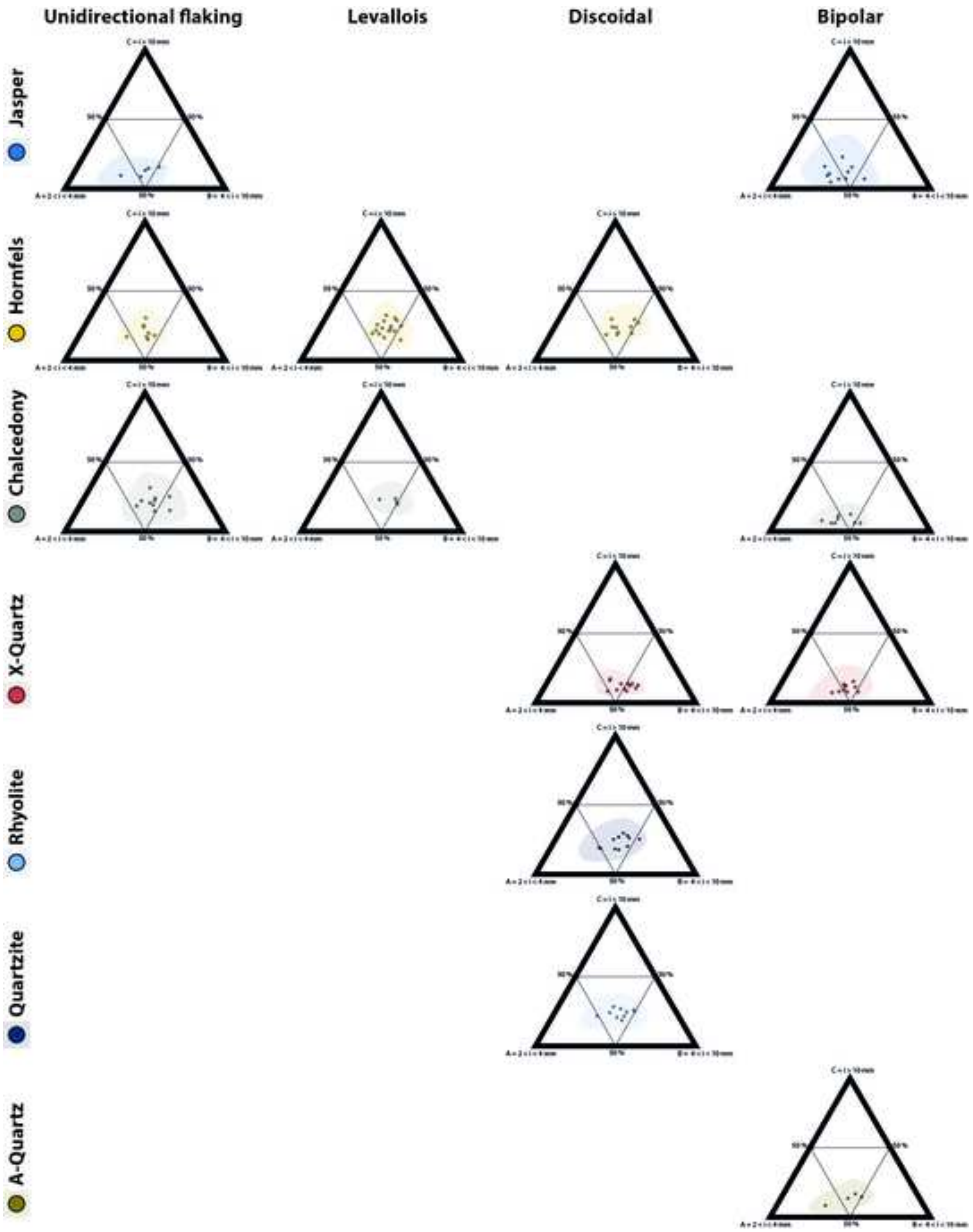

Click here to access/download;Figure;fig. 8.jpg

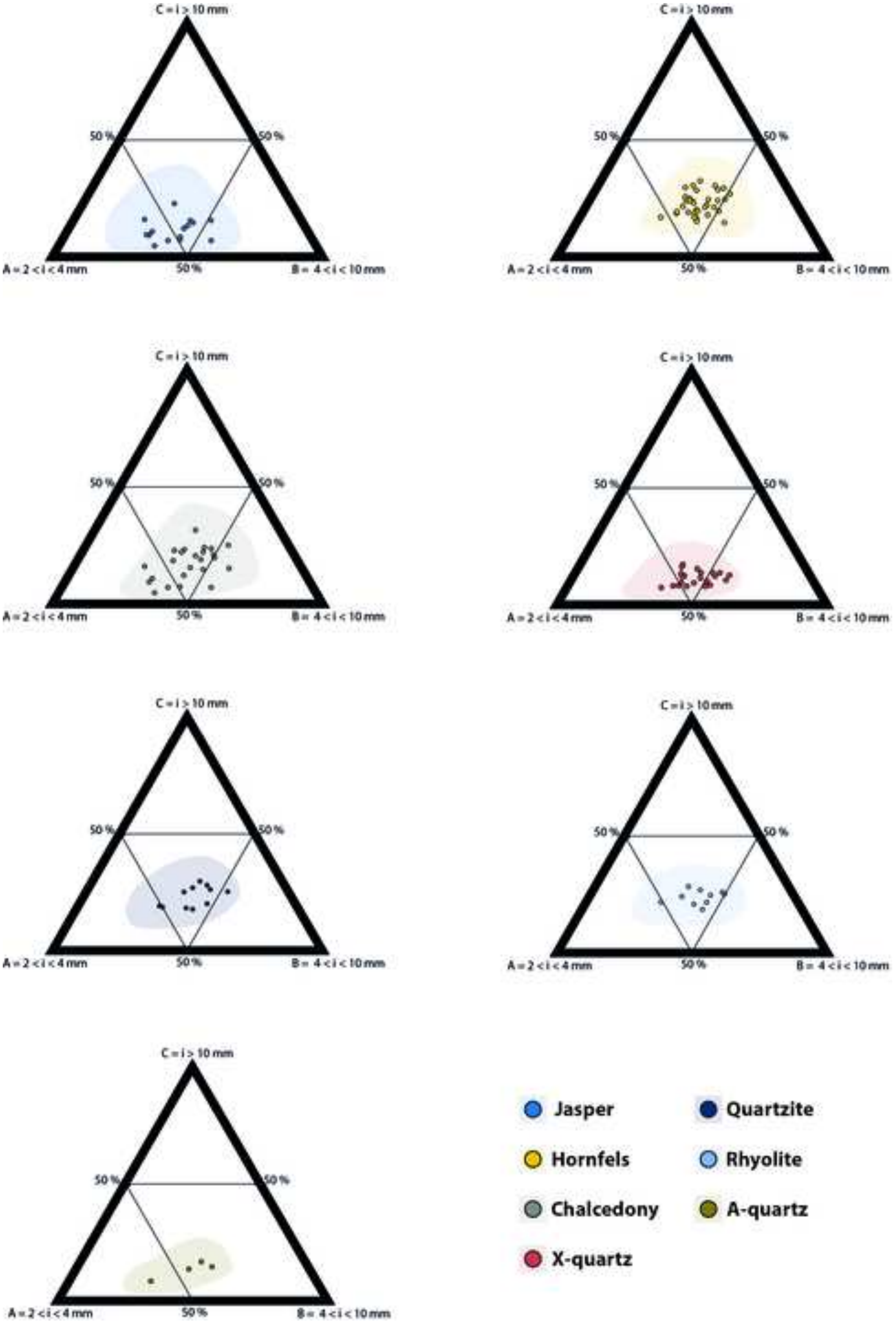

Click here to access/download;Figure;fig. 9.jpg

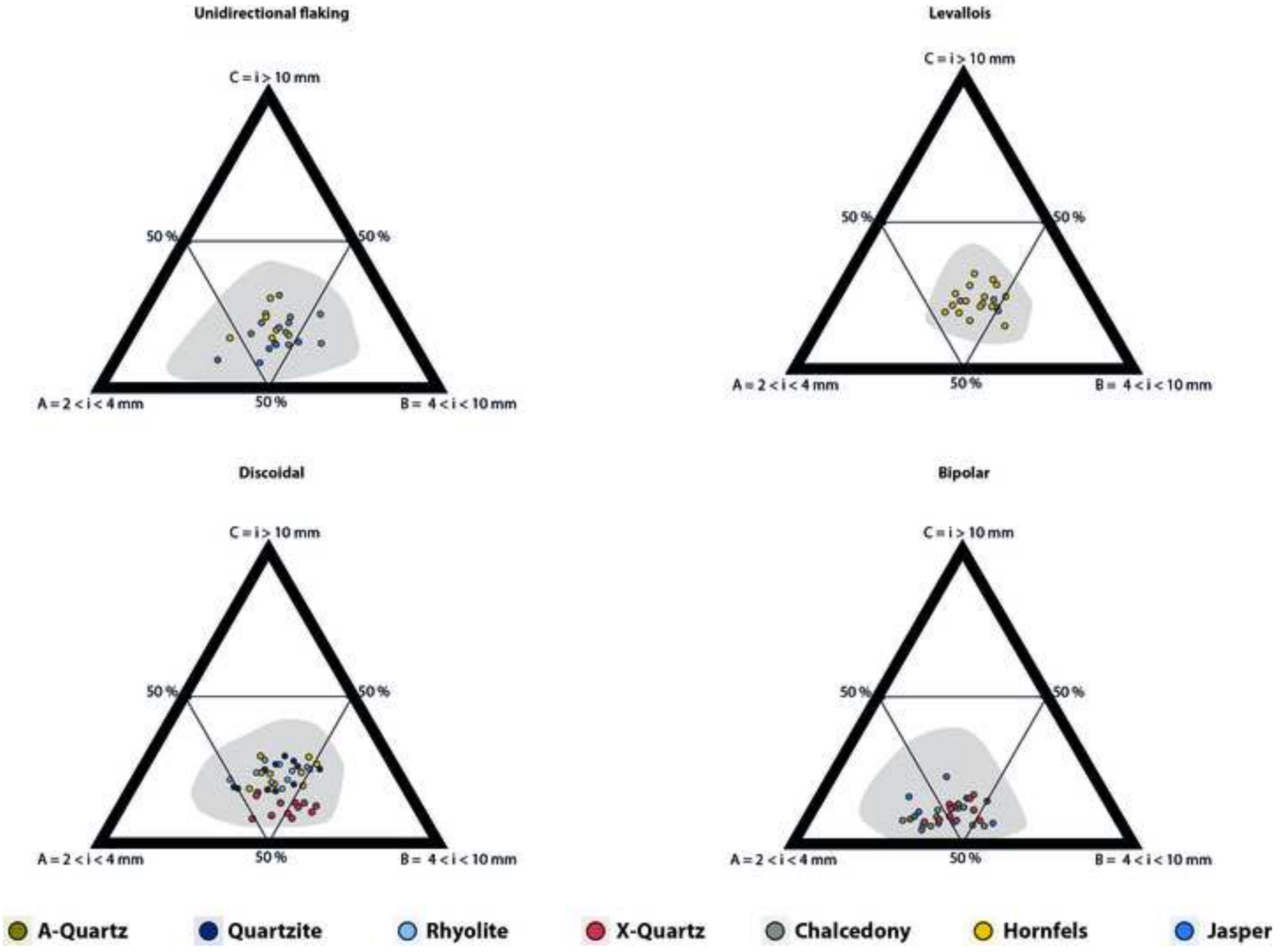

Click here to access/download;Figure;fig. 10.jpg

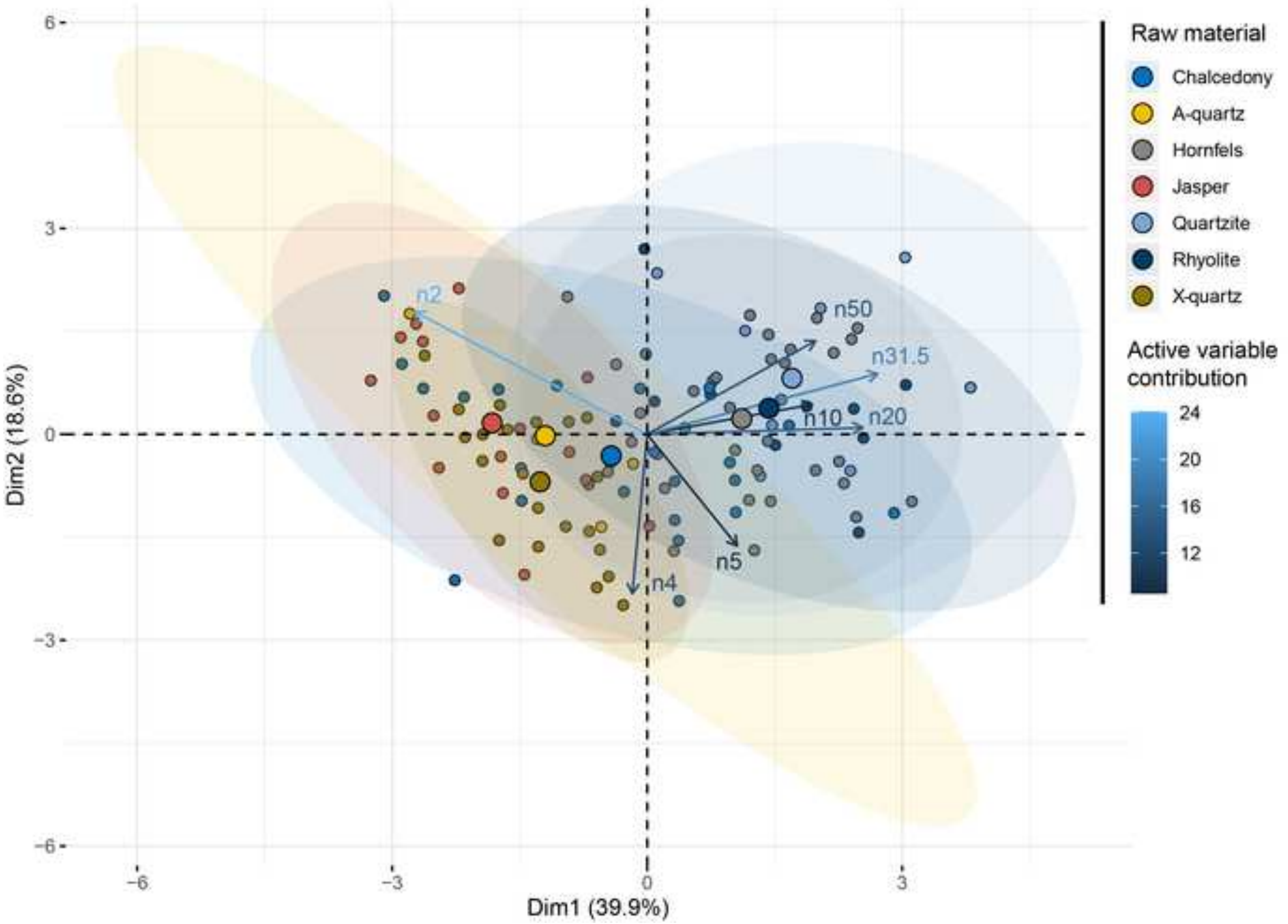

Click here to access/download;Figure;fig. 11.jpg

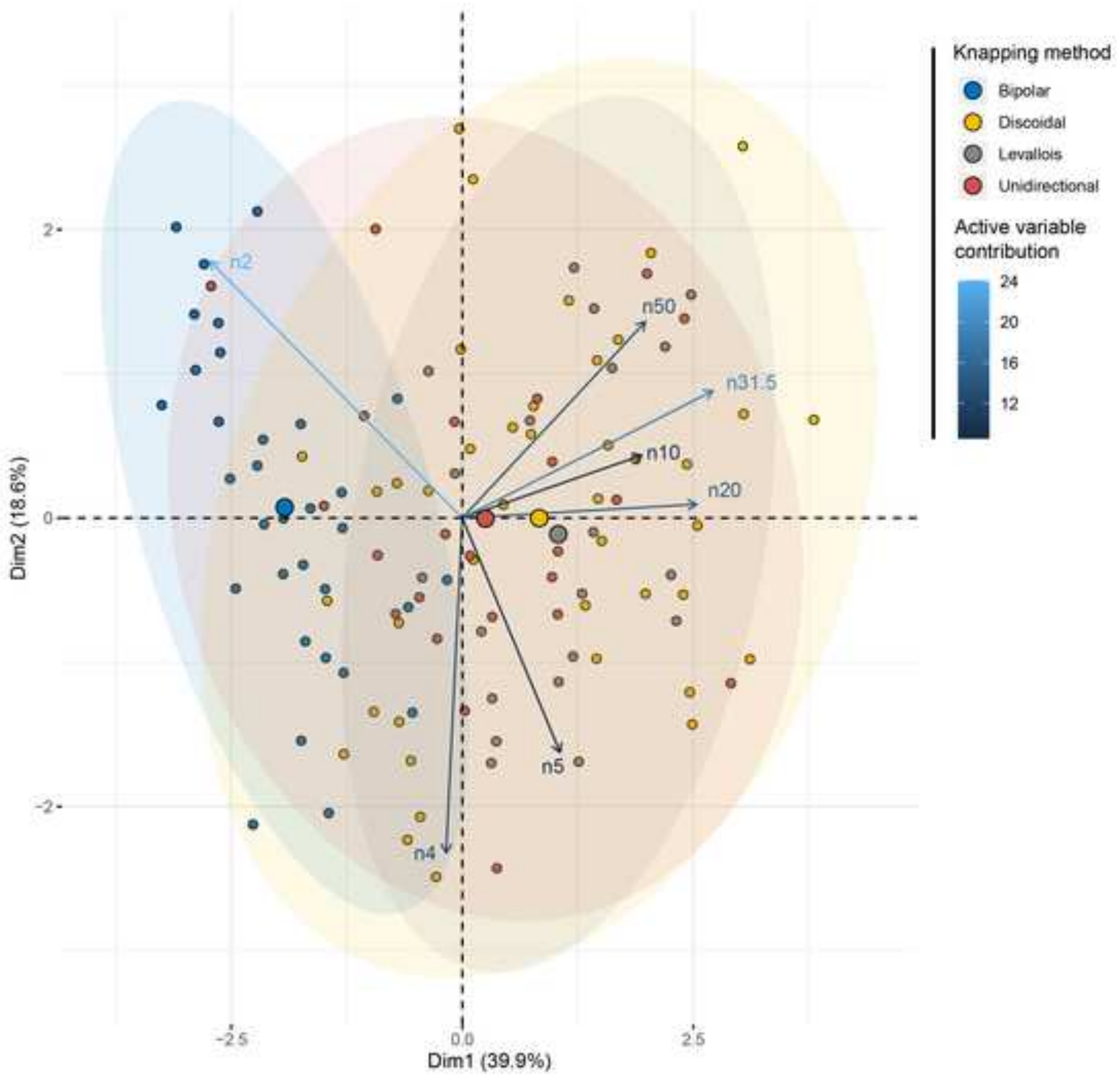

Click here to access/download;Figure;fig. 12.jpg

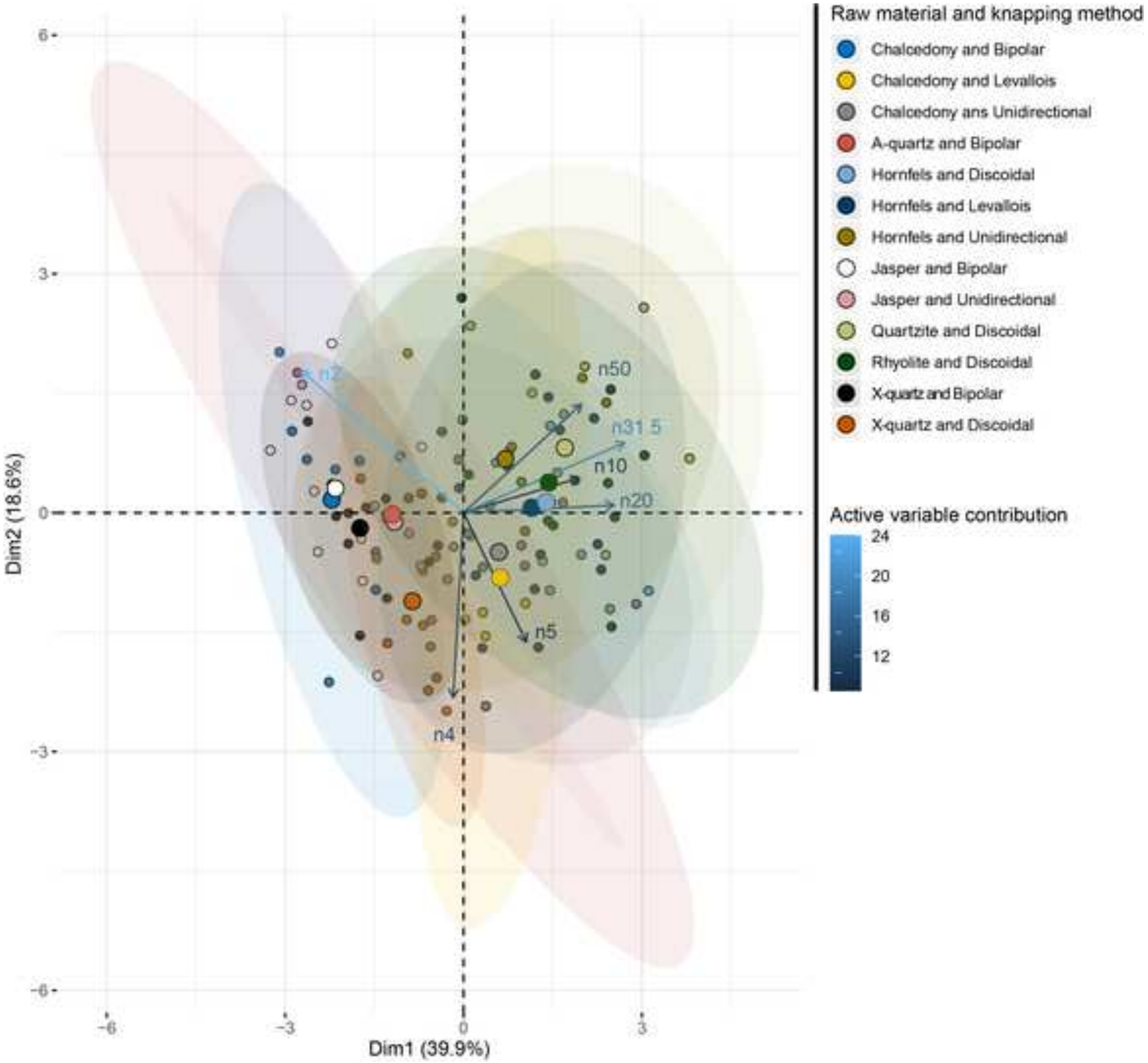

Click here to access/download;Figure;fig. 13.jpg

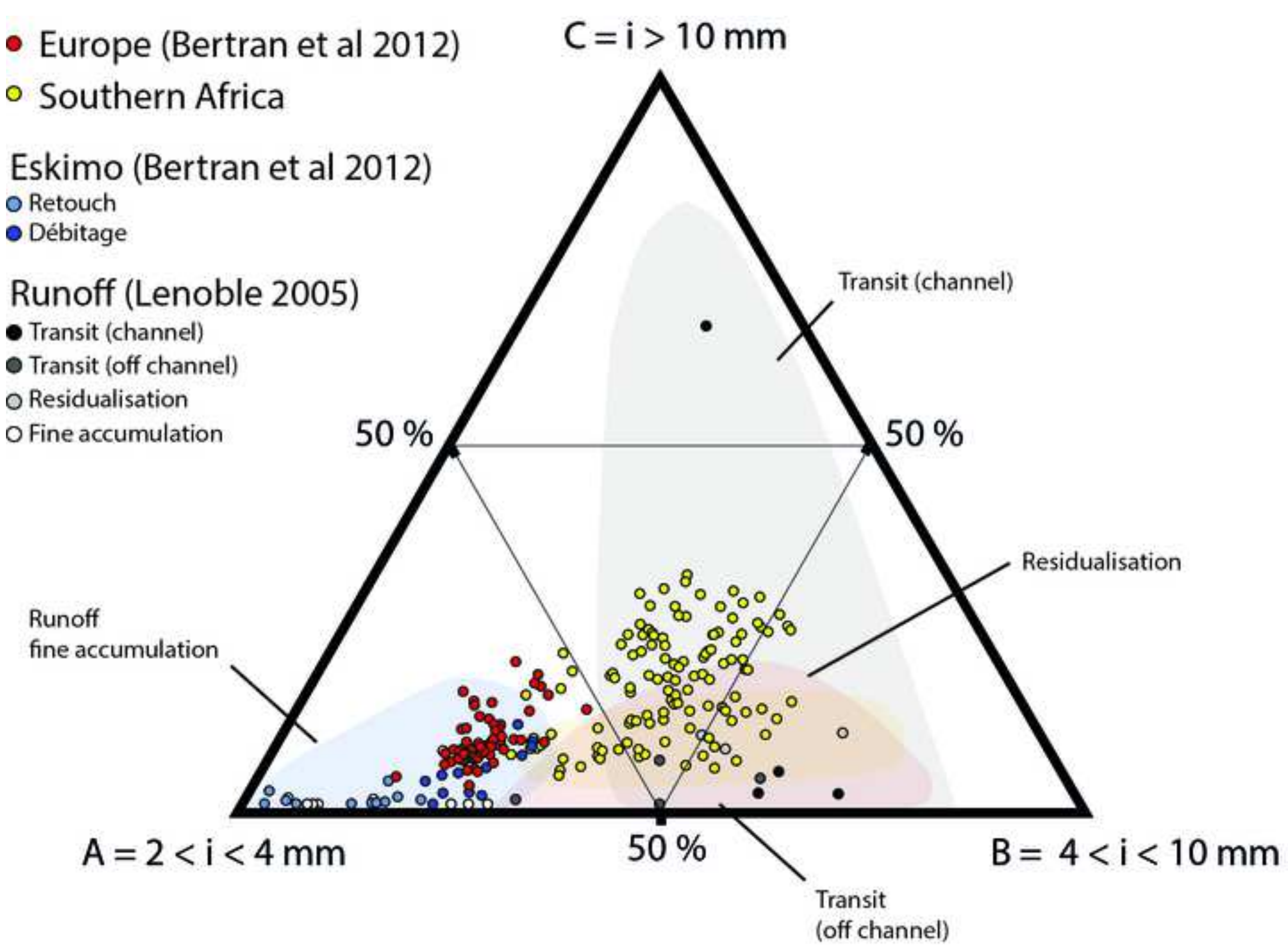

Click here to access/download;Figure;fig. 14.jpg

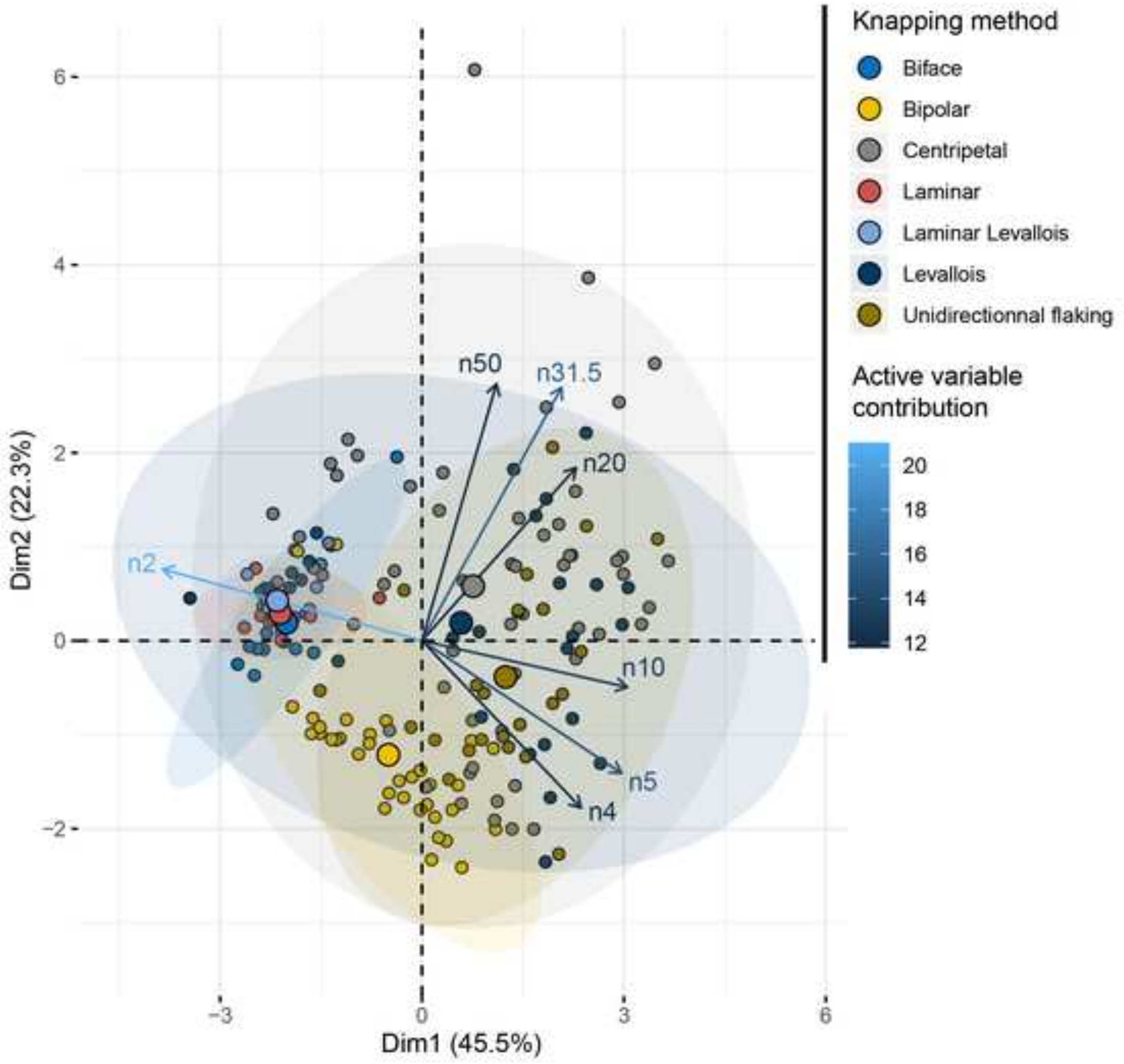

Click here to access/download;Figure;fig. 15.jpg

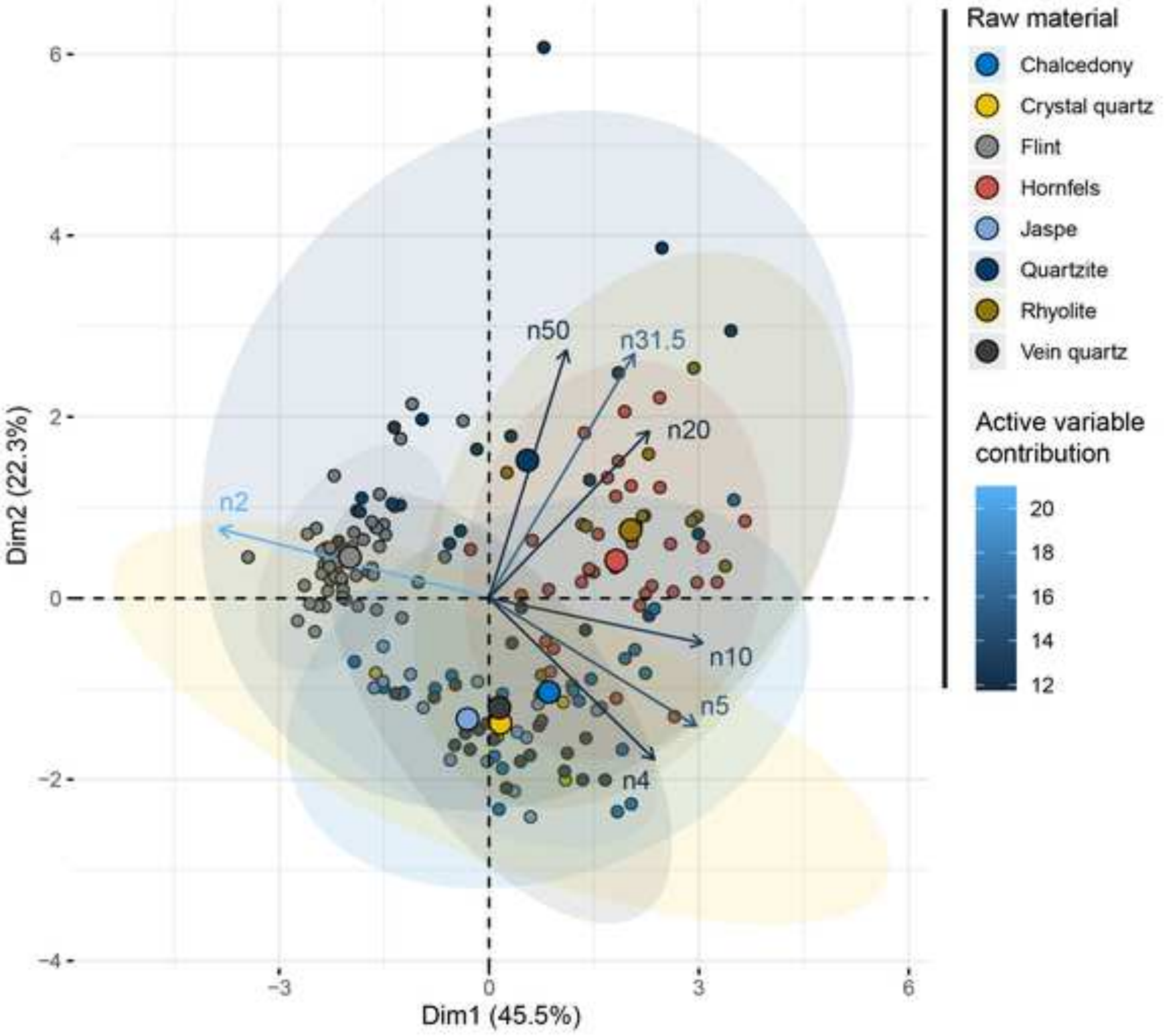

Click here to access/download;Figure;fig. 16.jpg

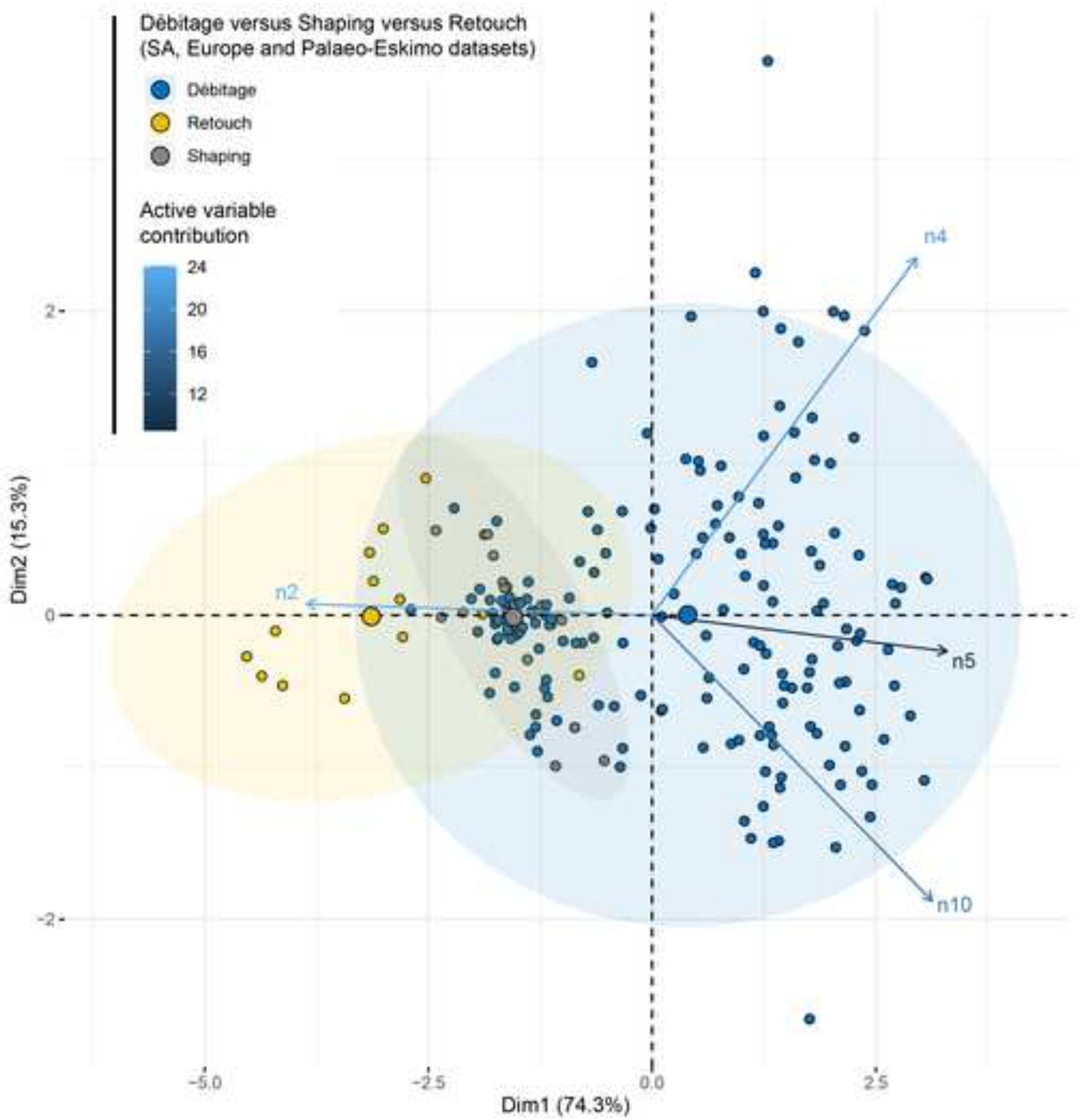

Click here to access/download;Figure;fig. 17.jpg

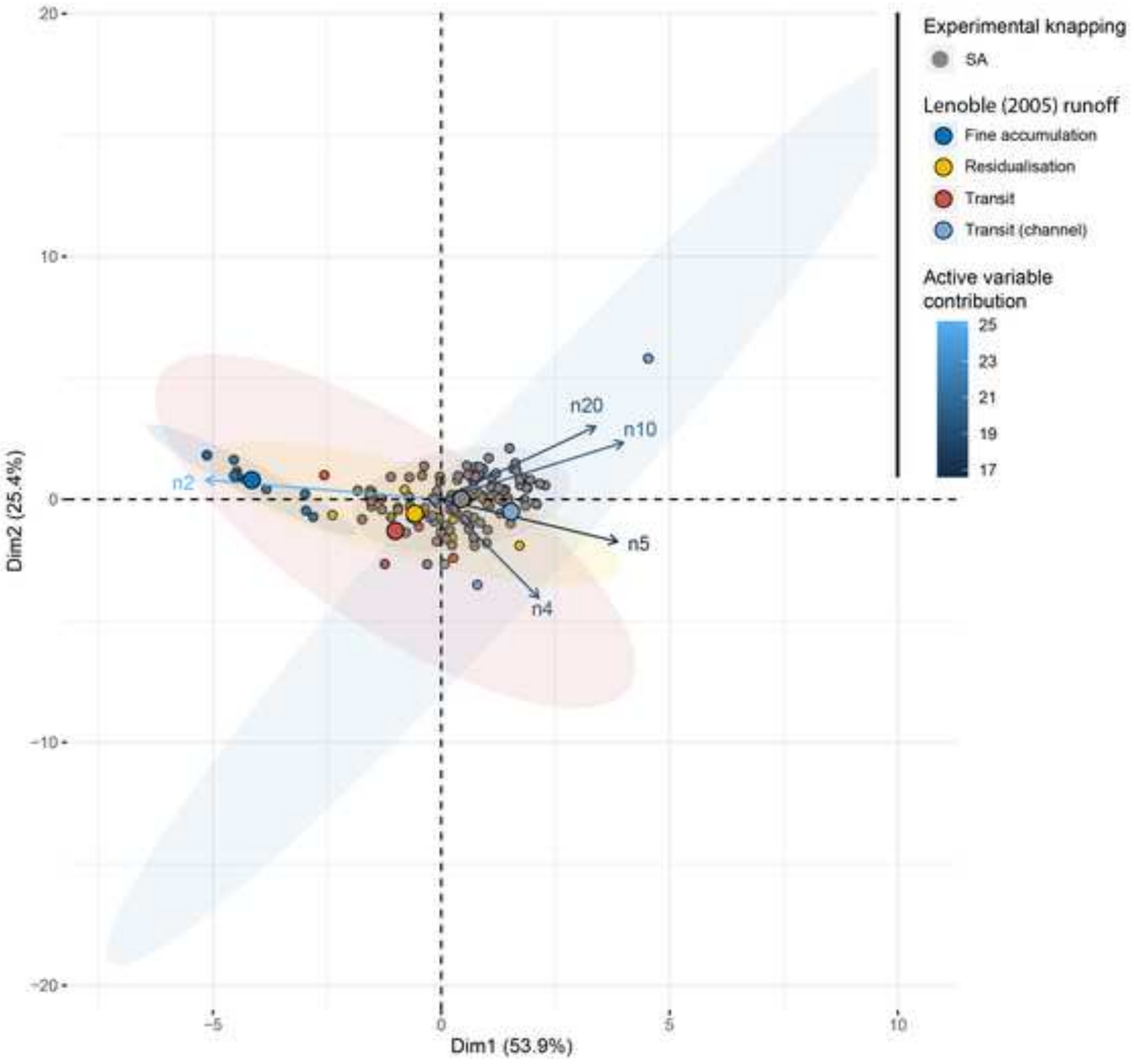

Supplement: Attachment — Submitted filename: PONE-D-22-10277_reviewer_REVn.pdf [file pone.0278867.s005.pdf]
