# Supplementary material for: Particle size distribution: An experimental study using southern African reduction methods and raw materials
Source: PLoS One. 2022 Dec 30;17(12):e0278867. doi: 10.1371/journal.pone.0278867 (PMC9803312; doi:10.1371/journal.pone.0278867)
Supplement: Attachment — Submitted filename: PONE-D-22-10277_Rev.pdf [file pone.0278867.s007.pdf]

PLOS ONE

Particle size distribution: an experimental study using southern African reduction methods and raw materials

--Manuscript Draft--

**Manuscript Number:**	PONE-D-22-10277R1
**Article Type:**	Research Article
**Full Title:**	Particle size distribution: an experimental study using southern African reduction methods and raw materials
**Short Title:**	Particle size distribution
**Corresponding Author:**	Paloma de la Peña, Ph.D<br>Universidad de Granada<br>Granada, SPAIN
**Keywords:**	Lithics, site formation, Experimental Archaeology, particle size analysis
**Abstract:**	We experimentally created a particle size dataset that is based on reduction sequences and raw materials typical of the Middle and Later Stone Age in southern Africa. The reason for creating this new dataset is that current particle size frameworks are based, almost exclusively, on flint and western European knapping methods. We produced the dataset using knapping methods and raw materials frequently encountered in the southern African archaeological record because we wanted to test whether it has the same distribution as particle size datasets experimentally created in Europe, and to initialise the production of a database for use in the analysis of lithic assemblages from southern African Late Pleistocene deposits. We reduced 117 cores of quartz, quartzite, jasper, chalcedony, hornfels, and rhyolite. The knapping methods selected were unidirectional, discoidal , Levallois recurrent and bipolar flaking. In this article we compare this new particle size distribution dataset with the results obtained from previous experiments. We found that the southern African dataset shows a wider size range distribution, which seems to be explained by differences in knapping methods and raw materials. Our results show that there is overlap between the distribution of the southern African experimental knapping dataset and the sorting experiment conducted by Lenoble on flint artefacts in a runoff context. This article shows that a particle size analysis is not sufficient on its own to assess the perturbation of an archaeological assemblage and must be coupled with other analytical tools .
**Order of Authors:**	Paloma de la Peña, Ph.D
	Marc Thomas
	Tumelo Rufus Molefyane
**Response to Reviewers:**	Dear Editor,<br>Thank you for letting us revise the manuscript. We are also grateful for the constructive comments of the two reviewers. We have addressed all the comments and problems highlighted in the review.<br>Yours sincerely,<br><br>Dr. Paloma de la Peña<br>In Johannesburg, 11th August 2022<br><br><br>Response to reviewers<br><br>Reviewer #1<br>Have the authors made all data underlying the findings in their manuscript fully available?<br>We incorporated our database into the supplementary material (SM1) and added some statistical information on the implementation of PCA (SM2) and an R script that allows us to reproduce all PCA analyses and obtain more statistics (SM3).

Powered by Editorial Manager® and ProduXion Manager® from Aries Systems Corporation

Is the manuscript presented in an intelligible fashion and written in standard English?
The manuscript was revised in the first and second submission by Dr Tammy Hodgskiss and in the second revision by Dr Lucinda Backwell, both of them are native English-speaking persons very familiar with archaeology vocabulary as they regularly publishes on Middle and Later Stone Age Archaeology.

My biggest criticism concerns the methodical setup of the experimental knapping. There is little explanation or context for what the initial set-up was: Why were 117 cores knapped (how to get to that number)? How was the distribution across raw materials and reduction methods arrived at? Was there any bigger experimental design behind it? Looking at the distribution of cores in Table 1, it honestly looks a bit unsystematic. The reasoning that not all knapping methods were not applied to all raw materials due to the Southern African archaeological context (lines 210-212) is not convincing, as there is evidence for all of the knapping methods on all raw materials of this study (admittedly much rarer for some raw materials, e.g. Levallois on quartz is rare). This then also compromises some statistical analyses of the study as the authors themselves acknowledge (lines 213-214). Ideally, the study would incorporate some more reduction of different raw materials/knapping methods or be at the very list much clearer about the description of the study design in the Materials and Methods section!

Thank you for this comment. The reviewer is right that the experiment seems a bit unsystematic. The number of experiments is odd because if the core fragmented during the knapping process, we did not include those experiments. Moreover, it has also been influenced by the covid pandemic. The experiment was done in late 2020 and early 2021 when it was not possible to access raw material outcrops due to driving and transport restrictions, so we operate with the material available at the laboratory of Archaeology at the Evolutionary Studies Institute.
Finally, the reason for some raw materials and knapping varieties not represented is that there are some knapping methods that are not reported in the archaeological record (Middle and Later Stone Age). For instance, to the best of our knowledge there is no evidence of Levallois recurrent centripetal reduction on automorphic and xenomorphic quartz or, on the other hand, there is no documentation on bipolar knapping on hornfels. It would have no archaeological sense to include those varieties in the experimental collection.
This has been clarified in the presentation of the methodology experimentation.

While the results are well-done and well-presented, the discussion is somewhat lacking substance and not yet sufficient for publication. Most importantly, the authors should at least provide one test application (i.e. case study) showing the value of their experimental study and the particle size distribution in one MSA or LSA assemblage from southern Africa. This would clearly show the analytical value of the current study for archaeology, which is so far only assumed but not shown. Particularly, this could show with empirical data why their database and not the EU dataset should be used for such an analysis. Additional discussion on the potential insights that can be gained from the experimental database for MSA/LSA studies from a general potential would also much add to the relevance of the current study.
We have reinforced and re-done the discussion adding new ideas and an in depth criticism of our results (see the changes in the final section)
We think it is a very good idea to compare our experimental results with archaeological collections and, indeed, we would like to do another research paper only for that comparison. We wanted first to publish the experimental material and to compare it to other experimental studies such as Schick (1986, 1987) or Bertran et al. (2006, 2012), which are valuable contributions for African and European assemblages respectively.
Finally, some parts of the manuscript such as the Introduction (lines 48-54) and several parts later om need some further polishing of the English language, potentially by the additional help of a native speaker.
The manuscript was revised in the first and second submission by Dr Tammy Hodgskiss and in the second revision also by Dr Lucinda Backwell, both of them are native English speaking persons very familiar with archaeology vocabulary as they regularly publishes on Middle and Later Stone Age Archaeology.

Reviewer #2

The paper would benefit of a more accurate description of the reason why a South

Powered by Editorial Manager® and ProduXion Manager® from Aries Systems Corporation

	African dataset has been compared with European datasets.<br><br>The authors assess that particle size distribution variability is strongly affected by raw materials and knapping methods, however it is not clearly explained if to demonstrate this diversity was the main reason behind their choice to perform comparative analyses between different contexts and different raw materials. I think that much benefit will be provided by clarifying since the beginning of the paper what is the main purpose of this research.<br>It is true that raw material variability strongly affects the results of these type of analysis, thus my question is if it is worth it to compare different débitage methods performed on different raw materials from different geographical contexts. It would be useful if the authors will dedicate a section in the paper to explain this main decision.<br>The only available datasets made thanks to the Bertran et al methodology describe mainly European archaeological context. That was the main reason of this comparison and doing these experiments and the datasets on particle size. Moreover, particle size analysis in southern Africa archaeological collections have not been applied before. To have a valid framework it needs to be addressed from the most frequent raw materials and the knapping methods in this chronology.<br>The main purpose of the paper is to offer a framework to understand the particle size distribution on Southern African sites of the Late Pleistocene. Following this reviewer advice we have reinforced this in the manuscript (see the abstract, the introduction and the "Questions and objectives" sections.<br><br>Furthermore, the statistical analyses are performed in the correct way. However, it is quite difficult to navigate through the PCA results without knowing any information regarding the loadings and the scores that each principal component provides. In this case, the graphs alone it is not enough to understand the degree of variance of the analyzed variables.<br>I would appreciate an additional table with variables used for comparative analyses, including the variables used from the datasets from previous experimental analyses. At this point, there are no information regarding the normalization of the data between the different datasets<br><br>We have included the statistics information associated to the PCA in SM 2 and 3.<br>We have also included in SM1 the datasets of previous experimental analyses together with our own experimentation, everything studies and formatted in the same way (sieving with the same screen mesh for at least 4 size class).<br><br>...<br>We have also modified figures 1, 6 and 14 as there were some typos.<br>Finally, we would like to change our title to: Particle size distribution: an experimental study using southern African reduction methods and raw materials
**Additional Information:**	
**Question**	**Response**
**Financial Disclosure**<br><br>Enter a financial disclosure statement that describes the sources of funding for the work included in this submission. Review the submission guidelines for detailed requirements. View published research articles from *PLOS ONE* for specific examples.<br><br>This statement is required for submission and **will appear in the published article** if the submission is accepted. Please make sure it is accurate.	PdlP received CoE-NRF (South Africa) funding

Powered by Editorial Manager® and ProduXion Manager® from Aries Systems Corporation

**Unfunded studies**<br>Enter: *The author(s) received no specific funding for this work.*<br><br>**Funded studies**<br>Enter a statement with the following details:<br>• Initials of the authors who received each award<br>• Grant numbers awarded to each author<br>• The full name of each funder<br>• URL of each funder website<br>• Did the sponsors or funders play any role in the study design, data collection and analysis, decision to publish, or preparation of the manuscript?<br>• **NO** - Include this sentence at the end of your statement: *The funders had no role in study design, data collection and analysis, decision to publish, or preparation of the manuscript.*<br>• **YES** - Specify the role(s) played.<br><br>* typeset	
**Competing Interests**<br><br>Use the instructions below to enter a competing interest statement for this submission. On behalf of all authors, disclose any competing interests that could be perceived to bias this work—acknowledging all financial support and any other relevant financial or non-financial competing interests.<br><br>This statement is required for submission and **will appear in the published article** if the submission is accepted. Please make sure it is accurate and that any funding sources listed in your Funding Information later in the submission form are also declared in your Financial Disclosure statement.<br><br>View published research articles from *PLOS ONE* for specific examples.	The authors have declared that no competing interests exist.

**NO authors have competing interests**<br><br>Enter: *The authors have declared that no competing interests exist.*<br><br>**Authors with competing interests**<br><br>Enter competing interest details beginning with this statement:<br><br>*I have read the journal's policy and the authors of this manuscript have the following competing interests: [insert competing interests here]*<br><br>* typeset	
**Ethics Statement**<br><br>Enter an ethics statement for this submission. This statement is required if the study involved:<br><br>• Human participants<br>• Human specimens or tissue<br>• Vertebrate animals or cephalopods<br>• Vertebrate embryos or tissues<br>• Field research<br><br>Write "N/A" if the submission does not require an ethics statement.<br><br>General guidance is provided below. Consult the submission guidelines for detailed instructions. **Make sure that all information entered here is included in the Methods section of the manuscript.**	N/A

**Format for specific study types**<br><br>**Human Subject Research (involving human participants and/or tissue)**<br>• Give the name of the institutional review board or ethics committee that approved the study<br>• Include the approval number and/or a statement indicating approval of this research<br>• Indicate the form of consent obtained (written/oral) or the reason that consent was not obtained (e.g. the data were analyzed anonymously)<br><br>**Animal Research (involving vertebrate animals, embryos or tissues)**<br>• Provide the name of the Institutional Animal Care and Use Committee (IACUC) or other relevant ethics board that reviewed the study protocol, and indicate whether they approved this research or granted a formal waiver of ethical approval<br>• Include an approval number if one was obtained<br>• If the study involved *non-human primates*, add *additional details* about animal welfare and steps taken to ameliorate suffering<br>• If anesthesia, euthanasia, or any kind of animal sacrifice is part of the study, include briefly which substances and/or methods were applied<br><br>**Field Research**<br><br>Include the following details if this study involves the collection of plant, animal, or other materials from a natural setting:<br>• Field permit number<br>• Name of the institution or relevant body that granted permission	
**Data Availability**<br><br>Authors are required to make all data underlying the findings described fully available, without restriction, and from the time of publication. PLOS allows rare exceptions to address legal and ethical concerns. See the PLOS Data Policy and FAQ for detailed information.	Yes - all data are fully available without restriction

Powered by Editorial Manager® and ProduXion Manager® from Aries Systems Corporation

A Data Availability Statement describing where the data can be found is required at submission. Your answers to this question constitute the Data Availability Statement and **will be published in the article**, if accepted.<br><br>**Important:** Stating 'data available on request from the author' is not sufficient. If your data are only available upon request, select 'No' for the first question and explain your exceptional situation in the text box.<br><br>Do the authors confirm that all data underlying the findings described in their manuscript are fully available without restriction?	
**Describe where the data may be found in full sentences. If you are copying our sample text, replace any instances of XXX with the appropriate details.**<br><br>• If the data are **held or will be held in a public repository**, include URLs, accession numbers or DOIs. If this information will only be available after acceptance, indicate this by ticking the box below. For example: *All XXX files are available from the XXX database (accession number(s) XXX, XXX.)*.<br>• If the data are all contained **within the manuscript and/or Supporting Information files**, enter the following: *All relevant data are within the manuscript and its Supporting Information files.*<br>• If neither of these applies but you are able to provide **details of access elsewhere**, with or without limitations, please do so. For example:<br><br>*Data cannot be shared publicly because of [XXX]. Data are available from the XXX Institutional Data Access / Ethics Committee (contact via XXX) for researchers who meet the criteria for access to confidential data.*<br><br>*The data underlying the results presented in the study are available from (include the name of the third party*	The data is all provided in the SI files<br>SM1 dataset and previous datasets<br>SM2 Additional statistical tables and information<br>SM3 Rcode

Powered by Editorial Manager® and ProduXion Manager® from Aries Systems Corporation

and contact information or URL).<br>• This text is appropriate if the data are owned by a third party and authors do not have permission to share the data.<br><br>* typeset	
Additional data availability information:	Tick here if your circumstances are not covered by the questions above and you need the journal's help to make your data available.

Manuscript

Click here to access/download;Manuscript;Manuscript final limpio.docx

Particle size distribution: an experimental study using southern African reduction methods and raw materials

de la Peña, P.[1,2,3¶*], Thomas, M.[4¶]; Molefyane, T.R.[5]

1 Departamento de Prehistoria y Arqueología. Universidad de Granada, Spain.

2 McDonald Institute for Archaeological Research, University of Cambridge, United Kingdom

3 Evolutionary Studies Institute, University of the Witwatersrand, South Africa.

4 UMR 5608 TRACES, Université Toulouse Jean Jaurès, Toulouse, France

5 Guías de Espeleología y Montaña (GEM), Spain

E-mail: paloma.delapenya@gmail.com (PdlP)

Abstract

We experimentally created a particle size dataset that is based on reduction sequences and raw materials typical of the Middle and Later Stone Age in southern Africa. The reason for creating this new dataset is that current particle size frameworks are based, almost exclusively, on flint and western European knapping methods. We produced the dataset using knapping methods and raw materials frequently encountered in the southern African archaeological record because we wanted to test whether it has the same distribution as particle size datasets experimentally created in Europe, and to initialise the production of a database for use in the analysis of lithic assemblages from southern African Late Pleistocene deposits. We reduced 117 cores of quartz, quartzite, jasper, chalcedony, hornfels, and rhyolite. The knapping methods selected were unidirectional, discoidal*, Levallois* recurrent and bipolar flaking. In this article we compare this new particle size distribution dataset with the results obtained from previous experiments. We found that the southern African dataset shows a wider size range distribution, which seems to be explained by differences in knapping methods and raw materials. Our results show that there is overlap between the distribution of the southern African experimental knapping dataset and the sorting experiment conducted by Lenoble on flint artefacts in a runoff context. This article shows that a particle size analysis is not sufficient on its own to assess the perturbation of an archaeological assemblage and must be coupled with other analytical tools.

Introduction

Stone Age sites are complex sedimentary accumulations, the formation of which depends on a combination of anthropogenic and natural processes [1–4]. Natural processes, including sedimentary processes, have often altered the spatial organization, composition, and chronological signal of the archaeological record [5]. To measure this loss of information on sites, disturbance assessment studies need to be carried out. Any archaeological remains can undergo the effect of sedimentary processes,

governed by physical laws [4,6]. Thus, particle shape, size and density are the main physical criteria used to measure the degree of perturbation of the archaeological record [2]. The particle size analysis of lithic artefacts was first applied in Africa by K. Schick [2]. She pointed out that in many cases the sites are associated with sedimentary contexts affected by water (alluvial environments, lake margins, basins, marine beaches or even spring deposits). In her seminal work, K. Schick [2] conducted experiments to understand the spatial dispersion and size-class distribution (maximum length of artefacts considered) of lithic industries affected by water flow. She concluded that water flow can substantially, if not radically, affect archaeological assemblages [7,8]. In addition, other natural processes are likely to have an impact on the particle size distribution of lithic assemblages, such as solifluction [9,10], trampling [11–18], and aeolian processes [19].

Other studies have extended and followed a similar methodology to Schick's work, applying it mainly to European Palaeolithic contexts, particularly in the southwest of France [4,20,21]. In order for there to be a systematic approach to the study of sites, A. Lenoble and P. Bertran developed a method based on sieving instead of manually measuring all lithic artefacts [4,20,21]. Based on the observation that rock knapping consistently caused the same particle size distribution, Lenoble and Bertran and colleagues [4,20,21] proposed to compare an experimental knapping assemblage (from [21] called "EU database" for ease of reading in this article) to an experimental one subjected to overland water flow (from [4] called "RUNOFF dataset" for ease of reading in this article) (see supplementary material 1(SM1)). The particle size distribution of these two sets of experiments: "EU dataset" and the "RUNOFF dataset" show separated distributions in a ternary plot (Fig 1). The knapping experiments were done mainly on flint and quartzite and included blade production, *Levallois* and discoid flaking, and bifacial shaping. The authors also added microlithic reduction sequences inspired by Palaeo-Eskimo knapping methods from the eastern Canadian Artic (called "ESKI dataset" for ease of reading in this article, from [22]).

In all knapping experiments, the distribution sizes follow a Weibull pattern, being long-tailed and positively skewed [23] with a proportion of largest pieces tending towards 0 [2,4,20–23], whereas the results of the "RUNOFF dataset" highlight different kinds of distributions according to where one stands

in the runoff system. This clear distinction between both experiments in the final model allows one to test the size sorting of lithic archaeological assemblages ([20,21], Figure 1). However, this experimental framework mainly includes debitage methods and raw materials typical of European contexts. Thus, the use of both datasets to study African assemblages is questionable, as different raw materials and knapping methods were used in Africa in the past.

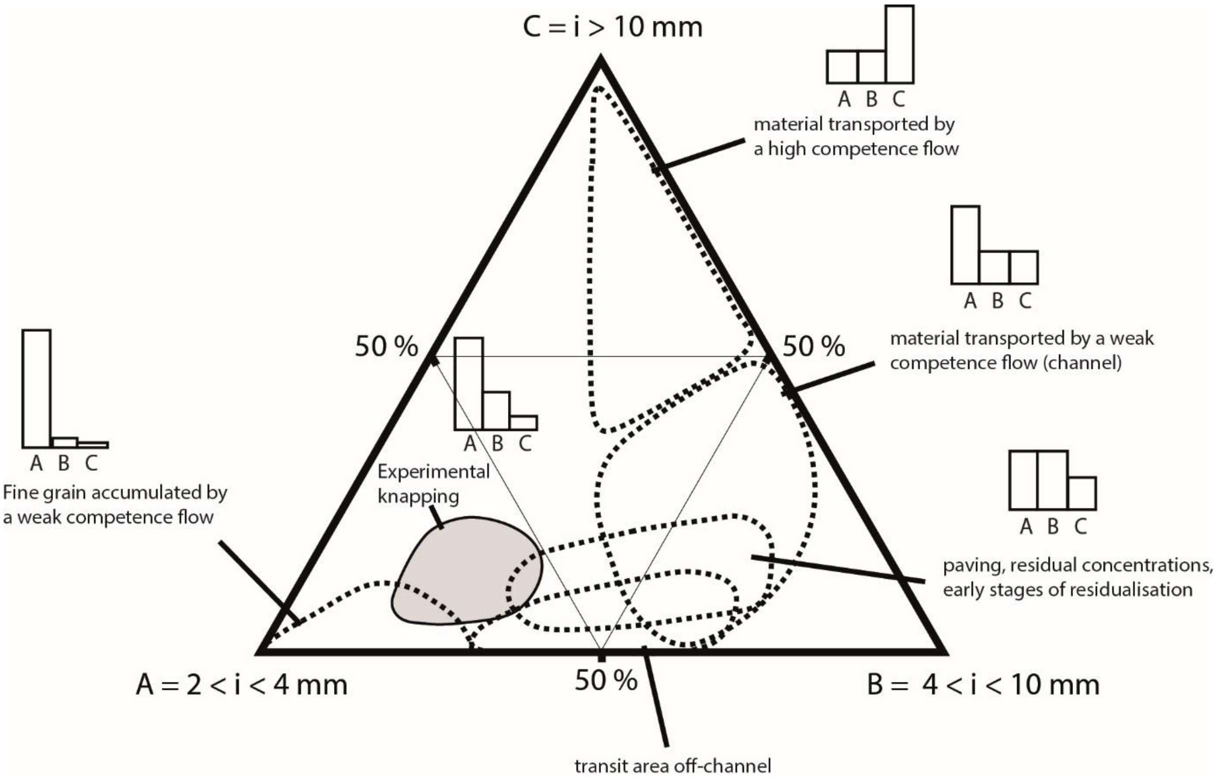

Fig 1: Particle size evolution of a lithic assemblage affected by overland water flow from the experiment of Lenoble [4]. The light brown area is the distribution of the EU dataset [20,21]. The dashed areas correspond to the size class distribution in the RUNOFF experiment [4]. The "A" triangle summit corresponds to the [2;4] mm width size class percentage, the "B" triangle summit to the [4;10] mm width size class percentage and "C" to the [10;+∞ mm width size class percentage]. i = ?. From [4].

Questions and objective

In this paper, we contribute experimental research to the field of lithic archaeology, focusing strictly on southern African knapping methods and raw materials. The reason for creating this specific experimental dataset was to assess if the previous experimental datasets created in Europe for Middle and Upper Palaeolithic archaeological contexts (such as EU and RUNOFF datasets [4,20,21]), and mainly based on flint (and to a lesser extend quartzite) [4,20,21], were useful or not for the understanding of

southern African archaeological lithic assemblages. We wanted to test if similar patterns of particle size distribution would be reproduced in southern African assemblages. The reason for this specific enquiry is that in southern African late Pleistocene contexts there are different methods of debitage and raw materials from those used in Europe. We hypothesized that different knapping methods and raw materials would create different proportions of lithic particles. For this purpose, we performed a large experiment on particle size analysis focusing on southern African rock varieties (automorphic and xenomorphic quartz, quartzite, jasper, chalcedony, hornfels and rhyolite) and knapping methods typical of the Middle and Later Stone Age records (unidirectional, bipolar knapping, discoid, recurrent *Levallois*). The knapping methods selected are relatively common in Middle and Later Stone Age assemblages (*vid.* [24,25] *inter alia*). The raw materials are the most common rocks used for knapping in the interior parts of southern Africa for the Late Pleistocene. The exceptions to this selection are dolerite and silcrete, which were excluded from this analysis because dolerite is an extremely difficult rock to knap and silcrete does not occur in any of the sites that we currently study.

This new experimental dataset (called "SA dataset" in this article, see SM1) will be useful for our own research on Middle and Later Stone Age sites and offer a feely available dataset for other archaeologists working on African material. Additionally, this new investigation serves as a control experiment that is comparable to previous European publications on this type of research [20,21]. Moreover, this new dataset will test if different raw materials and knapping methods, such as bipolar knapping and unidirectional reduction, generate different results from the previous experiments [20,21]. The new results and the comparison with previous experiments will ultimately serve to study in depth and without bias the archaeological assemblages from southern Africa. Our immediate goal is to compare the results of this experimental research with archaeological lithic assemblages from sites such as Mwulu's Cave, Border Cave and Marshill (studied by one of the authors, PdlP). Our research questions are:

(1) What is the particle size distribution specific to southern African raw materials and knapping methods?

(2) Are there statistically significant differences regarding raw materials?

(3) Are there statistically significant differences regarding the knapping methods?

(4) Is the particle size distribution of the SA dataset different from the EU dataset?

(5) Is the particle size distribution of the SA dataset different from the RUNOFF dataset?

The null hypotheses are that there is no statistical difference between the different raw material and knapping method datasets compared in this study, and compared with previous published datasets [4,20,21].

Material and methods

One of the authors (PdlP) conducted all of the experimental knapping, having several years of stone tool making experience. The experiment includes the most common southern Africa rocks and lithic reductions used during the Middle and the Later Stone Age (*vid. supra)*. They are summarized in Table 1 with the number of cores reduced for each one of the knapping methods and raw material. In Fig 2, we show schematically all the knapping methods deployed in this analysis. In total 117 cores were knapped for this experiment (see SM1). In Fig 3 the main raw materials are illustrated, alongside a selection of tools made during the experiments.

Table 1: Synthesis of the knapping methods and raw materials used in this experiment (A-quartz = Automorphic quartz, X-quartz = Xenomorphic quartz)

	Chalcedony	A-quartz	Hornfels	Jasper	Quartzite	Rhyolite	X-quartz	Total
Bipolar	8	4		10			10	**32**
Discoidal			10		10	10	12	**42**
Levallois	4		16					**20**
Unidirectional	10		8	5				**23**
Total	**22**	**4**	**34**	**15**	**10**	**10**	**22**	**117**

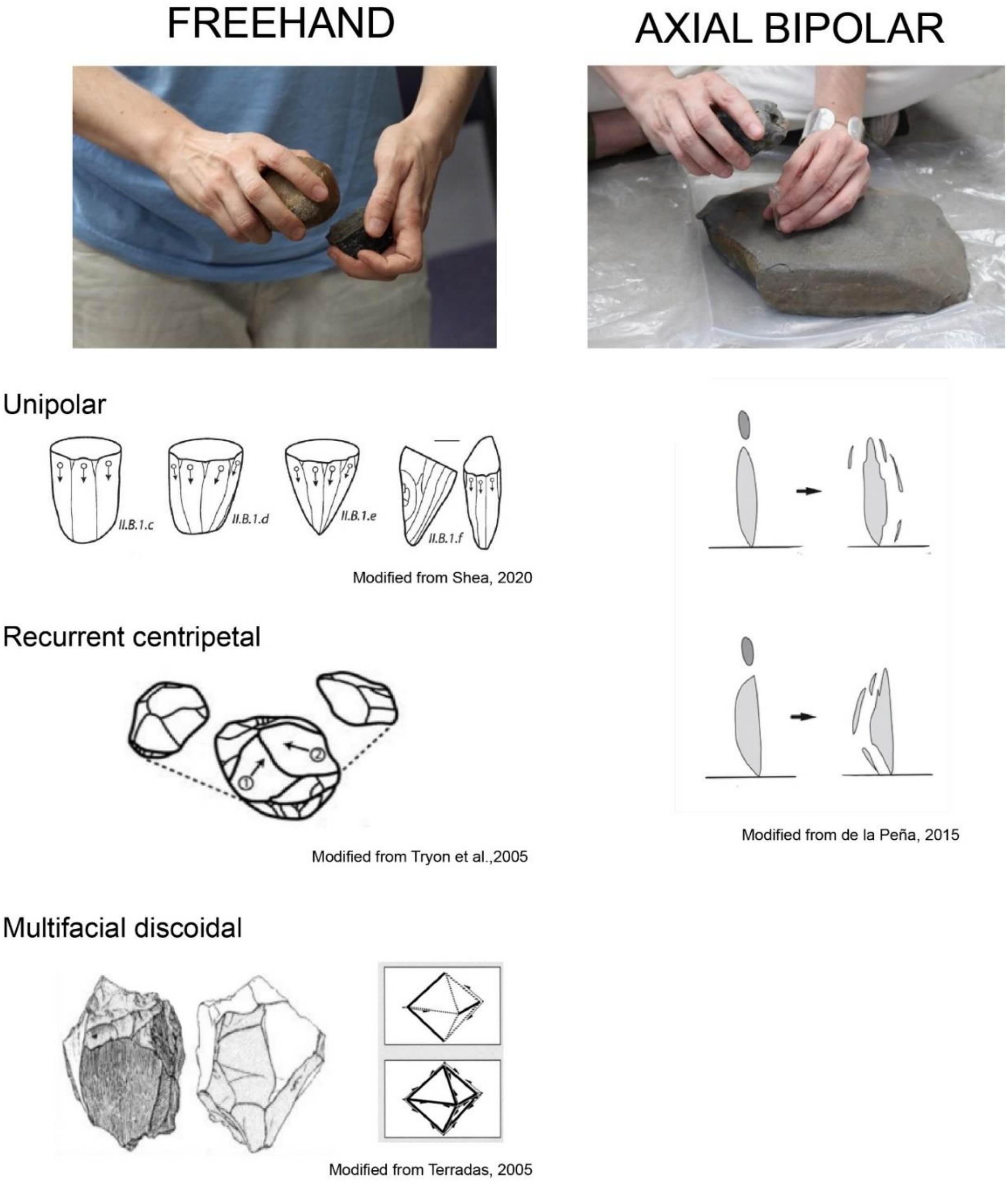

Fig 2: Examples of Middle and Later Stone Age reduction sequences employed in this knapping experiment: discoidal, *Levallois* recurrent, unidirectional, and axial bipolar . The figures are modified from [15, 40, 48, 53]. On the left freehand variants and on the right bipolar axial variant towards miniaturization. Photos: Paloma de la Peña

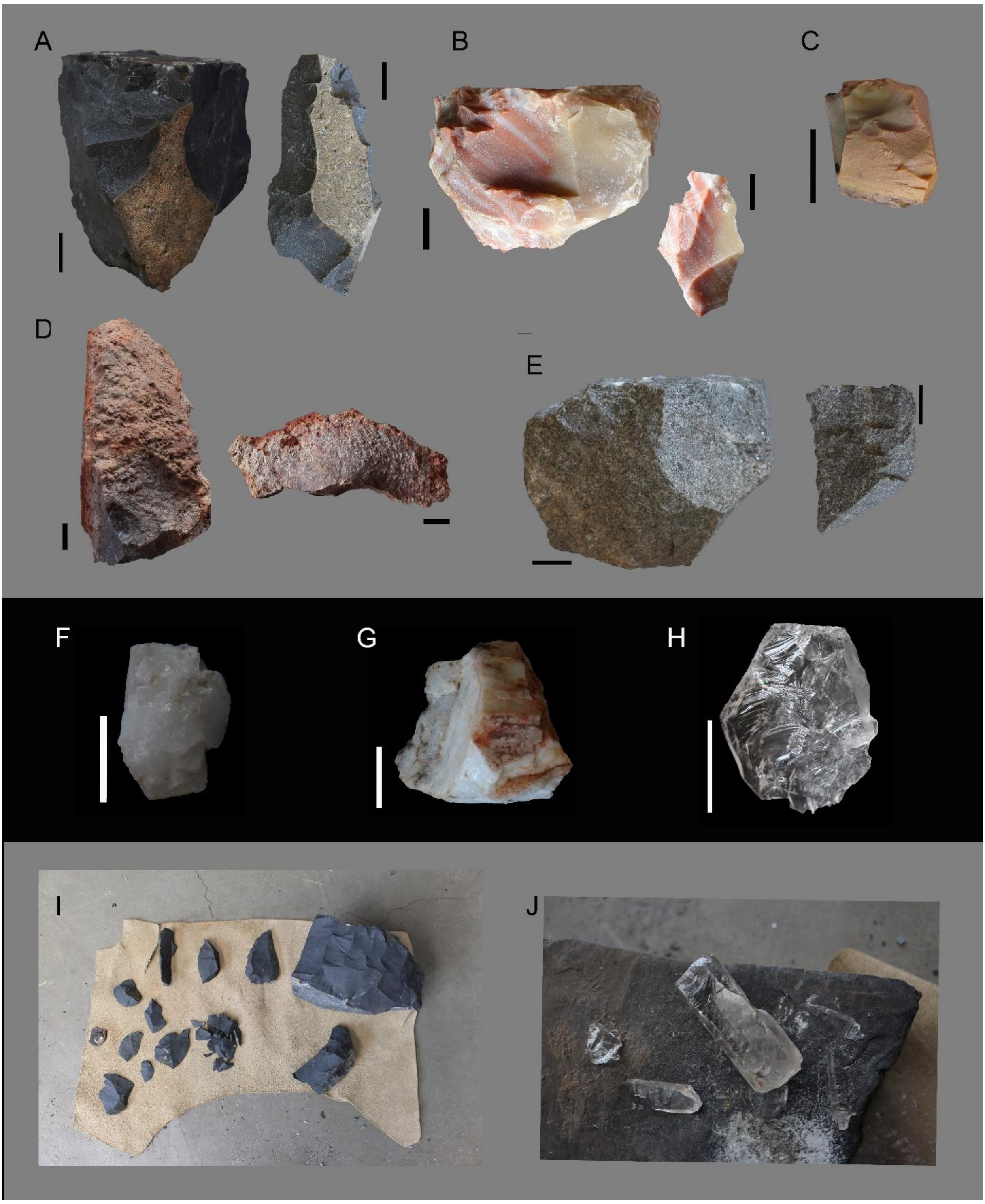

Fig 3: Raw materials used for this experiment. A. Hornfels (unidirectional core and flake). B. Jasper (unidirectional core and flake). C. Chalcedony (chunk). D. Rhyolite (core fragment) and flake (ventral view). E. Quartzite (discoidal core and flake, dorsal view). F. Xenomorphic quartz (bipolar core). G. Xenomorphic quartz (core fragment). H. Automorphic quartz (bipolar core). I. Selection of blanks and cores in hornfels during one of the unidirectional reduction sequences. J. Core and blanks in the middle of the reduction through bipolar axial knapping of one of the automorphic quartz cores. All scales are 1 cm except for H, which is 5 mm. All the material presented is from this experiment, except for C and H, which were previously produced. Photos: Paloma de la Peña.

During the experiments, we tried to reduce the core until exhaustion, in other words, until no more flakes could be extracted. For the knapping technique in this experiment, we used a hard mineral percussion with a banded iron pebble from KwaZulu-Natal and a soft mineral hammer, a sandstone pebble from the Eastern Cape.

The specific knapping methods selected are as follows:

- Bipolar knapping: A method in which the core is placed on an anvil and held with the bare hand. The rock is struck from above with a hammer held in the other hand, causing blanks to fly off from the top and from the edge that is in direct contact with the anvil [26,27]. Bipolar knapping has been reported in the Middle Stone Age and Later Stone Age [25].
- Discoidal Knapping: In this experiment we used what [28] named multifacial discoidal knapping (called "discoidal knapping"), which is equivalent to "centripetal knapping" according to [29]. In this sub-variant the knapper searches for 45 degree angles to obtain flakes until the core is exhausted, changing the striking platform almost every time and using the removals as such.
- Unidirectional knapping: In this method, we tried to exploit as much as possible from one striking platform following a unidirectional sequence of removals. Once the knapping surface from the first striking platform was exhausted, we rotated the core and continued knapping from a different striking platform, following the same strategy if possible.
- *Levallois* recurrent (also referred to in the African literature as 'prepared cores'): In our experiments we followed the guidelines explained by E. Boëda [30]. Sometimes during reduction, we could extract two series of *Levallois* flakes. In some cases, we finished the knapping of these cores using discoidal reduction. This was done to obtain the most out of the volume of the rock in the final stage, and only when there was no possibility to prepare two hierarchical knapping surfaces in a *Levallois* fashion.

The raw materials used for the experiments (Fig 3) include:

- Hornfels, a fine- to medium-grained metamorphic rock that is formed by contact metamorphism on the margins of igneous intrusions that crosscut the country rock [31]. The two varieties of hornfels used for this study come from two different dolerite dykes that intruded shales, one in the Eastern Cape and another in the Northern Cape. The variety used from the Eastern Cape was obtained from a river cutting of a massive hornfels contact metamorphism layer. Large chunks of the material were extracted and broken into smaller blocks for the experiments. The hornfels from the Northern Cape came in small slabs, usually of less than 10 cm for their longest axis.
- Chalcedony, a cryptocrystalline quartz [31]. The one we used is not banded, and in South Africa it mostly occurs in white, gray, black, or blue shades. Chalcedonies fill cavities and line fissures in rocks. In the Drakensberg and Lebombo Group, they occur in both basalts and rhyolites. Sometimes chalcedony contains a monoclinic polymorph called moganite [31]. The chalcedony of this experiment comes entirely from Siberia Farm, near a rock art site also known in the area as Franshoek. It is an outcrop with varieties of rocks ranging in colour from green to orange.
- Jasper, a chert coloured by the presence of iron [31]. The variety used for this experiment was banded red jasper from the Eastern Cape.
- Automorphic quartz (crystal quartz, called A-quartz in this article, following [32]), a euhedral mineral with the shape of a single large hexagonal crystal terminating alternately in major and minor rhombohedral faces [33]. Its flat faces are well developed around a symmetrical axis and bounded by rectilinear ridges [34,35]. The crystal quartz used for this experiment comes from a small vein in Limpopo Province, from the surroundings of Mwulu's Cave [36], specifically from the Black Reef Formation.
- Xenomorphic quartz (following [32], called X-quartz in this article), a solid polycrystalline aggregate with an anhedral texture formed by a close-packed mass of crystals that are not usually bounded by their own crystal faces, but have their outlines pressed on them by the

adjacent crystals [35,37]. All the xenomorphic quartz used for this experiment comes from the Cradle of Humankind (Gauteng) area, near the site of Swartkrans.

- Quartzite, a metamorphosed sandstone. All the Quartzite used in this experiment comes from the Black Reef Formation in the surroundings of Mwulu's Cave [36] in Limpopo Province.
- Rhyolite, an extrusive igneous rock with a high silica content. Rhyolite is made up of quartz, plagioclase, and sanidine, with minor amounts of hornblende and biotite. Trapped gases often produce vugs in the rock. These often contain crystals, opal, or glassy material. The rhyolite used for this experiment comes from the immediate surroundings of Border Cave in the Lebombo mountains (KwaZulu-Natal, South Africa).

To enable comparison of the southern African experimental knapping dataset with the RUNOFF dataset [4] and the EU dataset [20,21] we have used the same size classes. The previous experiment used mesh of 2, 4, 5, 10, 20, 31.5 and 50 mm. After gathering all the particles produced during the knapping process, we hand-sieved the material in the same way recommended by Bertran et al. [20,21]. As particle size analysis is time consuming, to speed up the counting for material-rich assemblages, the weights of small particles (2 and 4 mm) were obtained using mass/count ratios. We thus counted all the particles per size class and weighed them.

To describe our dataset and compare it with previous data we used ternary plots made with Past3. [38]. Additional statistics were made using the method developed by Weaver et al. [39] to obtain 95% confidence intervals in ternary plots (Fig 4). To compare the different datasets, we use Principal Component Analysis (PCA) performed under R using the FactoMineR package [40]. The MANOVA tests were run using Past3. The statistical descriptions of the PCA analysis are available in SM2 and SM3.
Raw material and reduction strategies choices were made to follow as closely as possible the southern African archaeological context (*vid. supra*). All knapping methods are not applied to all raw materials, as for example there are certain knapping methods that never appear on certain raw materials in the southern African archaeological record. For instance, to the best of our knowledge, there is no *Levallois*

reduction on automorphic or xenomorphic quartz in archaeological assemblages, and in the same vein there is no record of bipolar knapping on anvil for hornfels The number of experiments for each one of the variants explored per raw material is not consistent, as in some cases during the experiment the bad quality of the raw material (because of flaws and natural fractures) did not allow us to complete a reduction sequence. We tried to have at least five examples for each one of the raw materials and debitage variants and, ideally, to get 10 examples. This was not possible for all the variants selected (Table 1) because of the reasons explained. Thus, we assume statistical bias in the comparisons we performed as each raw material has not been knapped through each knapping method. Thus it is not possible to measure the exact effect of these two last variables on particle size distribution. The aim being to compare archaeologically compatible options.

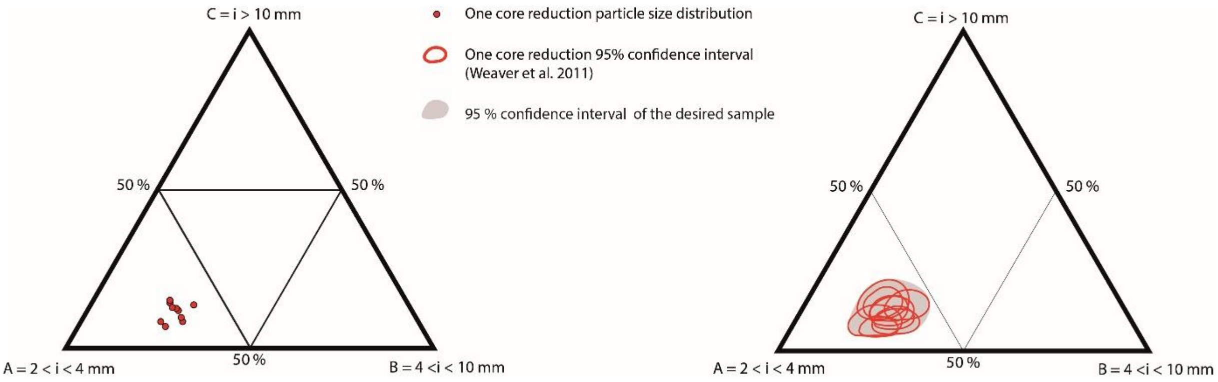

Fig 4: Illustration of how we applied the Weaver et al. (2011) method to create 95% confidence ellipse intervals in the ternary plots.

Results

What is the particle size distribution resulting from southern African raw materials and knapping methods?

In SM1 we present the particle size composition of the SA dataset, describing the size classes and their weights. The histogram of the percentages per size class, considering knapping methods and raw

materials, clearly shows that for all the raw materials and knapping methods there is a gradual decrease in particle numbers, as size classes increase. In all the series, the 5 mm particle class is slightly more abundant than the 4 mm size class (Fig 5). In all of the experiments, whether considering raw material or knapping method, the size classes 2 to 4 mm constitute around 40-60% of the particles (Fig 6).

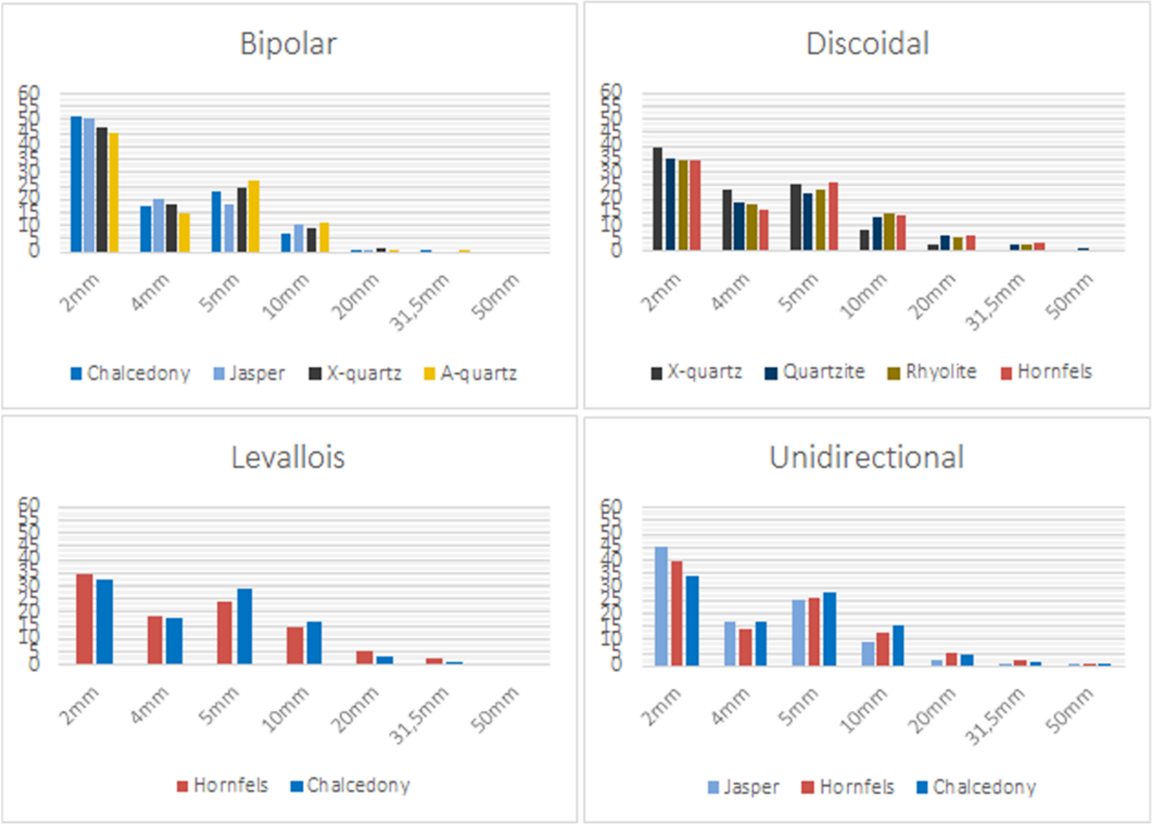

Fig 5: Histograms comparing mean distributions of percentage of particles per size class (counts).

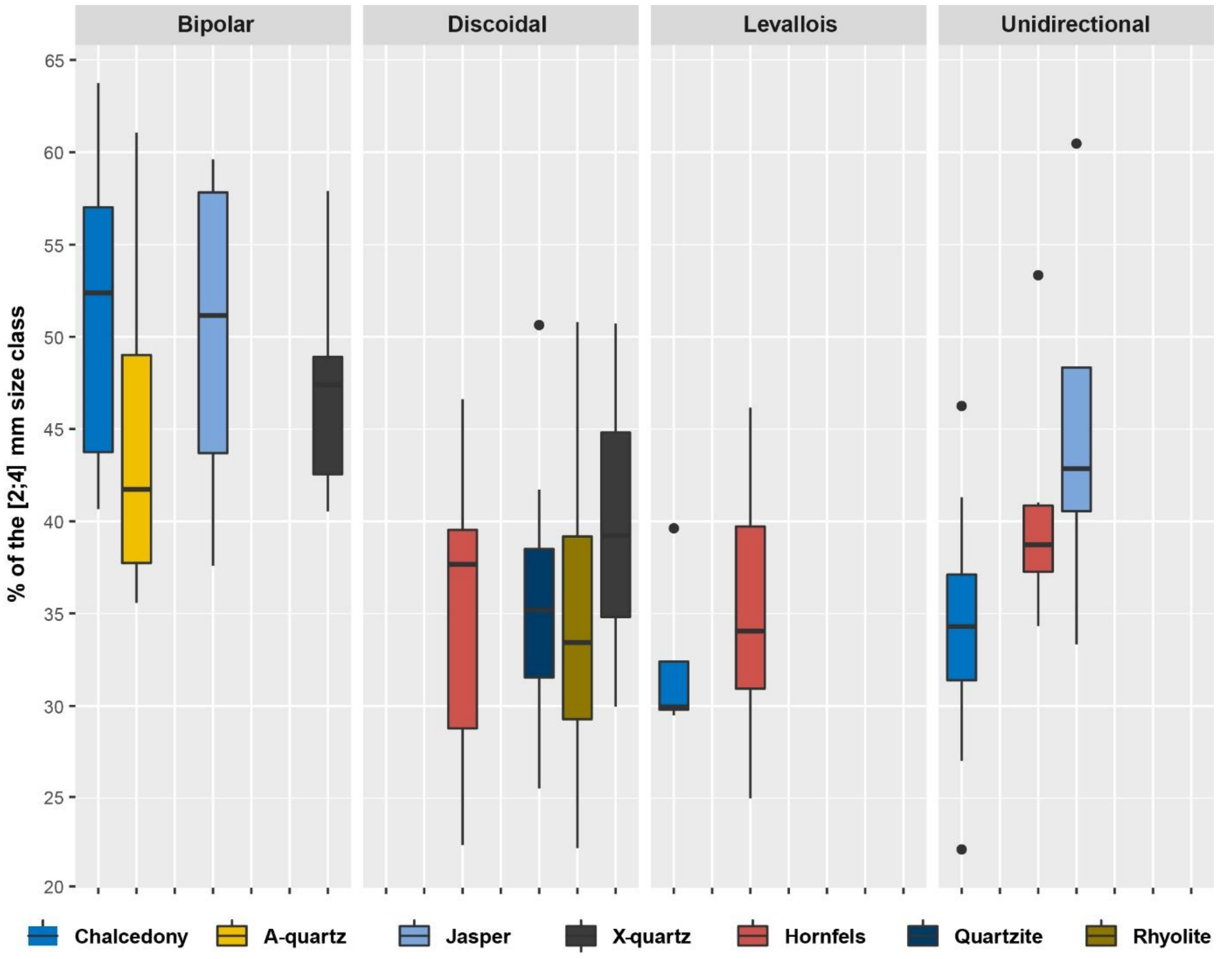

Fig 6: Box plots comparing the proportion of the [2:4] mm size class (counts) depending on the raw material and the knapping method.

The ternary diagrams (Fig 7) show a broad distribution of the SA dataset on experimental debitage size class distribution, predominantly composed of particles between 2 and 4 mm and between 4 and 10 mm. The 95% confidence interval calculated for each debitage group considerably increases the range of distribution for each raw material and knapping method.

In the ternary plots (Fig 8) rhyolite, quartzite and hornfels have a similar distribution towards the centre of the charts. Regarding knapping methods, unidirectional, discoidal, and *Levallois* seem to partially overlap, irrespective of the raw material considered (Fig 9). Furthermore, bipolar knapping produces the smallest fractions (between 2 and 4 mm) irrespective of the raw material. Freehand knapping (unidirectional, discoidal and *Levallois*) and bipolar knapping distribute slightly separately in the ternary plot.

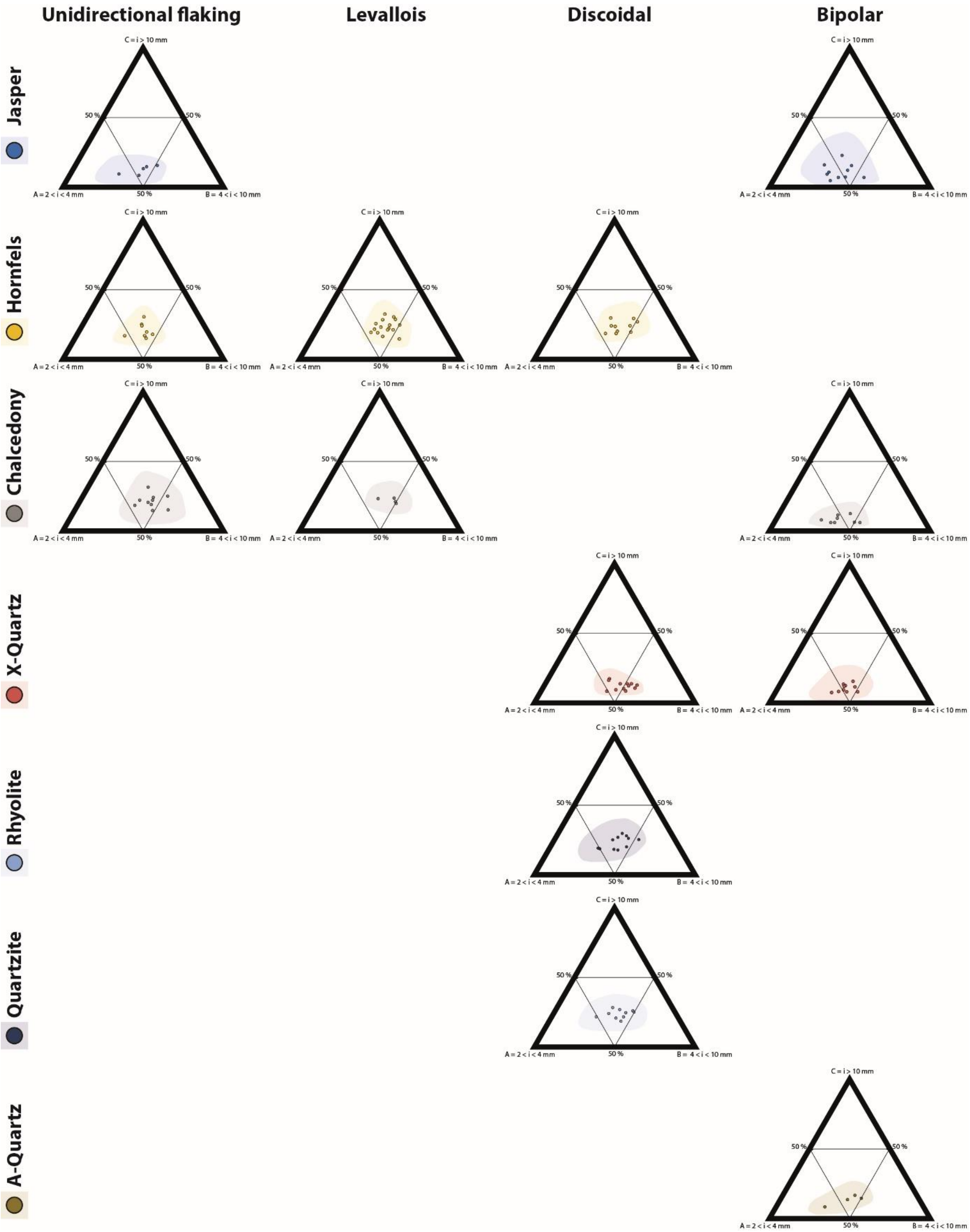

Fig 7: Ternary plots per raw material and knapping method in the SA dataset (counts). Shaded areas are 95% confidence intervals.

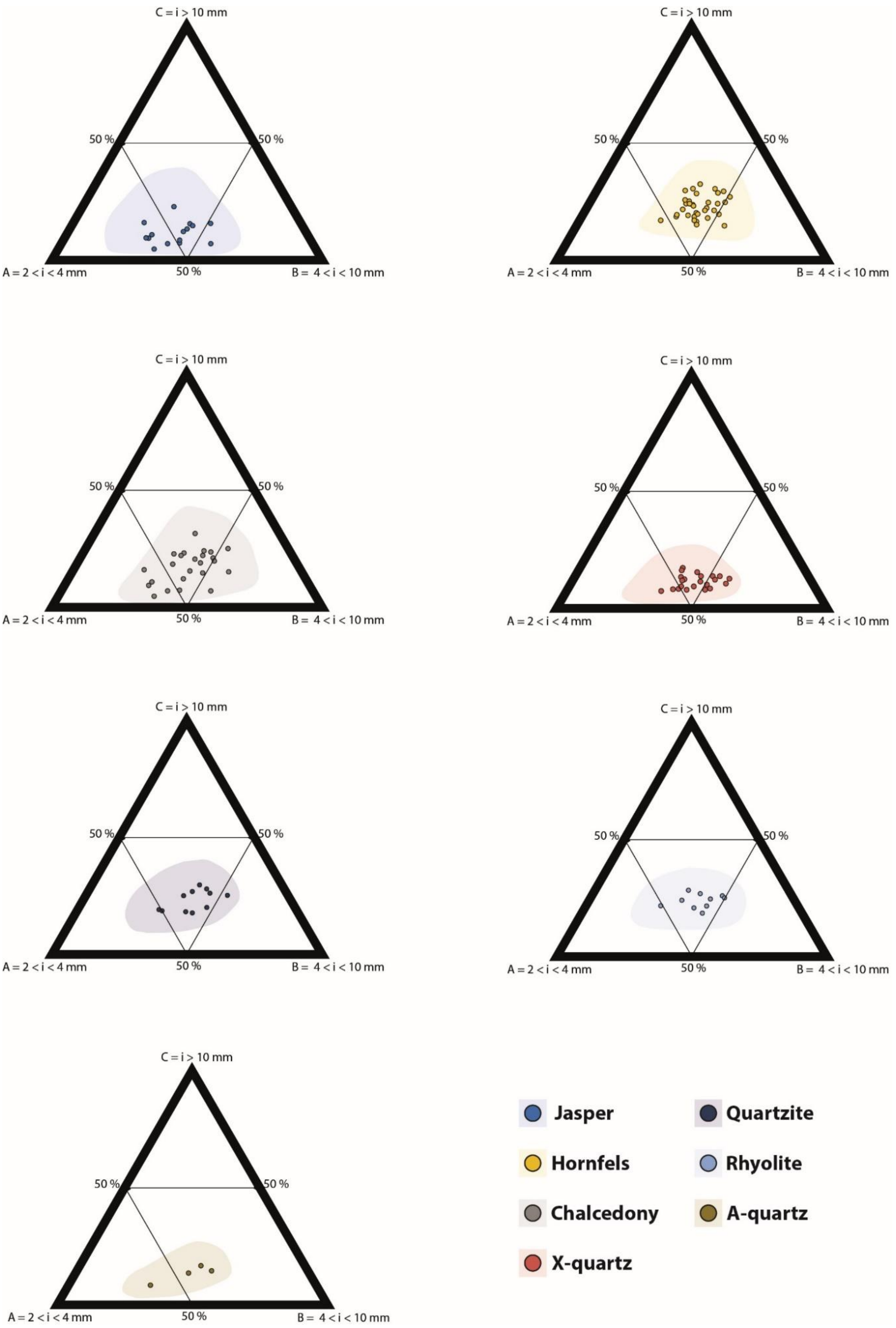

Fig 8: Ternary plots of the particle size distribution per raw material (counts) in the SA dataset. Shaded areas are 95% confidence intervals.

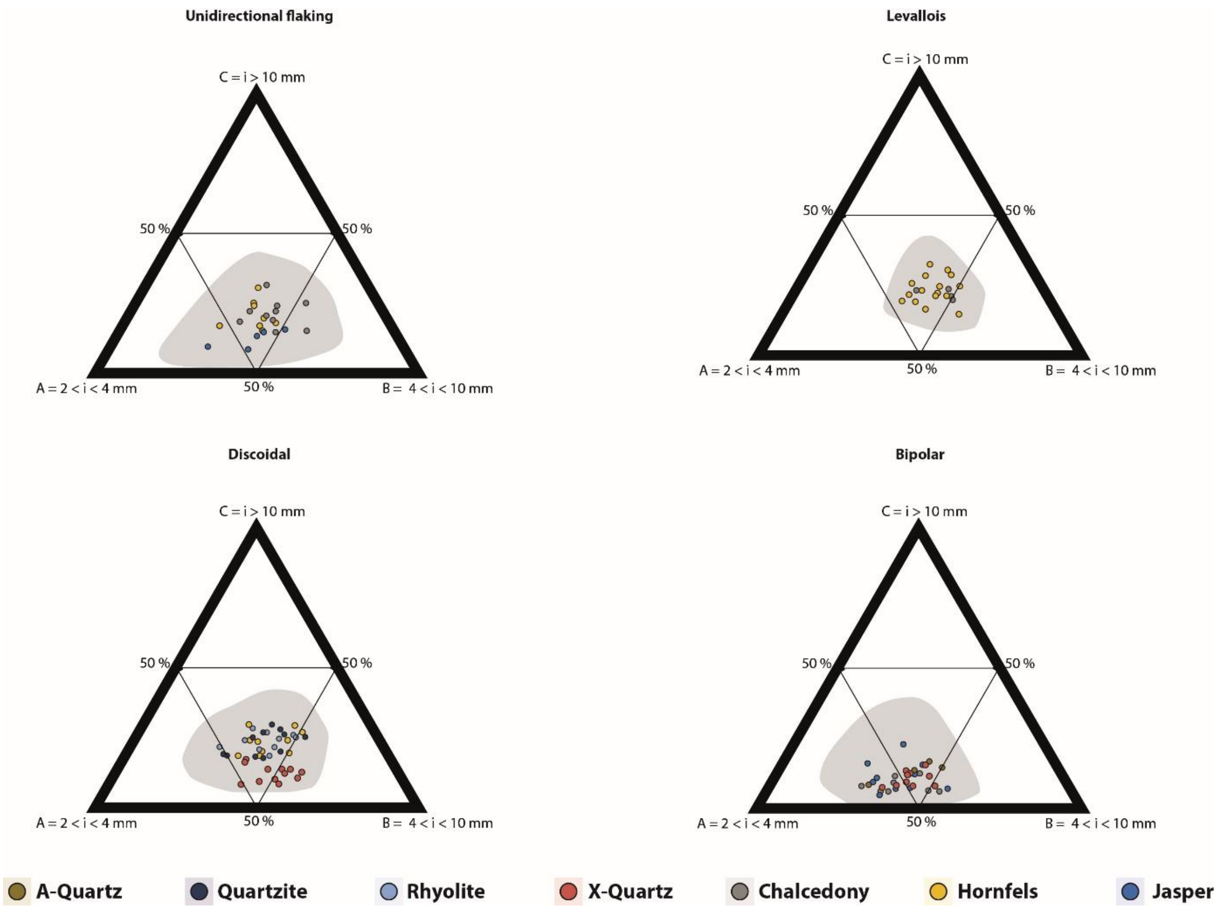

Fig 9: Ternary plots of the particle size distribution per knapping method (counts) in the SA dataset. Shaded areas are 95% confidence intervals.

Are there statistically significant differences between the raw materials?

The factorial space of the "Raw material" principal component analysis (Fig 10 and SM2) shows a clear opposition (mostly first axis, 39%) between, on one hand, chalcedony, jasper, X-quartz and A-quartz, and, on the other hand, hornfels, quartzite and rhyolite. The latter produced larger pieces, but have not been reduced through the bipolar technique. These differences are confirmed by a MANOVA test (using a pairwise comparison) with the raw material as an independent variable and the size distributions as dependent variables (Table 2). A group composed of chalcedony, jasper and x-quartz differs from hornfels, quartzite and rhyolite.

Table 2. Results of the pairwise comparison from the MANOVA test. Raw material is the independent variable, while size classes are the dependent variables.

	Chalcedony	A-Quartz	Jasper	X-quartz	Hornfels	Quartzite	Rhyolite
Chalcedony		18,09	0,16	1,51	*0,00*	*0,00*	*0,02*
A-Quartz	18,09		11,61	12,17	0,14	1,22	3,50
Jasper	0,16	11,61		9,63	*0,00*	*0,00*	*0,01*
X-quartz	1,51	12,17	9,63		*0,00*	*0,00*	*0,00*
Hornfels	*0,00*	0,14	*0,00*	*0,00*		*0,01*	13,83
Quartzite	*0,00*	1,22	*0,00*	*0,00*	*0,01*		9,40
Rhyolite	*0,02*	3,50	*0,01*	*0,00*	13,83	9,40	

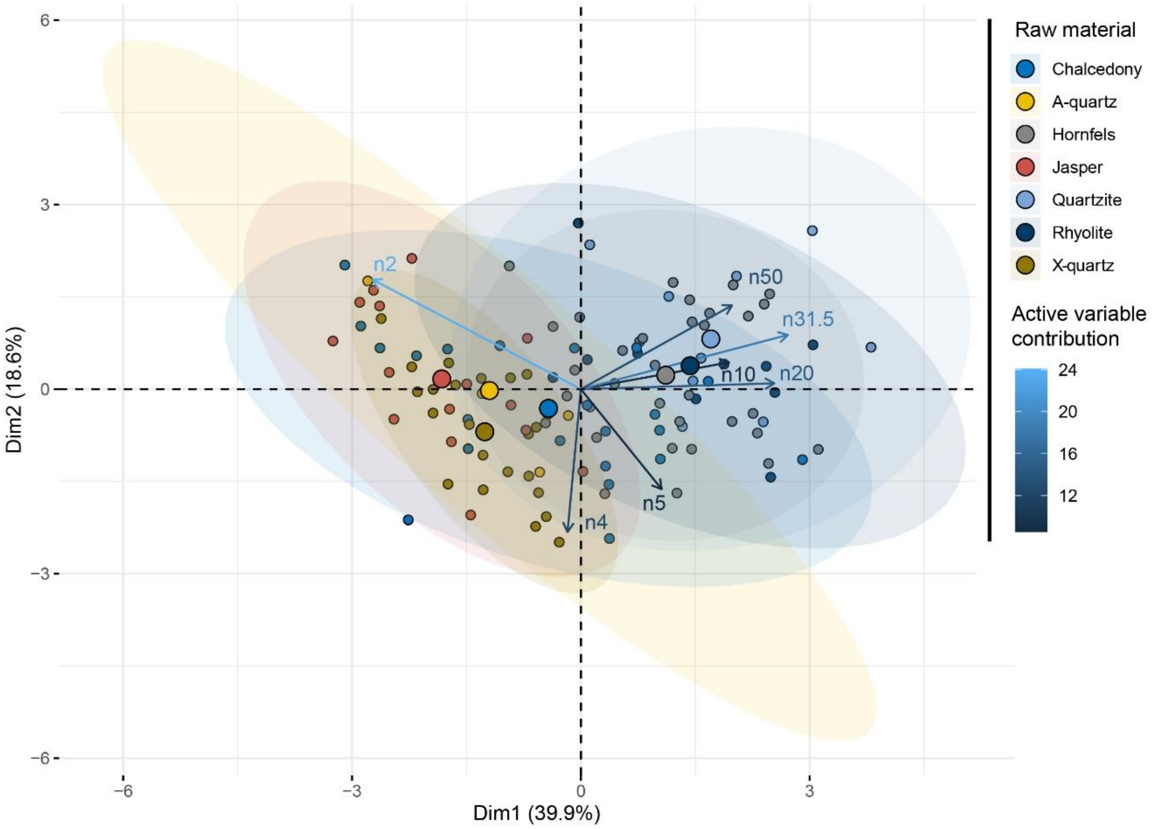

Fig 10: PCA of the SA dataset with the raw material as a supplementary variable.

Are there statistically significant differences between the knapping methods?

The factorial space of the "Knapping method" principal component analysis (Fig 11 and SM2) shows a clear opposition on the first dimension (39% of the variability) between methods producing smaller particles (2 to 4 and 4 to 5 mm width size class) and those producing larger particles (over 10 mm wide). Bipolar knapping is best described by the size range variables 2 to 4 mm and 4 to 5 mm, as opposed to

Levallois, unidirectional and discoidal methods, the distribution of which seem similar and produce larger pieces. These differences are confirmed by a MANOVA test with "Knapping method" as an independent variable (Table 3). Bipolar differs from all the other knapping methods, which have a size distribution that tends to be the same.

Table 3. Results of the pairwise comparison from the MANOVA test. Knapping method is the independent variable, while size classes are the dependent variables.

	bipolar	discoidal	Levallois	unidirectional
bipolar		*0,00*	*0,00*	*0,00*
discoidal	*0,00*		2,48	0,11
Levallois	*0,00*	2,48		2,41
unidirectional	*0,00*	0,11	2,41	

When combining knapping methods and raw material (Fig 12 and SM2), three groups appear in the factorial space. The first one is best described by small particles (2 to 4 mm), and is composed of X-quartz discoidal knapping, all bipolar on anvil, and all jasper knapping experiments. The second group is best described by larger particles. It is composed of all hornfels, quartzite and rhyolite raw materials regardless of the associated knapping method. A third group, composed of chalcedony associated with *Levallois* and unidirectional flaking, is described as intermediate width particles (5 to 10 mm size class).

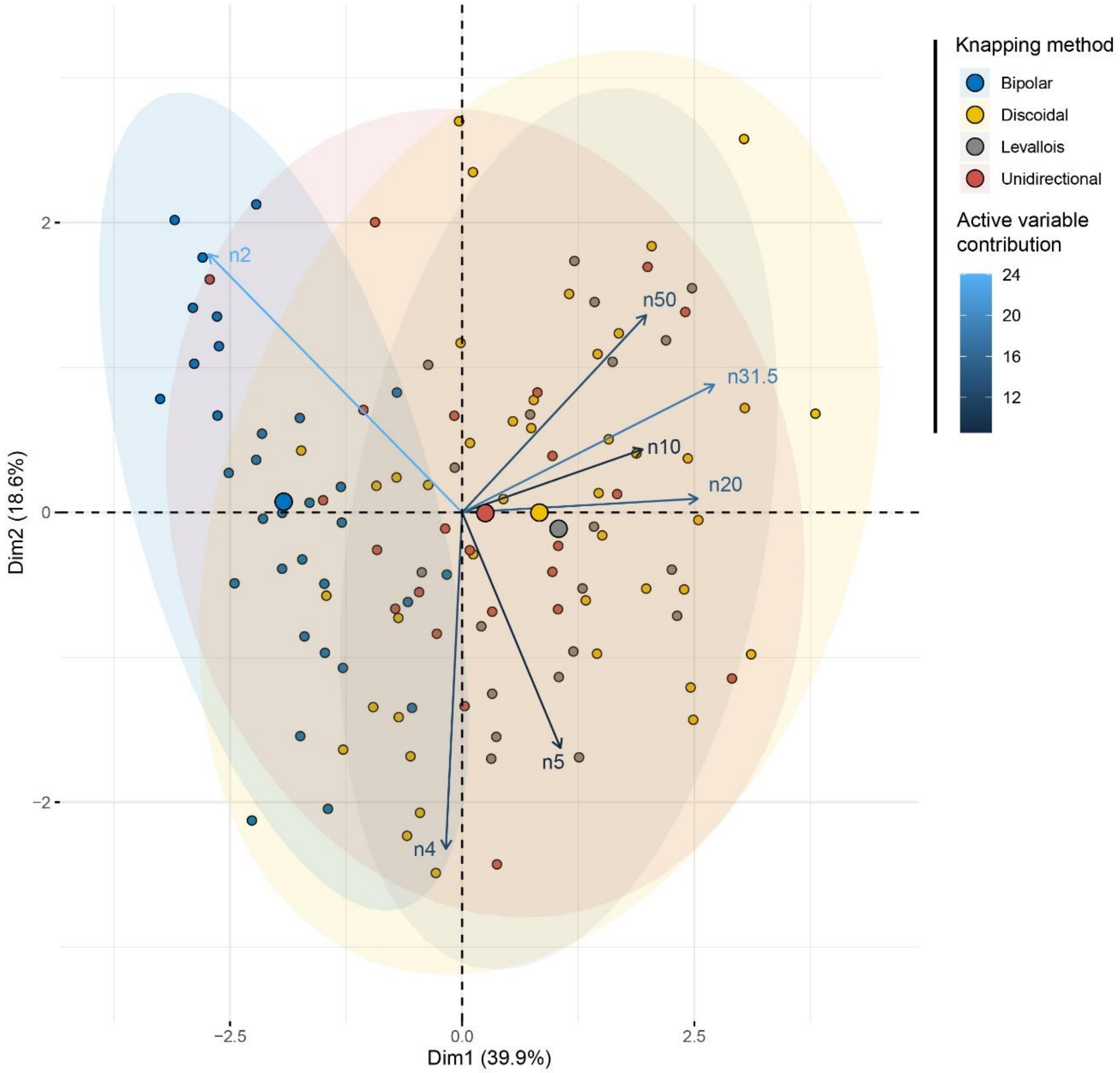

Fig 11: PCA of the SA dataset with the knapping method as a supplementary variable.

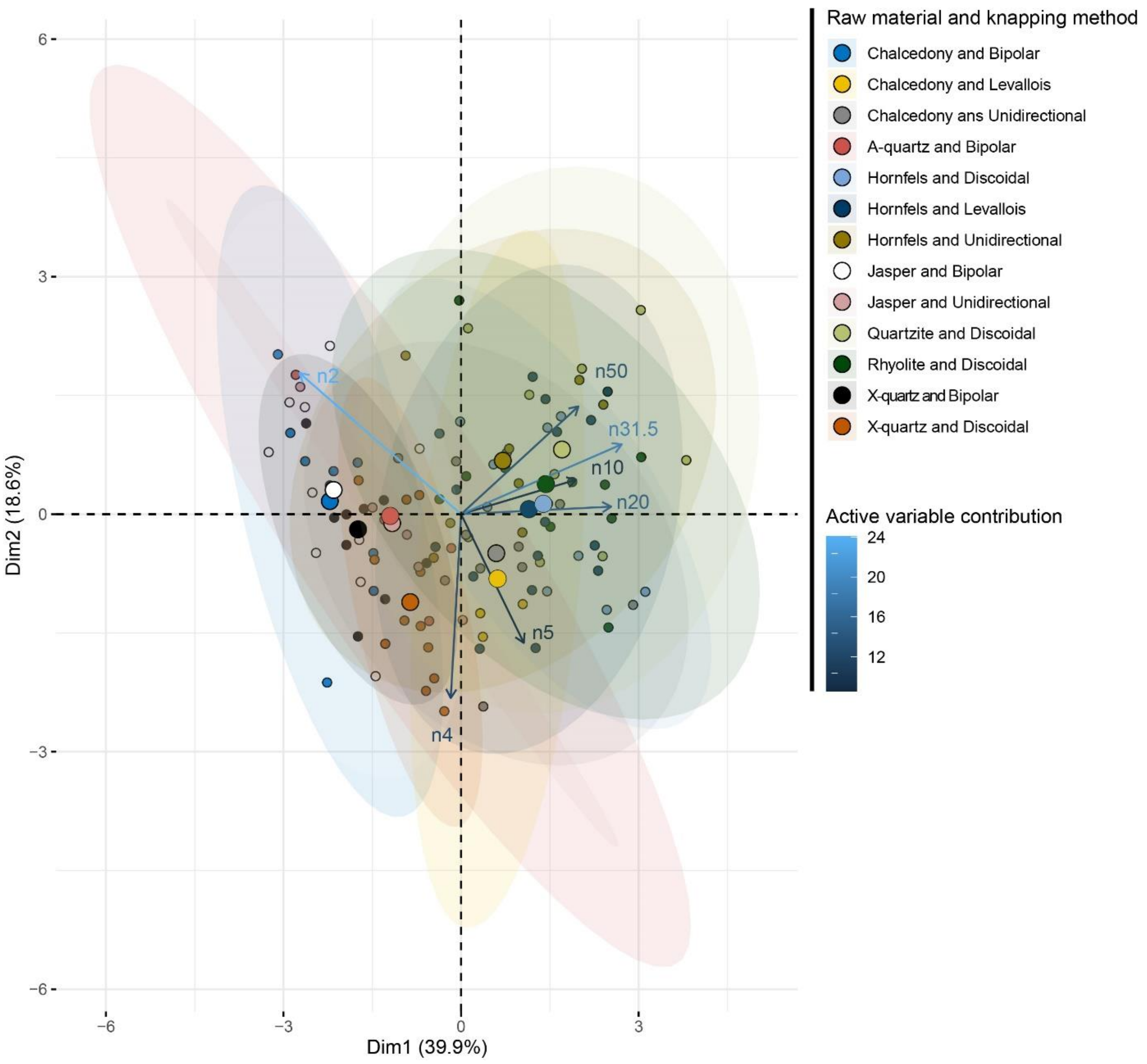

Fig 12: PCA combining knapping and raw material as a supplementary variable (SA dataset).

Is the particle size distribution of the SA dataset different from the EU dataset?

In the ternary diagram we see great differences between the three experimental knapping datasets on particle size distribution (Fig 13). This variability is confirmed by the principal component analysis including the EU dataset on particle size distribution [20,21] and the SA dataset (Figs 14 and 15). When considering the knapping methods (Fig 14 and SM2), three main groups appear in the factorial space. The first one is correlated with small particles (i.e., 2 to 4 mm) and is composed of bifacial shaping and

laminar knapping systems. A second group, best described as intermediate particle sizes, is composed of bipolar blank production. The third group is best described as having larger particle sizes and is composed of the flaking systems (discoidal, *Levallois* and unidirectional).

When considering raw materials as a supplementary variable (Fig 15 and SM2), flint is opposed in the factorial space to A-quartz, X-quartz, jasper, and chalcedony, which are all opposed to a group composed of rhyolite, quartzite and hornfels. These three clusters are best described respectively by their small particle sizes (2 to 4 mm), intermediate sizes (4 to 10 mm) and larger sizes (20 to 50 mm).

When adding the ESKI dataset in the principal component analysis with the data divided according to type of reduction, such as “knapping”, “shaping” and “retouching” (Fig 16 and SM2), we see a clear opposition in the factorial space between “retouching”, producing smaller remains, and “knapping” producing larger remains. “Shaping” falls between the two.

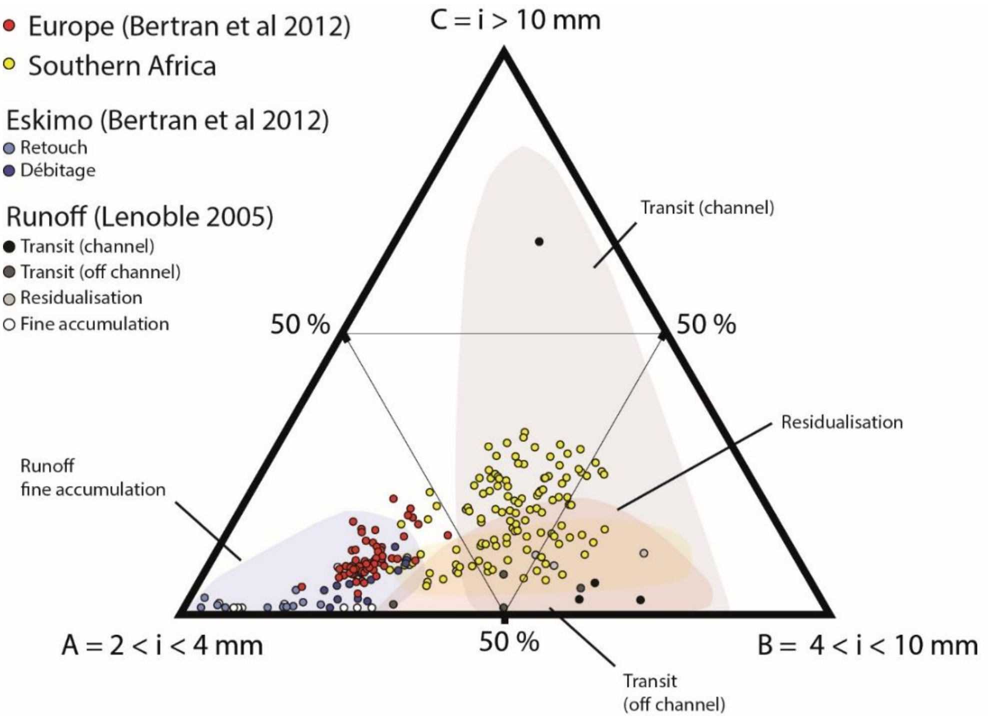

Fig 13: Ternary plots comparing the SA dataset presented in this paper, the EU and the ESKI datasets published in Bertran et al. [20,21] **and the RUNOFF dataset** [4]. The ternary plots contain 95% confidence intervals.

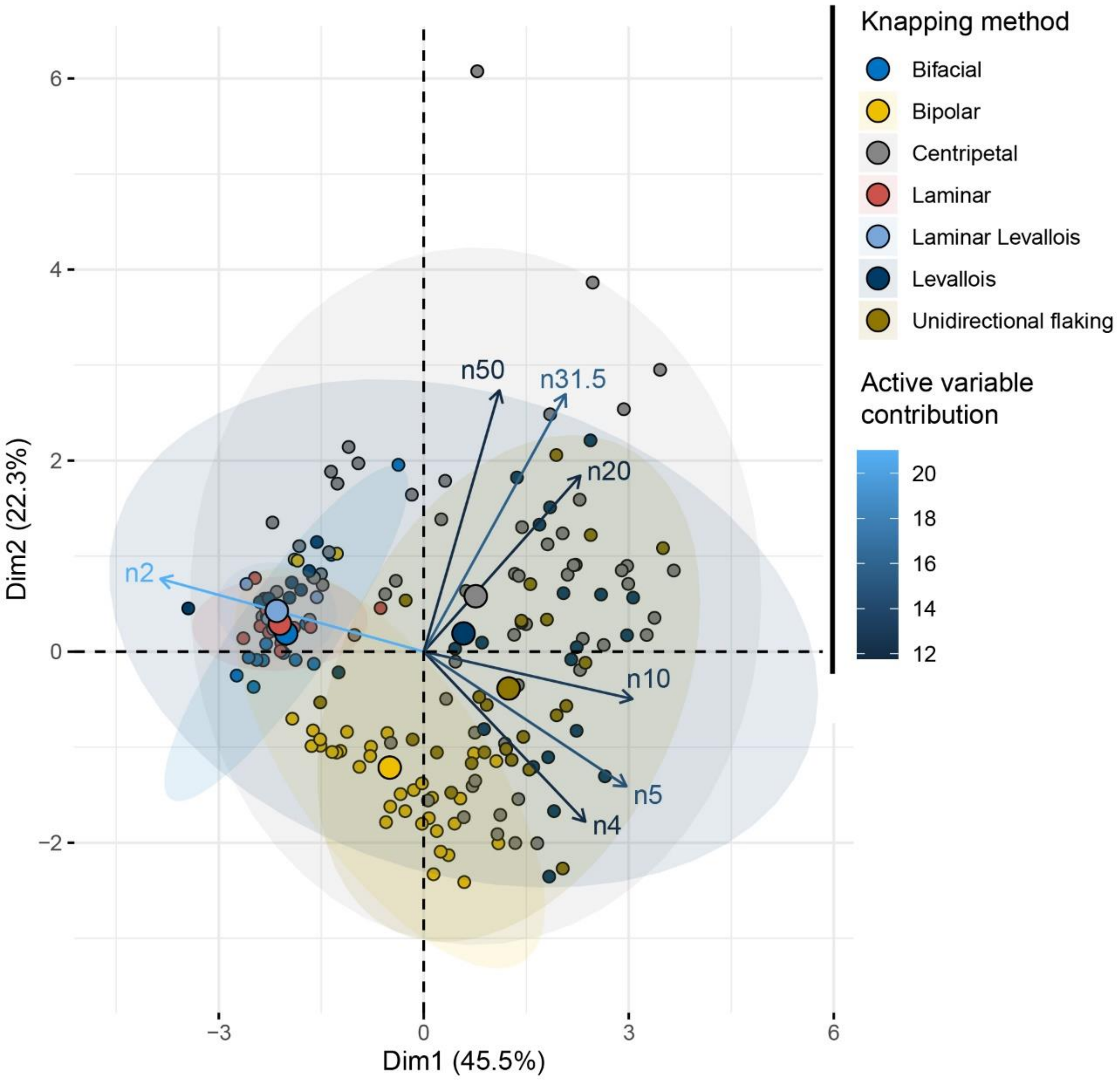
Knapping method
Bifacial
Bipolar
Centripetal
Laminar
Laminar Levallois
Levallois
Unidirectional flaking
Active variable contribution
20
18
16
14
12
n50
n31.5
n20
n2
n10
n5
n4
Dim2 (22.3%)
Dim1 (45.5%)
6
4
2
0
−2
−3
0
3
6

358

Fig 14: PCA of the SA dataset compared to the EU dataset [20,21]. The PCA has been made considering the knapping method as a variable.

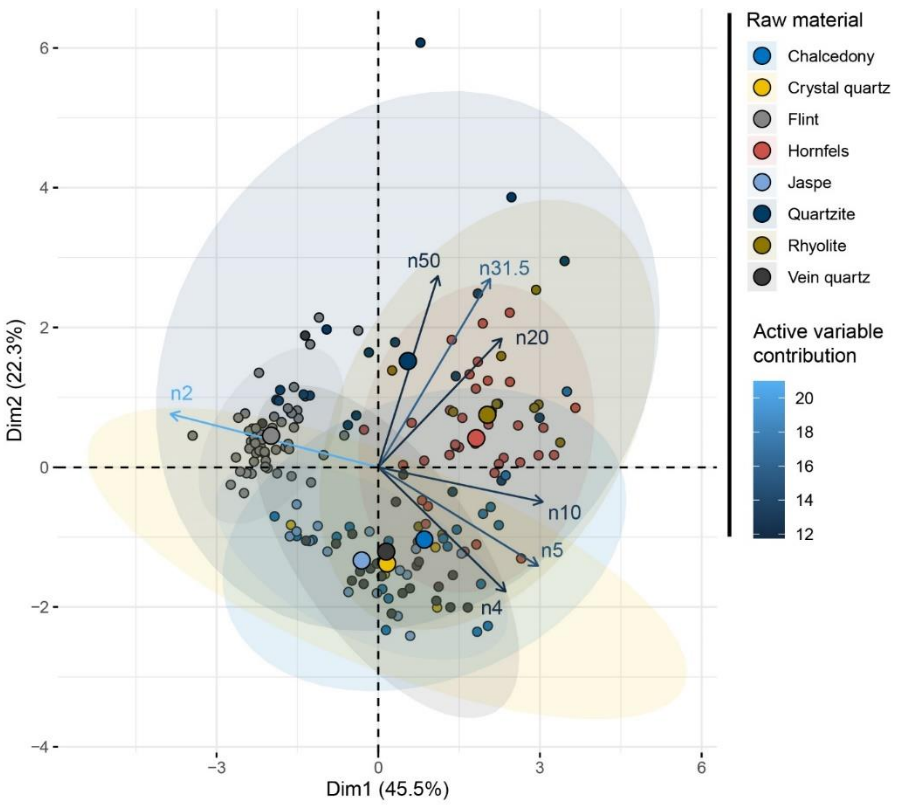

Fig 15: PCA of the SA dataset and the EU dataset [20,21], having 'raw material' as a supplementary variable.

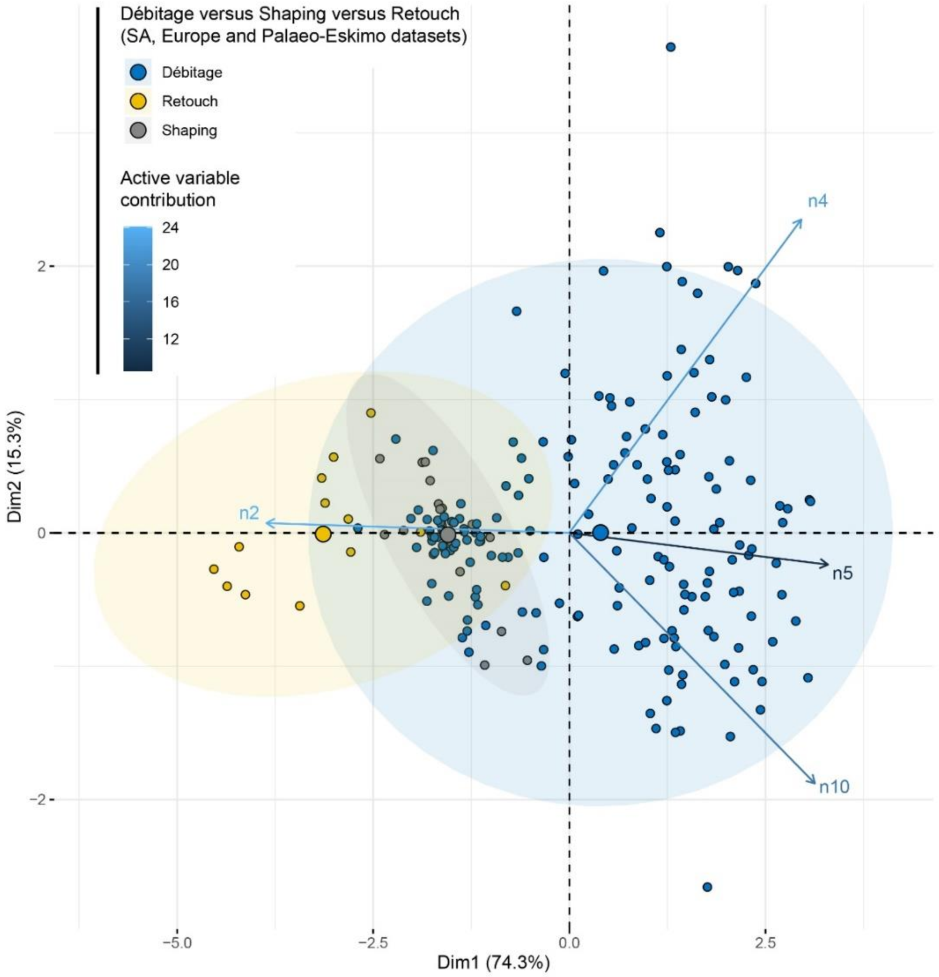

Fig 16: PCA comparing debitage, shaping and retouch from the SA dataset, EU dataset, and ESKI dataset [20,21].

Is the particle size distribution of the SA dataset different from the RUNOFF dataset?

The factorial space of the SA dataset compared to the RUNOFF model [4] (Fig 17) shows a clear opposition on the first dimension (53% of the variability) between, on one hand, the fine accumulation area composed of smaller remains (2 to 4 mm size class) and, on the other hand, knapping methods (SA dataset) and runoff areas as residual and transit (in and off channel) described by particles over 4 mm wide. The SA experiments overlap with most of the runoff accumulations of large particles.

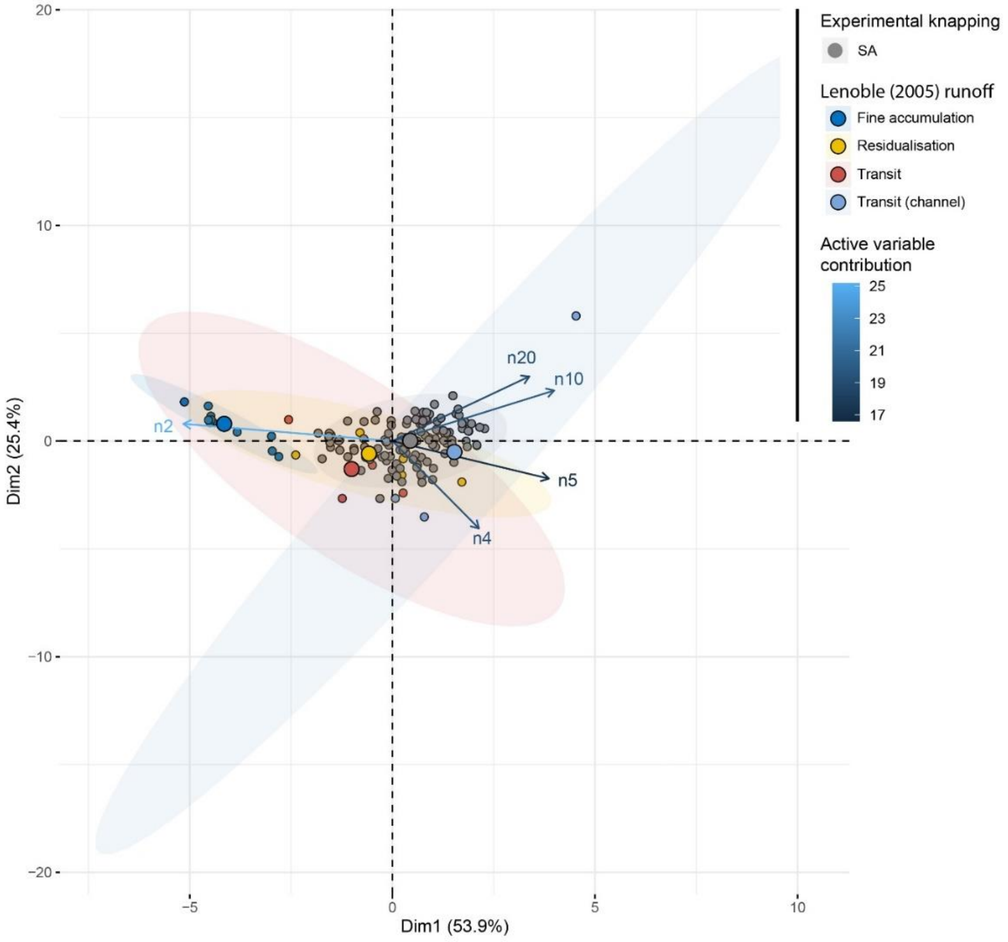

Fig 17: PCA comparing the SA dataset to the RUNOFF dataset [4].

Discussion

Variability within the SA dataset

Patterson [41]: p. 550 highlighted the need for ‘simple analytical techniques’ for examining lithic assemblages that can be used by any archaeologist (and not only lithic specialists). This still seems to be a valid point today as lithic assemblages have the advantage of preservation over organic remains, and they provide the most evident continuous cultural record available from the past [42–44].

The results of our experiment highlight two main points within the SA dataset: a distinction between raw materials and a difference between debitage produced by freehand and bipolar knapping techniques.

Concerning the raw materials, we see, on the one hand, that in our experimental sample chalcedony, X-quartz, A-quartz, and jasper group more or less together and produce smaller size particles; on the other hand, we find rhyolite, quartzite and hornfels producing wider size ranges (Figs 8 and 10). Other authors like K. Schick [2] have also highlighted differences regarding raw materials. Our results prove that experiments using specific raw materials from the regions associated with archaeological assemblages are needed to understand particle size distribution in Stone Age lithic assemblages.

Regarding the distinction between freehand and bipolar knapping methods, from a particle size analysis point of view, it seems possible to distinguish between these two types of reduction. This is in accordance with previous characterizations made to quantitatively distinguish freehand from bipolar axial knapping [13,45,46]. This should be tested in future archaeological analyses where freehand and bipolar knapping have been documented within the same assemblage, such as the Howiesons Poort of Sibudu [47,48], and the miniaturization strategies at Boomplaas [49]. It would be interesting to test if this distinction between freehand and bipolar is feasible considering that the distinction has been informed by the multivariate analysis of the particle size. The reason we mention previous technological analyses is that the main way of identifying bipolar knapping in those studies was through the cores, as it seems the easier way to recognize the reduction technique [50]. If distinguishing between freehand and bipolar knapping by means of particle size is confirmed, this experiment would have made a significant contribution to the field.

Regarding the different freehand knapping methods in our sample, in the factorial space, it does not seem possible to differentiate unidirectional, discoidal and *Levallois* flaking systems. However, as we have not knapped each raw material through all knapping methods, it remains difficult to know what variable (either the knapping method or the raw material) causes these differences. Our results show that the size distribution in southern African archaeological assemblages is knapping method specific and raw material specific.

Due to the variability expressed by the SA dataset, the selection of a single raw material and knapping method appropriate to the site context provides a more accurate model for performing a particle size analysis. Moreover, when combining a specific raw material and a specific knapping method, the

distribution areas tend to reduce (with and without 95% confidence intervals) in both ternary plots and principal component analyses.

Differences between SA, EU and ESKI datasets

Southern African lithic industries produce a greater proportion of bigger particles whatever the raw material and the knapping method (Fig 13). In the southern African dataset, the smallest particles (2 to 4 mm) always constitute 50 to 60% of the reduction sequences. In the EU dataset [20,21] (see Fig 13 therein) the percentage of this fraction was slightly higher, between 60-70% in all their variants.

It seems that our knapping and raw material particle size distribution differs from the ones published in Bertran and colleagues [20,21]. In the case of the EU dataset [20,21], the different experiments overlap remarkably in the multivariate analysis and in the ternary plots (Fig 11), whereas our dataset has a much wider distribution (Fig 13).

The EU, ESKI and SA datasets are clearly separated from each other. The reasons for these different groupings might be due to different aspects. With the evidence at hand, we can only propose some hypotheses that should be tested in future experiments.

Firstly, some technical behaviours produce more small particles. This is particularly the case of microlithic production and retouching activities (ESKI dataset) and to a lesser degree, for bipolar knapping on anvil (SA dataset) and shaping (EU and ESKI datasets). Similar results have been pointed out in other experimental studies [2,20,21,41].

Secondly, the differences between Bertran and colleagues [20,21] and our sample regarding freehand variants could be the result of the level of skill of the knappers, due to specific knapper gestures and procedures. In other words, it should not exclude the hypothesis that knapper competence and experience have an impact on particle size distribution. Nonetheless, these different levels of knapping performance enrich the models, as it is expected that in the past there would also have been variability in knapping skills within cultural traditions and periods. Moreover, despite similar knapping goals,

knapper-specific gestures, habits, and procedures (such as platform preparation or removing striking platform overhang) could also affect size distribution. Again, these variables would require additional experiments.

Thirdly, raw material specificities also induce different patterns in terms of particle size distribution. Whereas flint knapping experiments have a narrow distribution, other types of raw material induce specific distributions with various dispersion features. Regardless of the knapping method, flint tends to produce finer particles than A-quartz, X-quartz, jasper, and chalcedony, which in turn produce finer particles than quartzite, rhyolite and hornfels. Finer raw materials (i.e., flint, jasper, and chalcedony), A-quartz and X-quartz produce finer grain sizes in contrast to coarser metamorphic and volcanic raw materials like quartzite, hornfels and rhyolite.

Finally, considering previous knapping datasets [20,21] and our knapping experiments, it seems clear that blank transformation ("retouching") produces smaller particles than "shaping" and blank production (debitage). The intensity of retouch and site function [34] also influence the particle size distribution of industries as is already shown in Bertran et al. [20–22]. When a great proportion of the blank is transformed on site, we would expect a greater proportion of the small fraction (2 to 4 mm).

SA knapping dataset versus RUNOFF experiments

The comparison of the SA dataset distribution and the RUONFF model [4] (for flint) shows a remarkable amount of overlap.

While taken as a whole, the SA dataset shows a broad distribution (contra EU dataset) (Fig 13 and 17). At a site scale, it is nevertheless possible to reduce that distribution by considering only a single knapping method on a single raw material. This will reduce the overlap with the RUNOFF model and allow a more accurate comparison to assess the degree of perturbation in the archaeological record. The overlap between the SA and the RUNOFF dataset could also be due to the different densities of the raw materials. Igneous rocks, such as rhyolite are denser that sedimentary rocks and this could have an

impact on this general comparison. This overlapping is also pointing out the need for further experimentation that clarifies the potential reasons for this situation. The new experiments should take into account the geomorphological frequent scenarios for site formation deposits in southern Africa assemblages.

While our experiment has produced very concrete results, the size distribution analysis of lithic material is not sufficient on its own to assess the perturbation of an archaeological lithic assemblage and must be coupled with other analytical tools. In this regard, sedimentary analysis [3], fabric analysis [52–54], refitting [17,55–58], spatial analysis [59,60], material proportions [51], faunal analysis in terms of taphonomy [62], and dating [5] are all tools that can help characterize the impact of natural processes on archaeological assemblages. When all of them are combined [51,59], we can reach a very detailed site formation model by which to gauge the degree of perturbation of the associated lithic assemblage.

Conclusion

Our results highlight the need to perform more experimentation to clarify and further support the preliminary distinctions proposed here and the contradictions with previous experimental work.

While all knapping experiments show the same general pattern of size distribution (following a Weibull function, i.e., proportion decreasing as size increases [23]), we have demonstrated that the knapping method, blank transformation activity intensity, and raw material, explain most of the variability. The creation of a new experimental knapping dataset focusing on southern African debitage and raw materials, and its comparison with previous datasets have shown a more complex picture for particle size analysis than was previously thought. There are two main aspects that do not match with recent investigations on particle size analysis in Europe: on the one hand the wider distribution of our experimental knapping, on the other hand the huge overlap between our SA dataset and the RUNOFF dataset. To reduce this overlap, the experimental debitage model compared to the RUNOFF dataset should be knapping method and raw material specific. This means that the performance of a particle size analysis at a site must be done in combination with a technological study of the lithic material. The

latter guides the choice of the knapping method and raw material to be included in the comparison model between experimental knapping and runoff experiences on sorting. Both analyses are clearly dependent on one another (i.e., particle size distribution and technology). The first conditions the interpretation of the second and the second makes the first more accurate.

Acknowledgements

We thank Gary Trower and Ben Maclennan for indicating to us raw material outcrops and providing us with some of the raw material we used in our knapping experiments. Our thanks also go to Lucinda Backwell, Tammy Hodgskiss, Matt Caruana and Matt Lotter, whose reading and correction of the article were very valuable.

References

1. Schiffer MB. Toward the identification of formation processes. American Antiquity. 1983;48: 675–706.

2. Schick KD. Stone Age sites in the making: experiments in the formation and transformation of archaeological occurrences. BAR Publishing; 1986.

3. Texier J. A propos des processus de formation des sites préhistoriques/About prehistoric site formation processes. Paléo, Revue d'Archéologie Préhistorique. 2000;12: 379–386.

4. Lenoble A. Ruissellement et formation des sites préhistoriques: référentiel actualiste et exemples d'application au fossile. British Archaeological Report; 2005.

5. Bertran P, Todisco D, Bordes J-G, Discamps E, Vallin L. Perturbation assessment in archaeological sites as part of the taphonomic study: a review of methods used to document the impact of natural

processes on site formation and archaeological interpretations. PALEO Revue d'archéologie préhistorique. 2019; 52–75.

6. Bishop WW. Paleogeomorphology and continental taphonomy. Fossils in the Making: Vertebrate Taphonomy and Paleoecology, Midway Reprint. 1980; 20–37.

7. Petraglia MD, Nash DT. The impact of fluvial processes on experimental sites. Natural formation processes and the archaeological record. BAR; 1987. pp. 108–130.

8. Petraglia MD, Potts R. Water flow and the formation of Early Pleistocene artifact sites in Olduvai Gorge, Tanzania. Journal of Anthropological Archaeology. 1994;13: 228–254.

9. Bertran P, Klaric L, Lenoble A, Masson B, Vallin L. The impact of periglacial processes on Palaeolithic sites: the case of sorted patterned grounds. Quaternary International. 2010;214: 17–29.

10. Lenoble A, Bertran P, Boulogne S, Masson B, Vallin L. Évolution des niveaux archéologiques en contexte périglaciaire. Apport de l'expérience Gavarnie. Les Nouvelles de l'archéologie. 2009; 16–20.

11. Bordes F, Bourgon M. Le complexe moustérien: Moustériens, levalloisien et tayacien. l'Anthropologie. 1951;55: 1–23.

12. de la Peña P, Witelson D. Trampling vs. retouch in a lithic assemblage: a case study from a middle stone age site, steenbokfontein 9KR (Limpopo, South Africa). Journal of Field Archaeology. 2018;43: 522–537.

13. Eren MI, Durant A, Neudorf C, Haslam M, Shipton C, Bora J, et al. Experimental examination of animal trampling effects on artifact movement in dry and water saturated substrates: a test case from South India. Journal of Archaeological Science. 2010;37: 3010–3021.

14. Gifford DP, Behrensmeyer AK. Observed formation and burial of a recent human occupation site in Kenya. Quaternary Research. 1977;8: 245–266.

15. Gifford DP. Ethnoarchaeological observations of natural processes affecting cultural materials. Explorations in Ethno-archaeology. 1978; 77–101.

16. Marwick B, Hayes E, Clarkson C, Fullagar R. Movement of lithics by trampling: an experiment in the Madjedbebe sediments, northern Australia. Journal of Archaeological Science. 2017;79: 73–85.

17. Villa P, Courtin J. The interpretation of stratified sites: a view from underground. Journal of Archaeological Science. 1983;10: 267–281.

18. Yellen JE. Archaeological approaches to the present: Models for reconstructing the past (Studies in archeology). 1977.

19. Sitzia L. Chronostratigraphie et distribution spatiale des dépôts éoliens quaternaires du bassin aquitain. PhD Thesis, Université Sciences et Technologies-Bordeaux I. 2014.

20. Bertran P, Claud É, Detrain L, Lenoble A, Masson B, Vallin L. Composition granulométrique des assemblages lithiques. Application à l'étude taphonomique des sites paléolithiques. PALEO Revue d'archéologie préhistorique. 2006; 7–36.

21. Bertran P, Lenoble A, Todisco D, Desrosiers PM, Sørensen M. Particle size distribution of lithic assemblages and taphonomy of Palaeolithic sites. Journal of Archaeological Science. 2012;39: 3148–3166. doi:10.1016/j.jas.2012.04.055

22. Todisco D, Bhiry N, Resrosiers P. Paleoeskimo Site Taphonomy: An Assessment of the Integrity of the Tayara Site, Qikirtaq Island, Nunavik, Canada. Geoarchaeology: An International Journal. 2009;24: 743–791.

23. Stahle DW, Dunn JE. An analysis and application of the size distribution of waste flakes from the manufacture of bifacial stone tools. World Archaeology. 1982;14: 84–97.

24. Pargeter J. Early microlithic technologies and behavioural variability in Southern Africa and South Asia. Taylor & Francis; 2017.

25. Wadley L. Those marvellous millennia: the Middle Stone Age of southern Africa. Azania: Archaeological Research in Africa. 2015;50: 155–226.

26. Crabtree DE. An introduction to Flintworking. Occasional Papers of the Idaho State University Museum. 1972.

27. de la Peña Alonso P. Sobre la identificación macroscópica de las piezas astilladas: propuesta experimental. Trabajos de Prehistoria. 2011;68: 79–98.

28. Terradas X. Discoid flaking method: conception and technological variability. BAR International Series. 2003;1120: 19–32.

29. Mourre V. Discoïde ou pas discoïde? Réflexions sur la pertinence des critères techniques définissant le débitage discoïde. BAR International Series. 2003;1120: 1–18.

30. Boëda E. Le concept Levallois: variabilité des méthodes. Archéo éditions; 1994.

31. Cairncross B. Field guide to rocks & minerals of Southern Africa. Penguin Random House South Africa; 2011.

32. Mourre V. Les industries en quartz au Paléolithique. Terminologie, méthodologie et technologie. Paléo, Revue d'Archéologie Préhistorique. 1996;8: 205–223.

33. Sunagawa I. Crystals: growth, morphology, & perfection. Cambridge University Press; 2007.

34. Rodríguez-Rellán C. La anisotropía y el clivaje del cuarzo automorfo y sus posibles efectos sobre la talla: Una revisión bibliográfica. Journal of Lithic Studies. 2015;2: 189–207.

35. Rodríguez-Rellán C. Variability of the rebound hardness as a proxy for detecting the levels of continuity and isotropy in archaeological quartz. Quaternary International. 2016;424: 191–211.

36. de la Peña P, Val A, Stratford D, Colino F, Esteban I, Fitchett J, et al. Revisiting Mwulu's Cave: new insights into the Middle Stone Age in the southern African savanna biome. Archaeological and Anthropological Sciences. 2019;11: 3239–3266.

37. Allaby M. Dictionary of earth sciences. Oxford University Press; 2008.

38. Hammer Ø, Harper DA, Ryan PD, others. PAST: Paleontological statistics software package for education and data analysis. Palaeontologia electronica. 2001;4: 9.

39. Weaver TD, Boyko RH, Steele TE. Cross-platform program for likelihood-based statistical comparisons of mortality profiles on a triangular graph. Journal of Archaeological Science. 2011;38: 2420–2423.

40. Lê S, Josse J, Husson F. FactoMineR: an R package for multivariate analysis. Journal of statistical software. 2008;25: 1–18.

41. Patterson LW. Characteristics of bifacial-reduction flake-size distribution. American Antiquity. 1990;55: 550–558.

42. Shea JJ. Prehistoric stone tools of Eastern Africa: a Guide. Cambridge University Press; 2020.

43. Shott MJ. Size and form in the analysis of flake debris: review and recent approaches. Journal of archaeological method and theory. 1994;1: 69–110.

44. Stout D. Stone toolmaking and the evolution of human culture and cognition. Philosophical Transactions of the Royal Society B: Biological Sciences. 2011;366: 1050–1059.

45. Pargeter J, Eren MI. Quantifying and comparing bipolar versus freehand flake morphologies, production currencies, and reduction energetics during lithic miniaturization. Lithic Technology. 2017;42: 90–108.

46. Pargeter J, de la Peña P. Milky quartz bipolar reduction and lithic miniaturization: experimental results and archaeological implications. Journal of Field Archaeology. 2017;42: 551–565.

47. de la Peña P, Wadley L. Quartz knapping strategies in the Howiesons Poort at Sibudu (KwaZulu-Natal, South Africa). PloS one. 2014;9: e101534.

48. de la Peña P. The interpretation of bipolar knapping in African Stone Age studies. Current Anthropology. 2015;56: 911–923.

49. Pargeter J. Lithic miniaturization in late Pleistocene southern Africa. Journal of Archaeological Science: Reports. 2016;10: 221–236.

50. Diez-Martín F, Sánchez P, Domínguez-Rodrigo M, Mabulla A, Barba R. Were Olduvai hominins making butchering tools or battering tools? Analysis of a recently excavated lithic assemblage from BK (Bed II, Olduvai Gorge, Tanzania). Journal of Anthropological Archaeology. 2009;28: 274–289.

51. Thomas M, Discamps E, Gravina B, Texier J-P. Analyse taphonomique et spatiale de palimpsestes d'occupations moustériennes de l'abri inférieur du Moustier (Dordogne, France). PALEO Revue d'archéologie préhistorique. 2019; 278–299.

52. Bertran P, Lenoble A. Fabriques des niveaux archéologiques : méthode et premier bilan des apports à l'étude taphonomique des sites paléolithiques. PALEO Revue d'archéologie préhistorique. 2002; 13–28. doi:10.4000/paleo.1383

53. Isaac GL. Towards the interpretation of occupation debris: some experiments and observations. Kroeber Anthropological Society Papers. 1967;37: 31–57.

54. McPherron SP. Additional statistical and graphical methods for analyzing site formation processes using artifact orientations. PLOS ONE. 2018;13: e0190195. doi:10.1371/journal.pone.0190195

55. Bordes J-G. Lithic taphonomy of the Châtelperronian/Aurignacian interstratifications in Roc de Combe and Le Piage (Lot, France). The chronology of the Aurignacian and of the transitional technocomplexes Dating, stratigraphies, cultural implications. 2003; 223–244.

56. Bordes J-G. À propos de taphonomie lithique: fondements épistémologiques et outils analytiques. Réflexions à partir du thème de la transition du Paléolithique moyen au Paléolithique supérieur. Université de Bordeaux. 2012.

57. Staurset S, Coulson S. Sub-surface movement of stone artefacts at White Paintings Shelter, Tsodilo Hills, Botswana: Implications for the Middle Stone Age chronology of central southern Africa. Journal of Human Evolution. 2014;75: 153–165.

58. Villa P. Conjoinable pieces and site formation processes. American Antiquity. 1982;47: 276–290.

59. Discamps E, Bachellerie F, Baillet M, Sitzia L. The use of spatial taphonomy for interpreting Pleistocene palimpsests: an interdisciplinary approach to the Châtelperronian and carnivore occupations at Cassenade (Dordogne, France). Paleoanthropology. 2019;2019: 362–388.

60. Lenoble A, Texier J-P. Processus géologiques de formation du site d'Isturitz (Sud-Ouest de la France). Implications archéologiques. PALEO Revue d'archéologie préhistorique. 2016; 235–252.

61. Thomas M, Gravina B. Analyse techno-économique d'un assemblage Discoïde du Moustérien récent de l'abri inférieur du Moustier (Dordogne, France). PALEO Revue d'archéologie préhistorique. 2019; 300–317. doi:10.4000/paleo.4933

62. Stratford, D., Clark, J.L., Wojcieszak, M., Wadley, L., d'Errico, F., de la Peña, P. et al. Geoarchaeology and zooarchaeology of Border Cave, South Africa: Initial multiproxy considerations of stratigraphy and site formation processes from the Backwell et al. excavations. Quaternary Science Reviews. 2022; p107618.

Click here to access/download;Figure;fig. 2.jpg

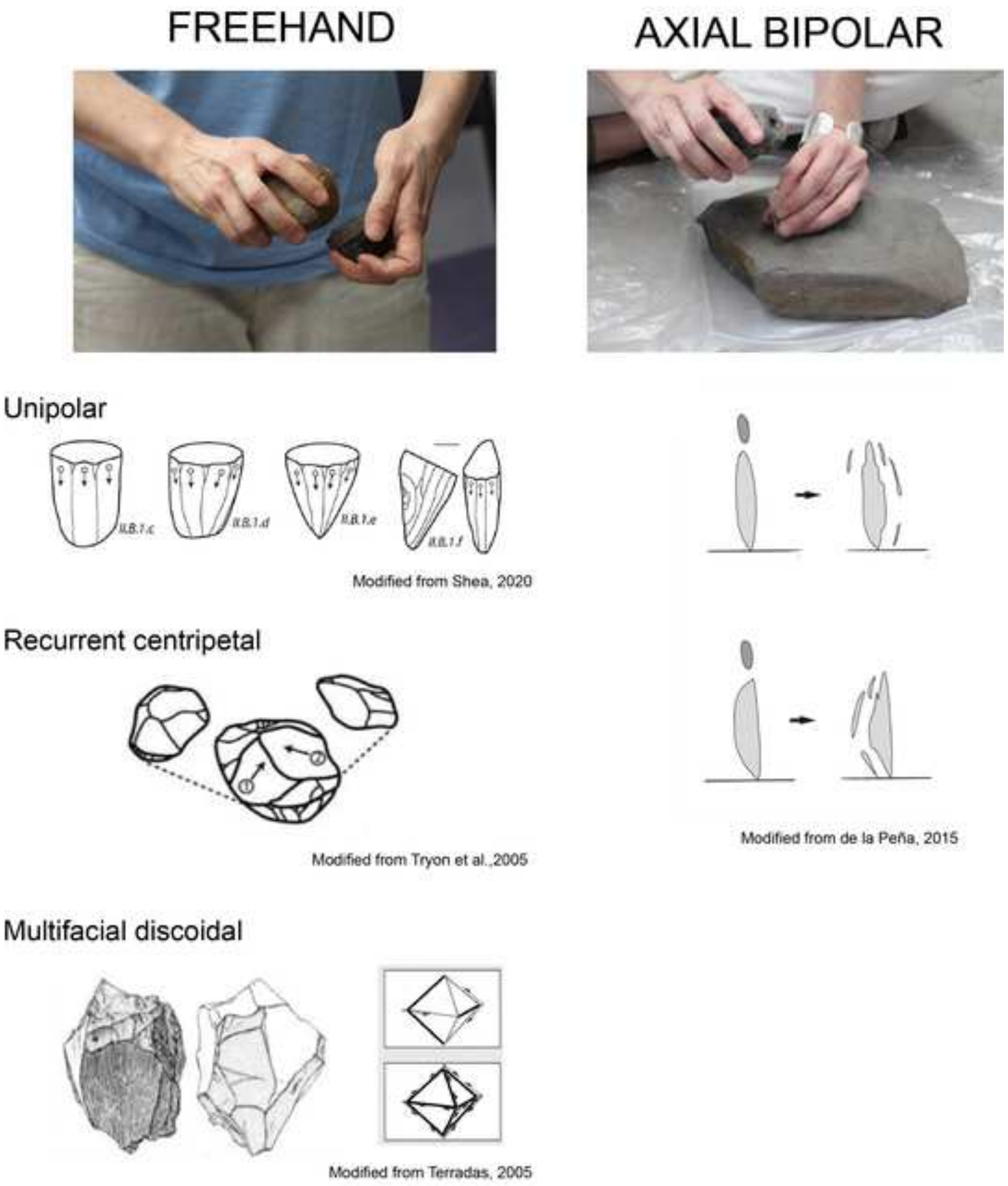

Click here to access/download;Figure;fig. 3.jpg

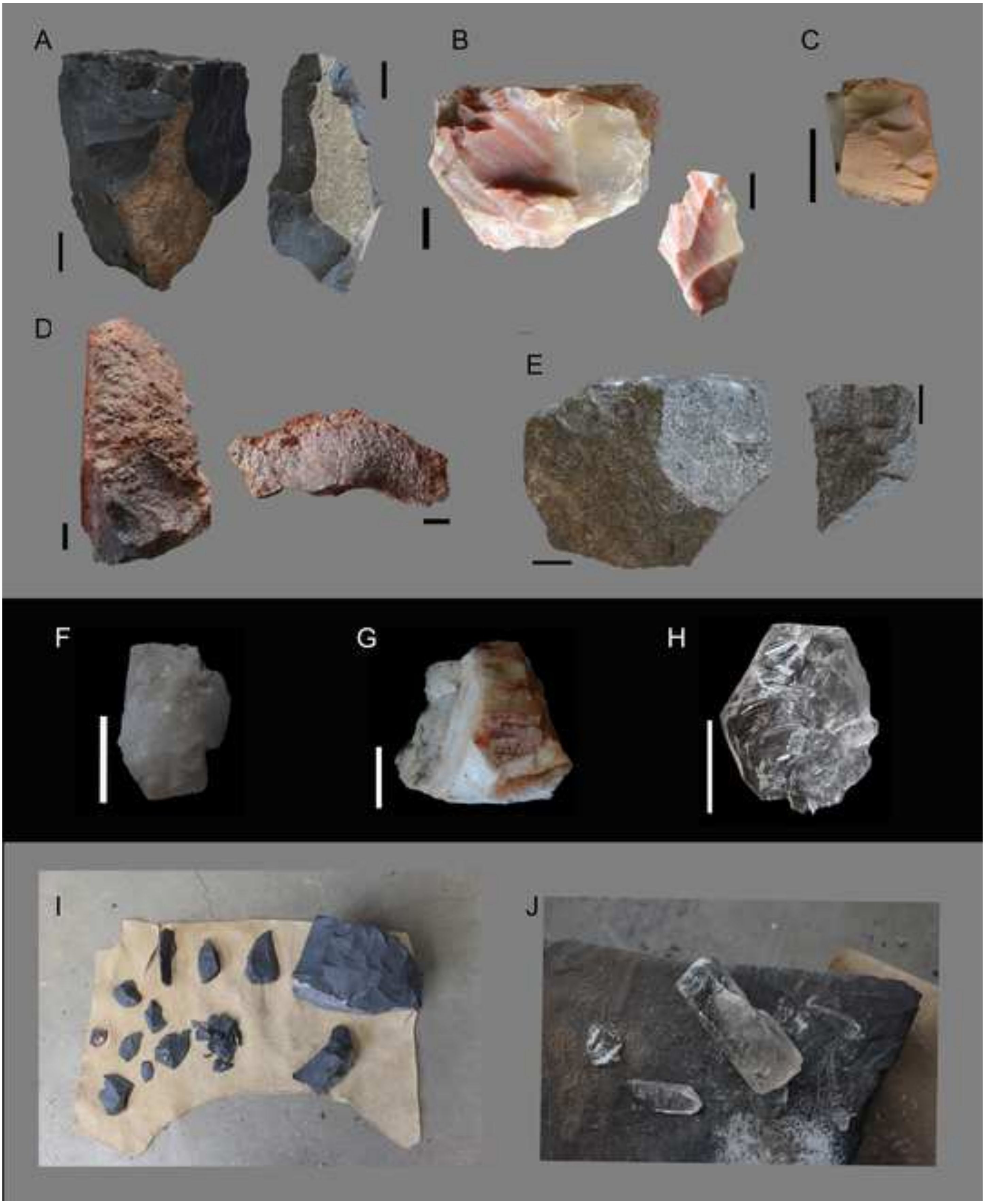

Click here to access/download;Figure;fig. 4.jpg

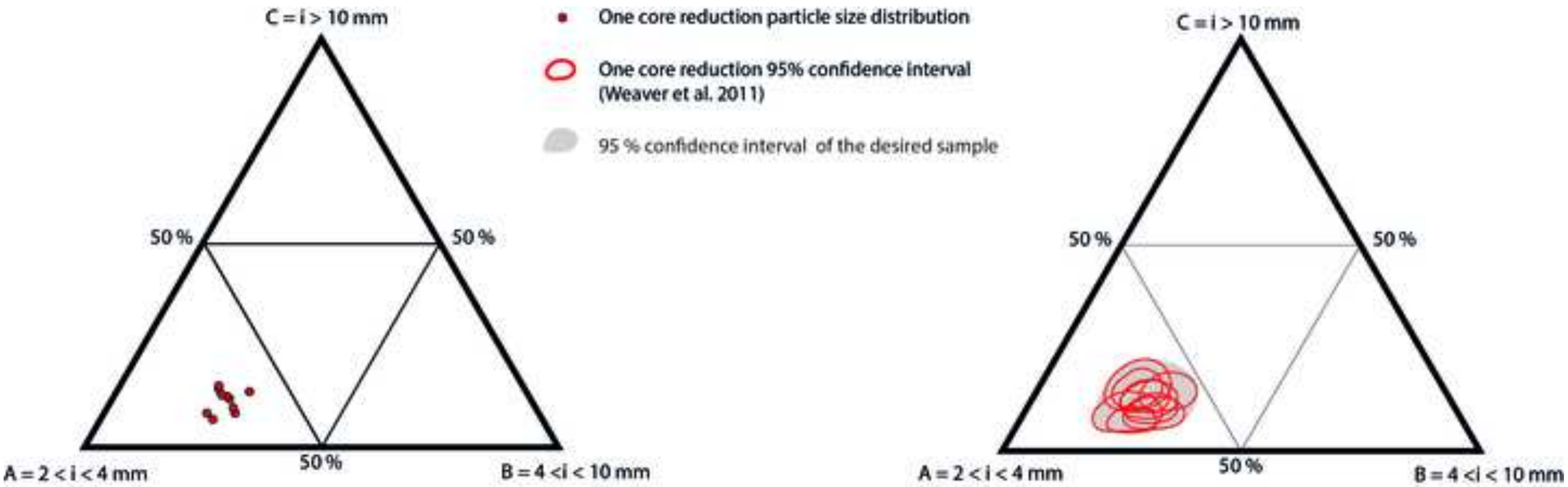

Click here to access/download;Figure;Fig5.tif

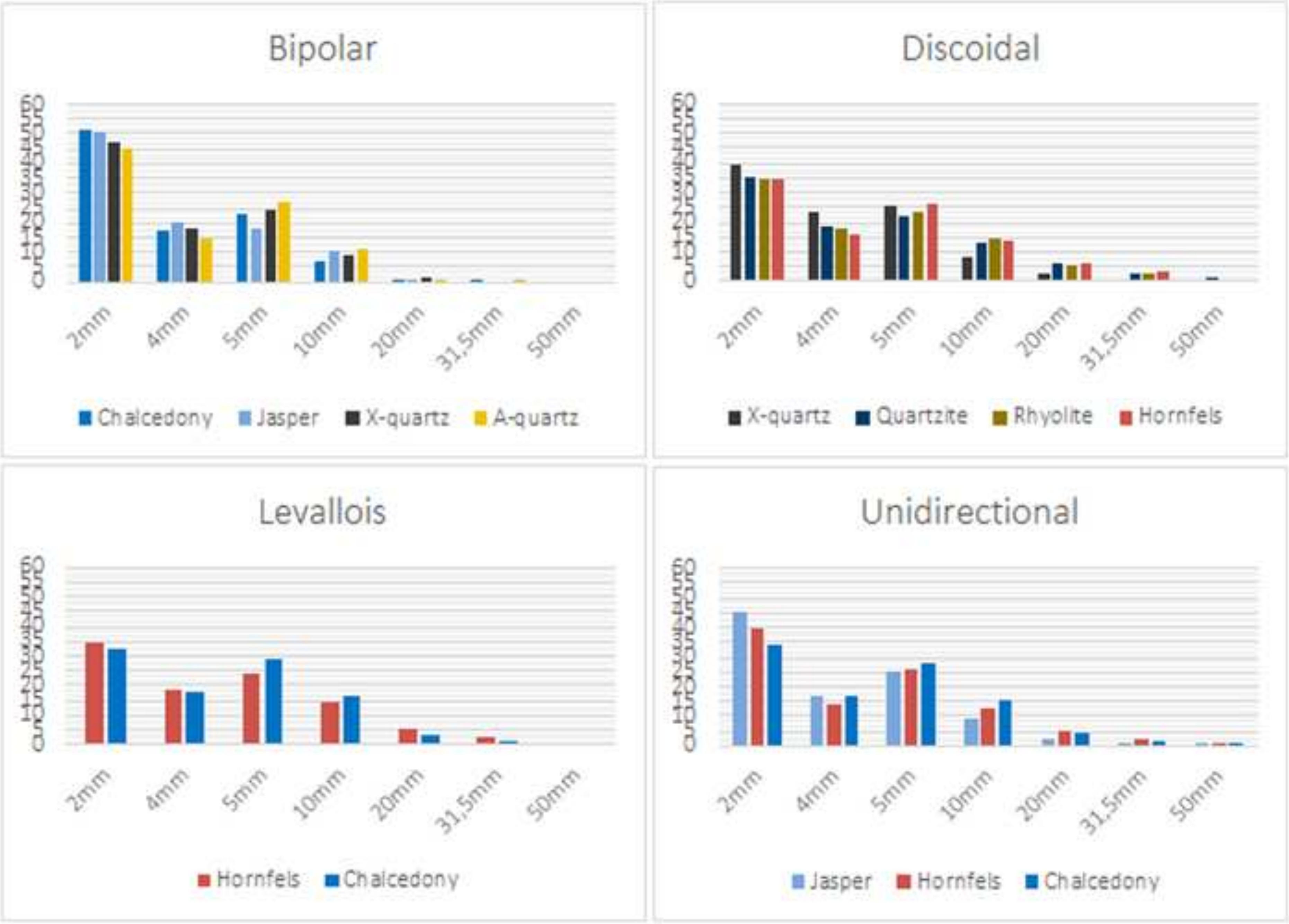

Click here to access/download;Figure;fig. 7.jpg

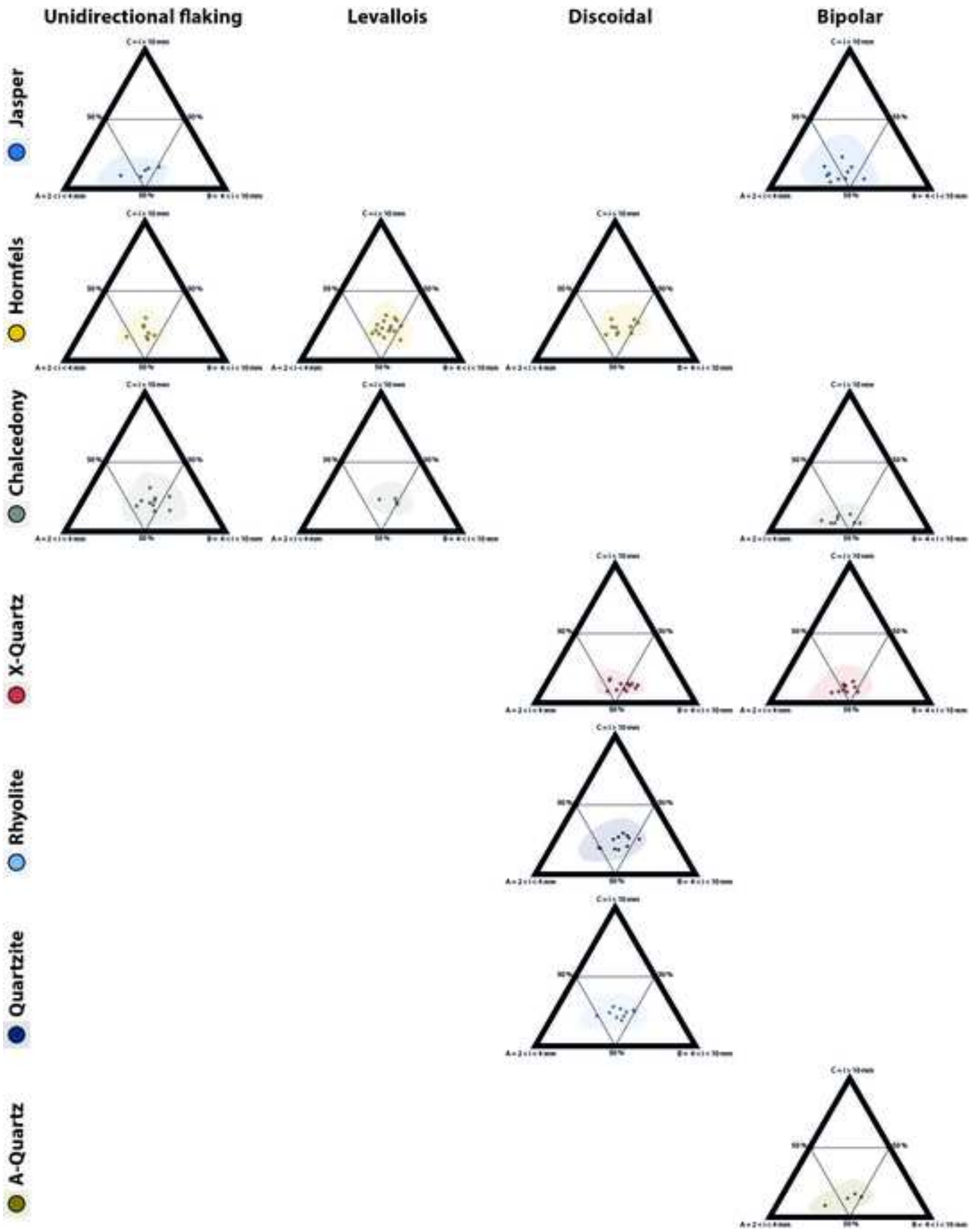

Click here to access/download;Figure;fig. 8.jpg

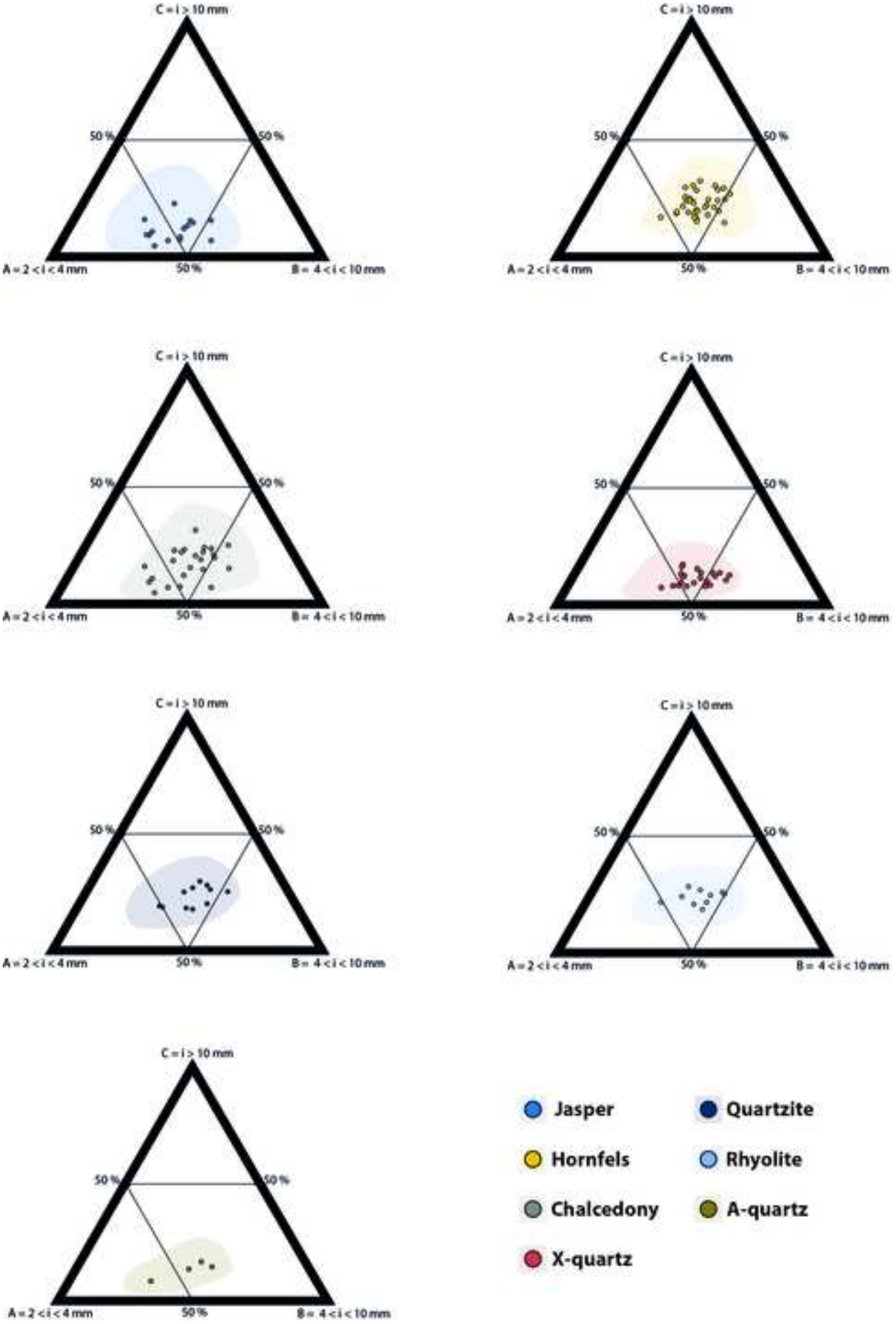

Click here to access/download;Figure;fig. 9.jpg

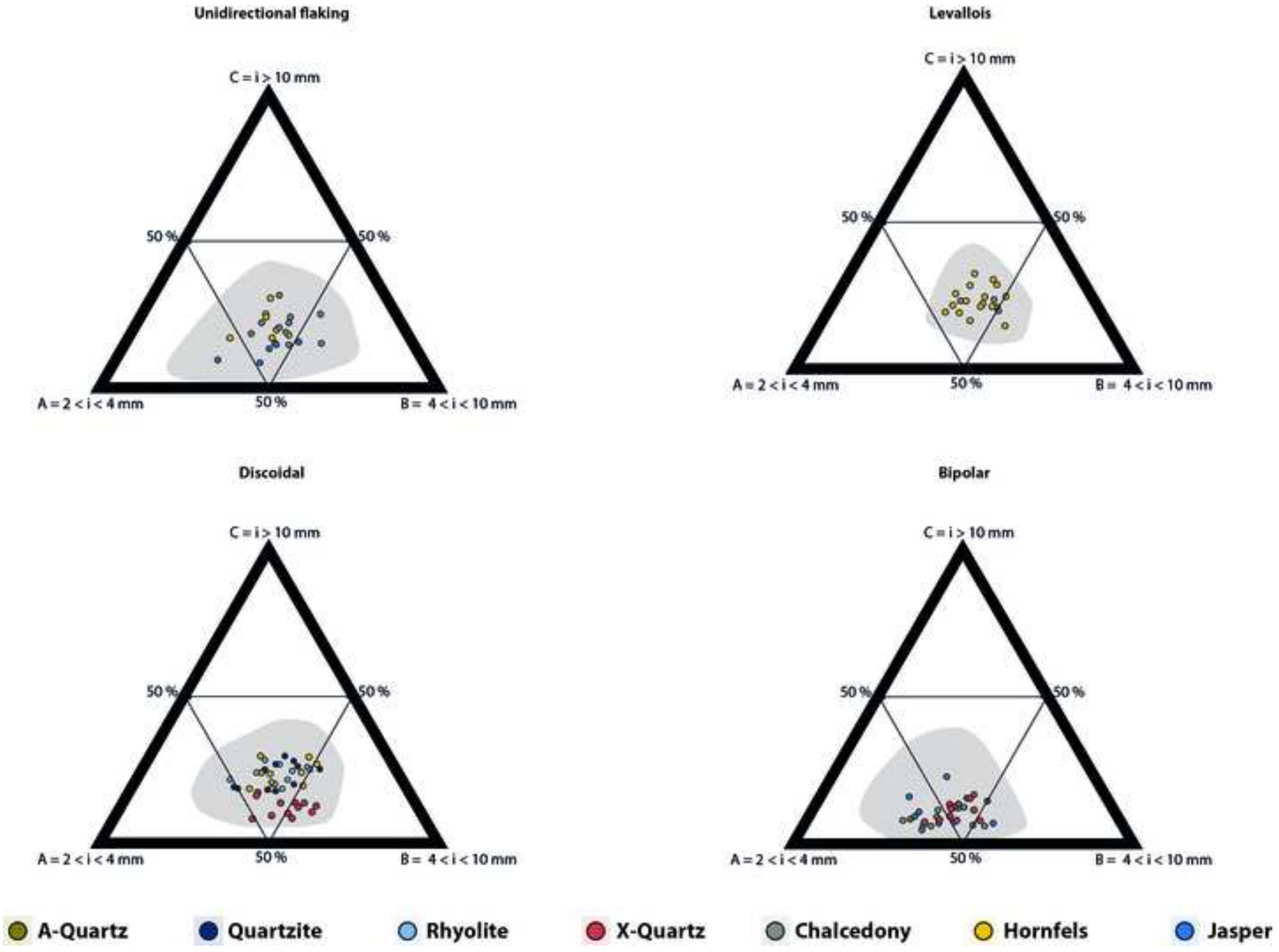

Click here to access/download;Figure;fig. 10.jpg

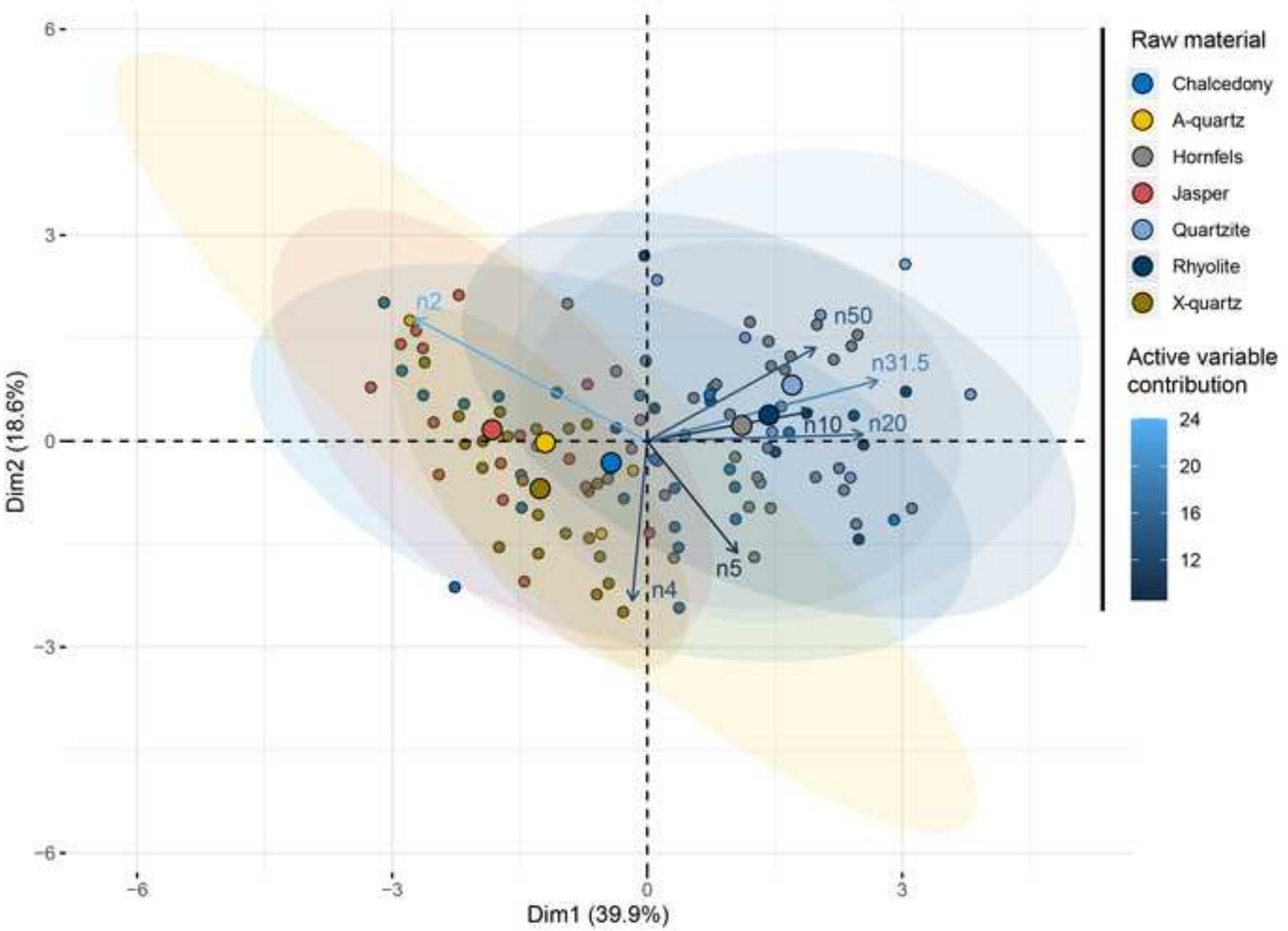

Click here to access/download;Figure;fig. 11.jpg

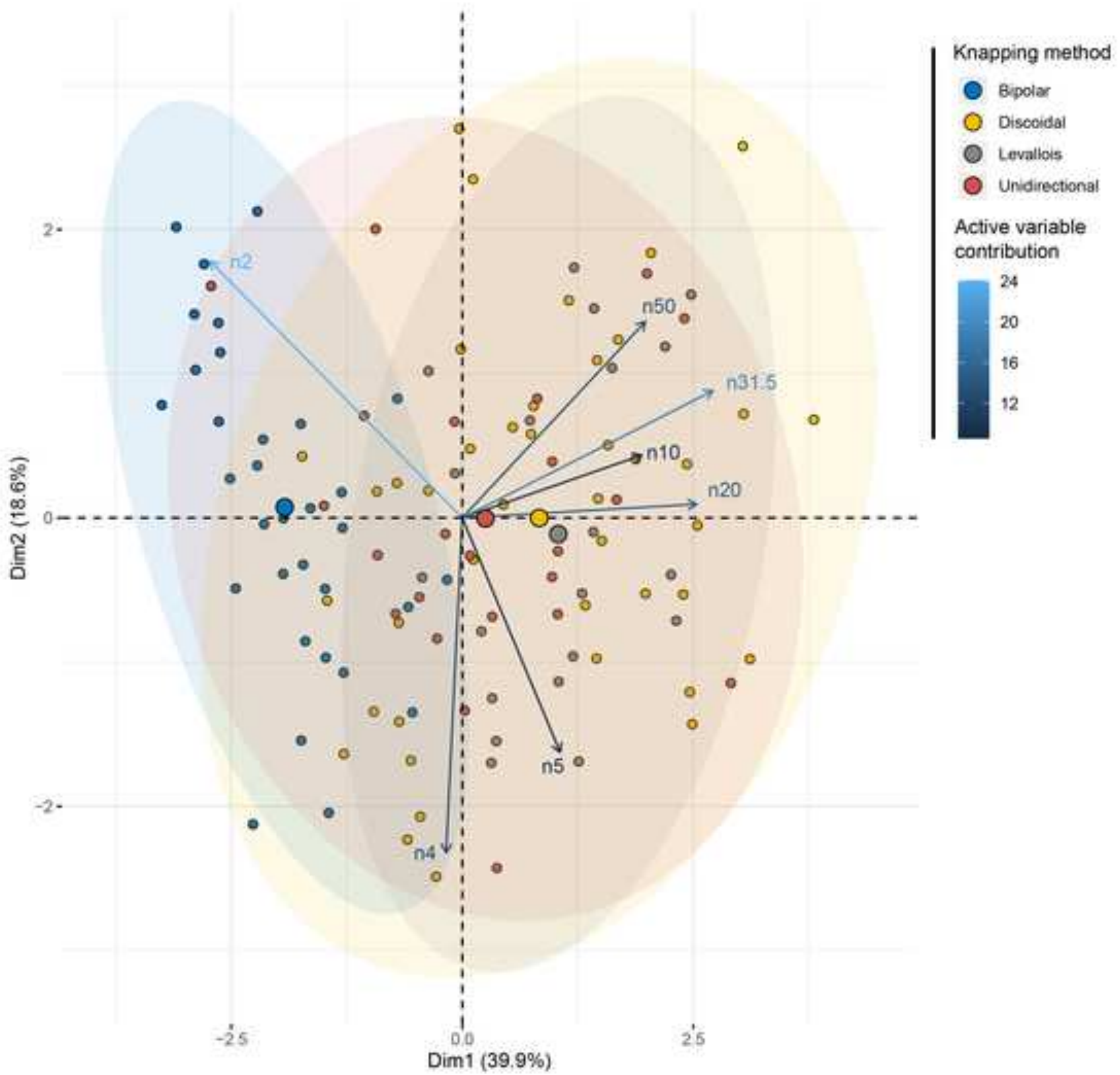

Click here to access/download;Figure;fig. 12.jpg

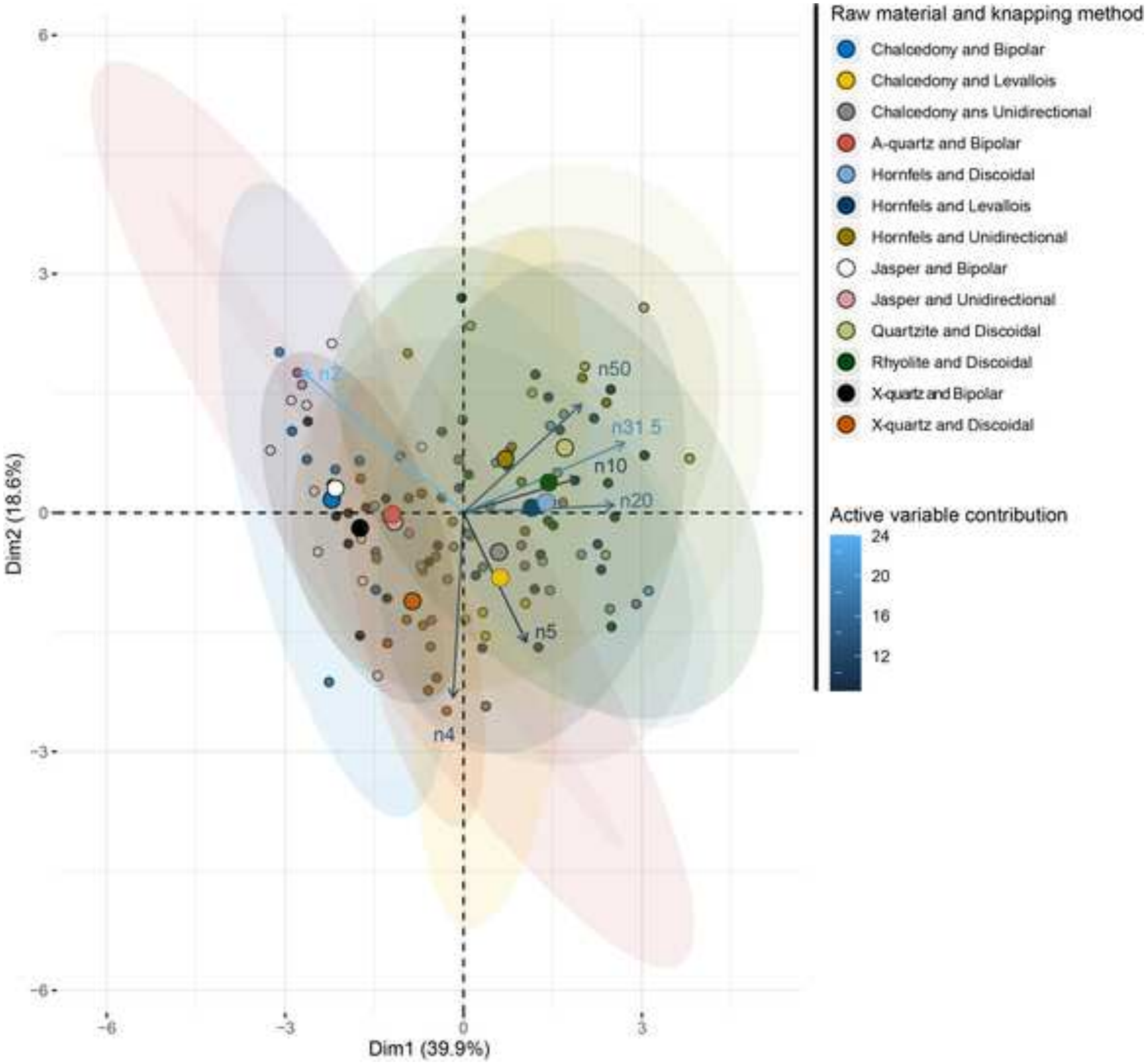

Click here to access/download;Figure;fig. 13.jpg

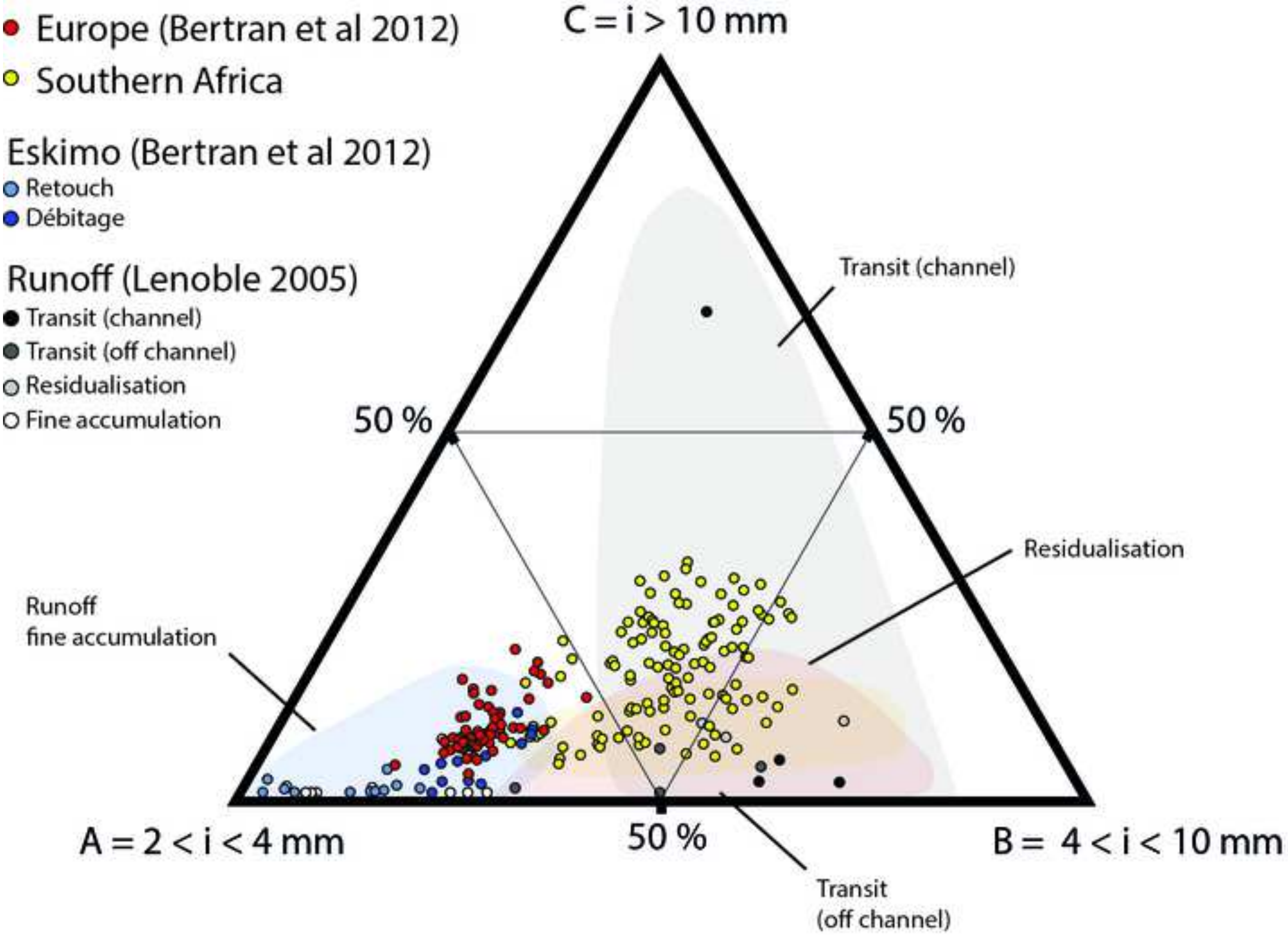

Click here to access/download;Figure;fig. 15.jpg

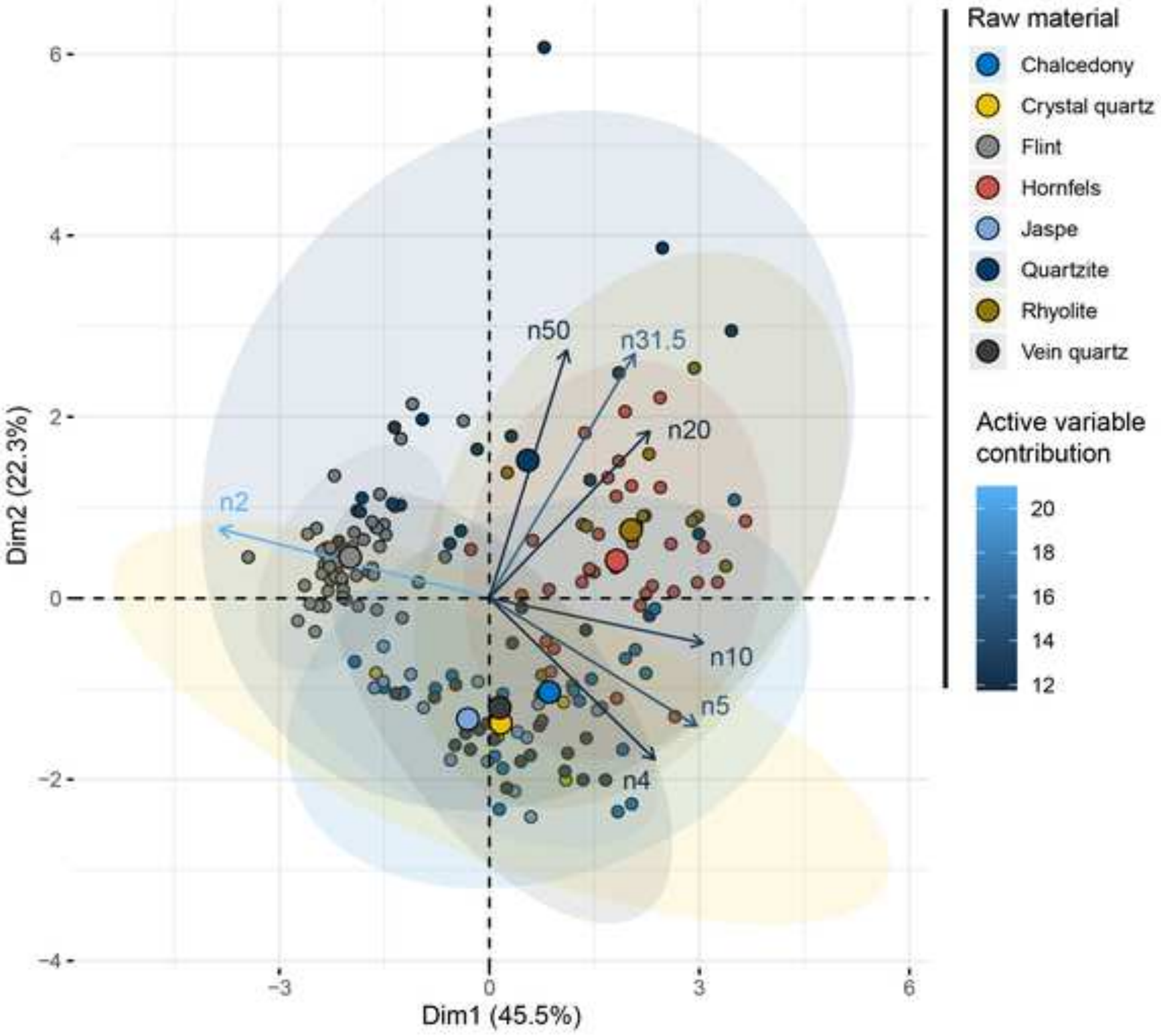

Click here to access/download;Figure;fig. 16.jpg

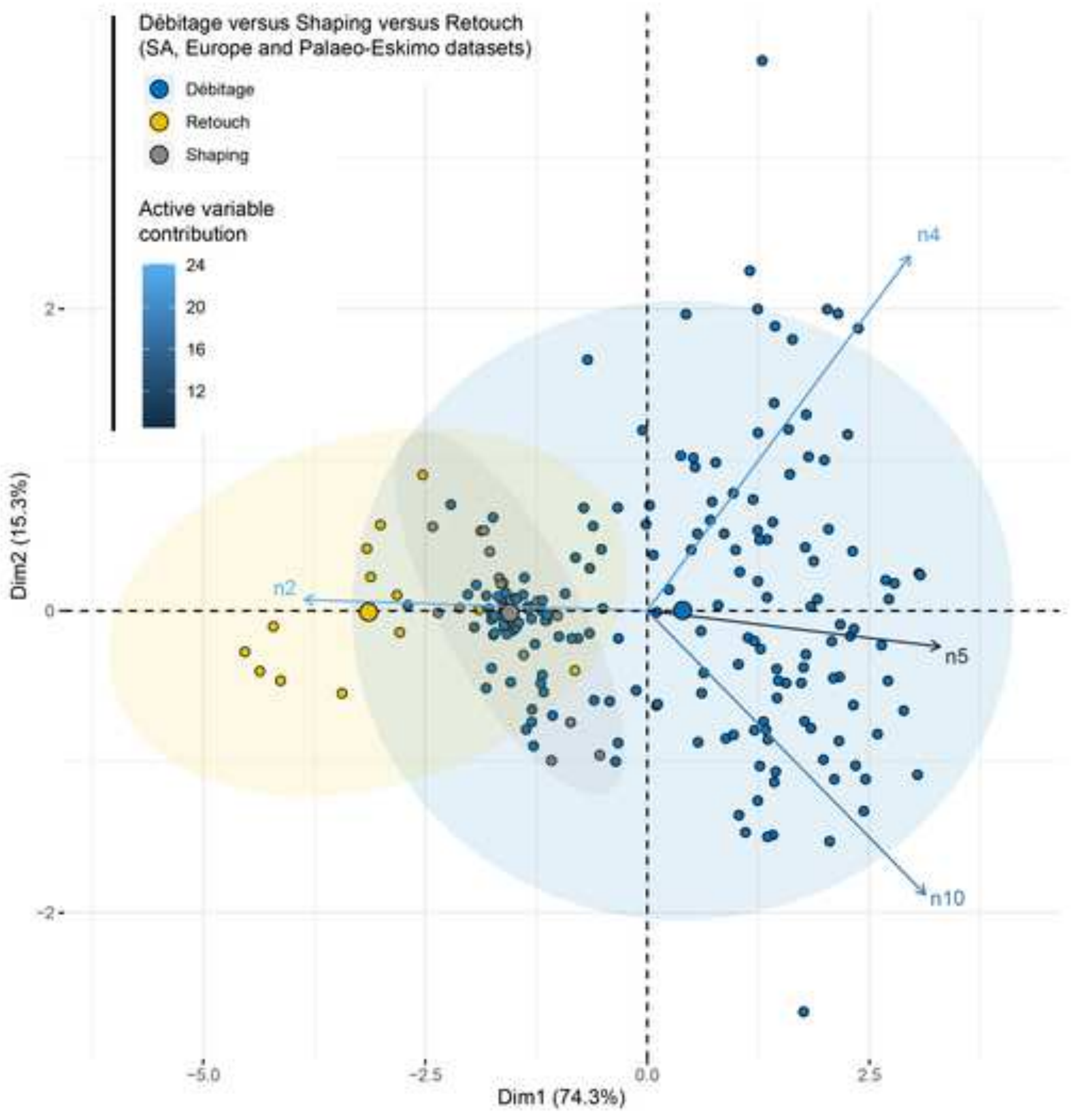

Click here to access/download;Figure;fig. 17.jpg

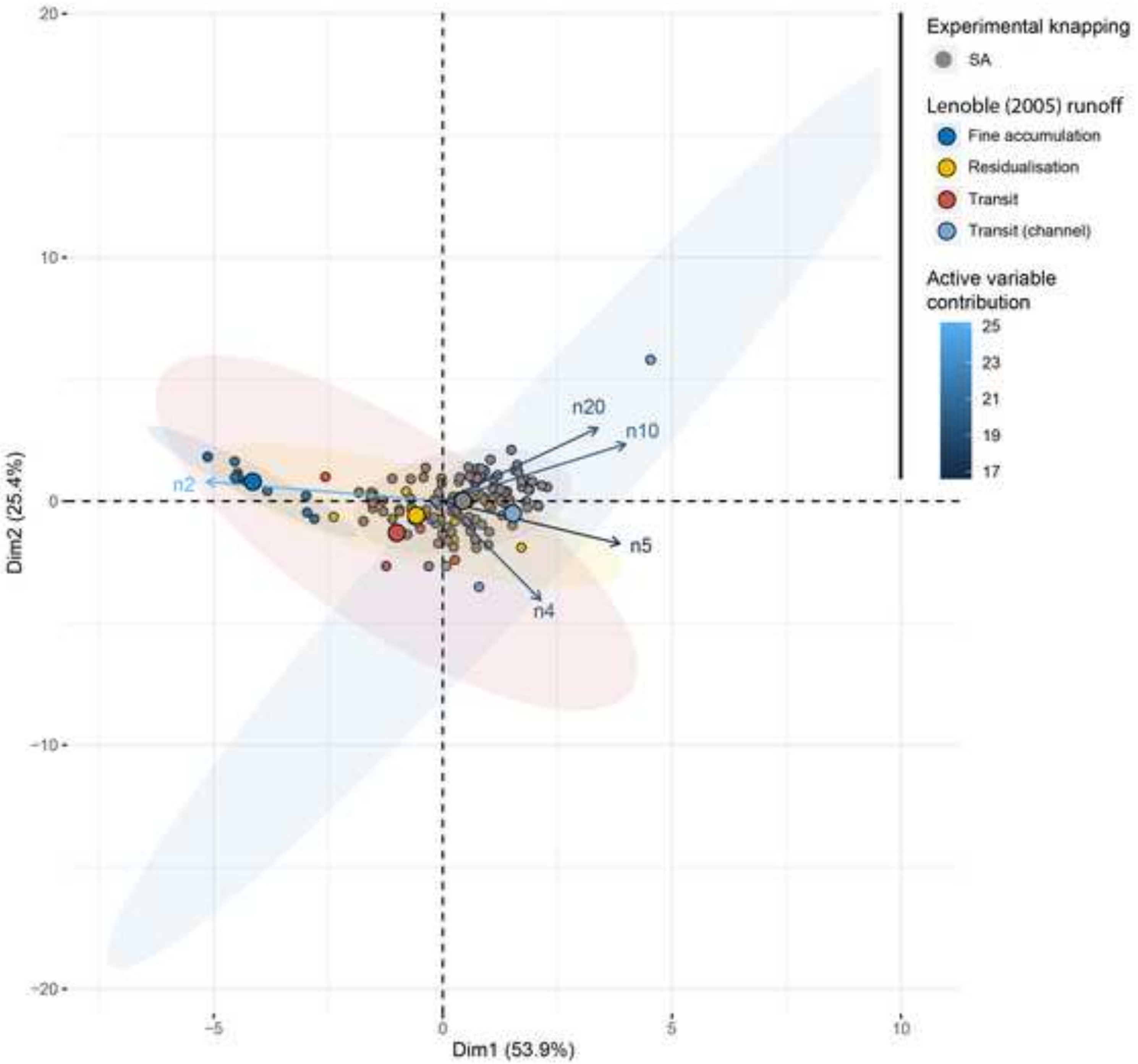

Figure

Click here to access/download;Figure;fig. 14.jpg

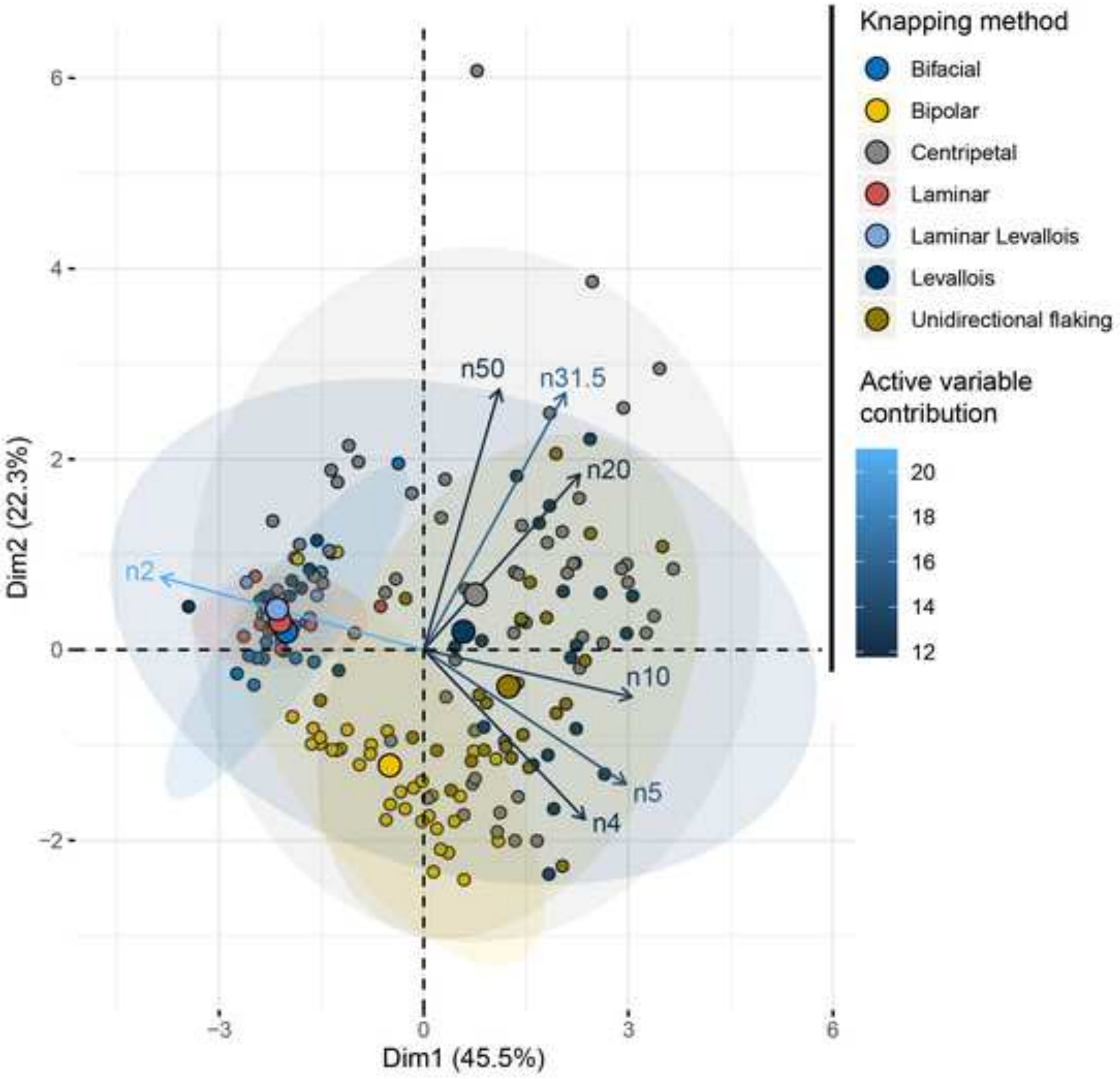

 Click here to access/download;Figure;fig. 1.jpg

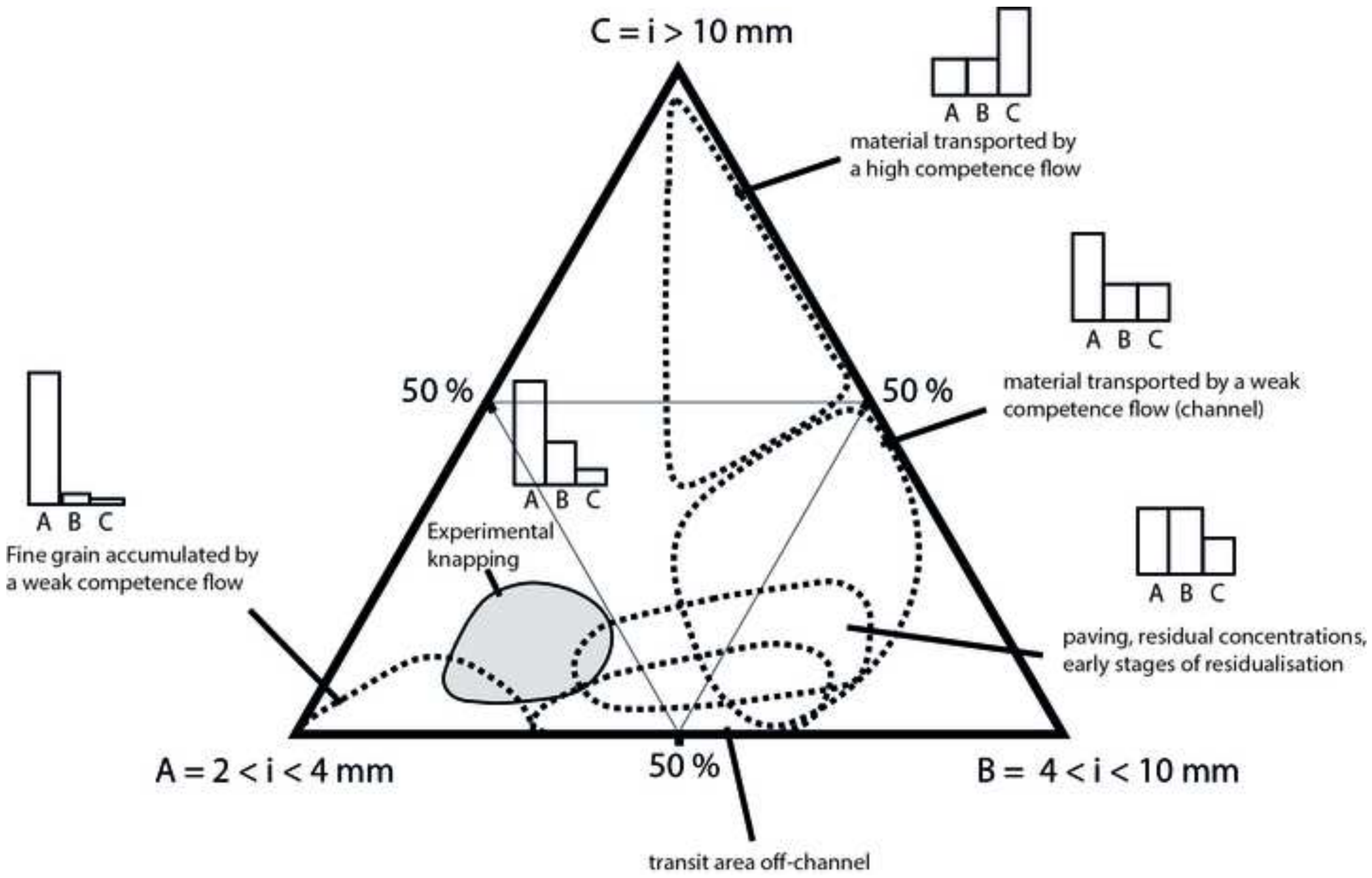

Figure

Click here to access/download;Figure;fig. 6.jpg

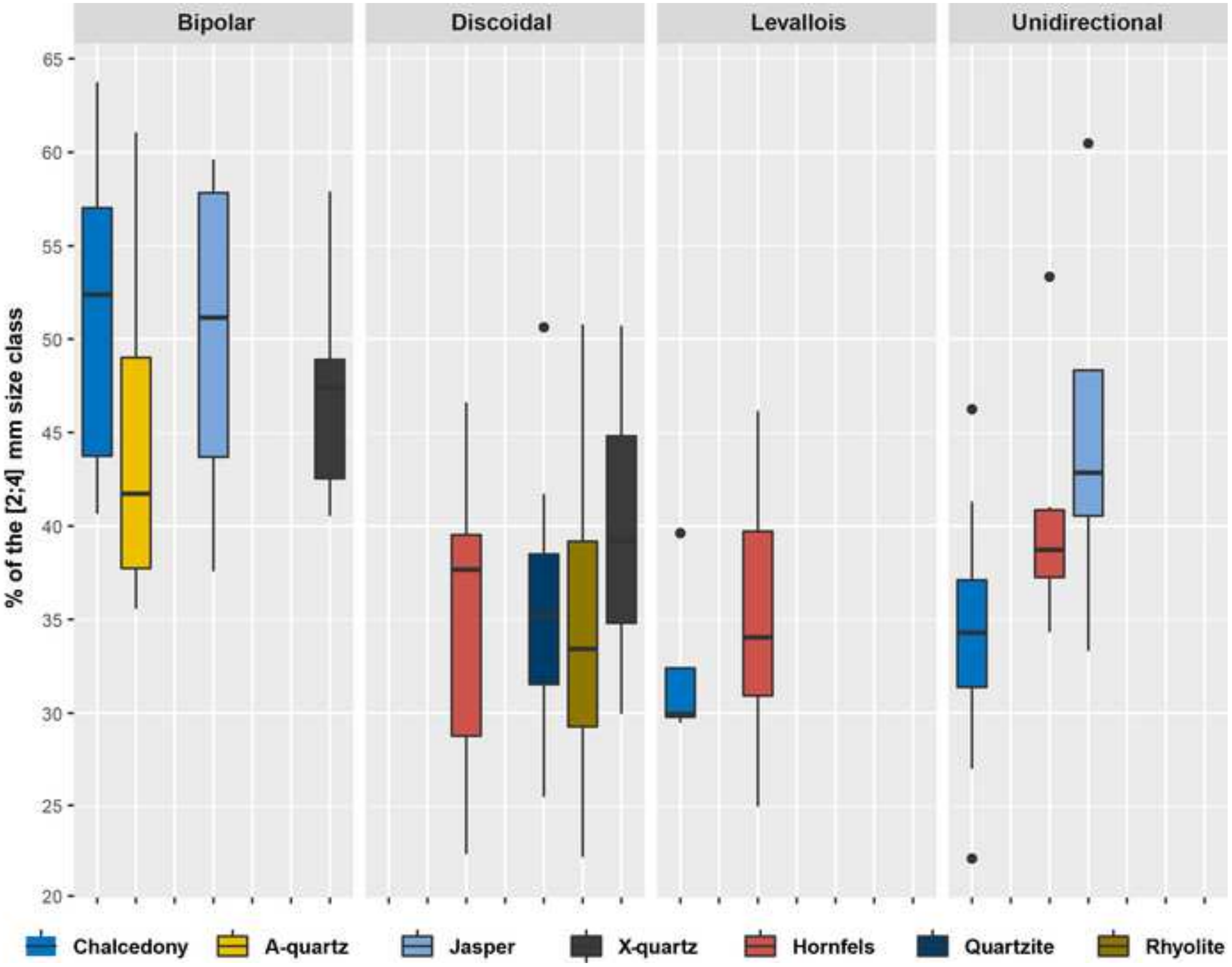

Click here to access/download
Supporting Information
SM1.csv

Click here to access/download
Supporting Information
SM2.pdf

Click here to access/download
Supporting Information
SM3.Rmd

~~Particle size distribution of Ssouthern African rocks and methods of debitage~~

Particle size distribution: an experimental study using southern African reduction methods and raw materials

Formatted: Heading 1, Left, Line spacing: single

Formatted: Font: 14 pt

Formatted: Centered

de la Peña, P.[1,2,3¶*], Thomas, M.[4¶]; Molefyane, T.R.[5]

1 Departamento de Prehistoria y Arqueología. Universidad de Granada, Spain.~~Evolutionary Studies Institute, University of the Witwatersrand, South Africa~~

2 McDonald Institute for Archaeological Research, University of Cambridge, United Kingdom

3 ~~Center of Exploration of the Deep Human Journey, University of the Witwatersrand, South Africa~~Evolutionary Studies Institute, University of the Witwatersrand, South Africa.

4 UMR 5608 TRACES, Université Toulouse Jean Jaurès, Toulouse, France

5 ~~Departamento de Arqueología.~~ Guías de Espeleología y Montaña (GEM), Spain

E-mail: paloma.delapenya@gmail.com (PdlP)

Formatted: Spanish (Spain)

Formatted: English (South Africa)

Abstract

We ~~performed~~ experimentally created a particle size ~~analyses~~ data~~base~~set ~~focusing~~ that is based on reduction sequences and raw materials typical of the Middle and Later Stone Age ~~record of~~ in s~~S~~outhern Africa. The reason for creating this new dataset is that current particle size frameworks are based, almost exclusively, on flint and western European knapping methods. ~~By cCreating a southern Africa focus~~We produced the dataset ~~(with~~using ~~frequent~~ knapping methods and raw materials ~~of this region),~~frequently encountered in the southern African archaeological record because we wanted to test whether ~~-~~it has the same distribution as ~~the if the experimental proposed European~~ particle size datasets experimentally created in Europe, and to initialise the production of a database ~~proposed for Europe reproduce for other experimental examples based in other archaeological scenarios. The long-term goal is creating a robust dataset to~~ for use in the analysis of lithic assemblages from southern African Late Pleistocene deposits. We reduced 117 cores of quartz, quartzite, jasper, chalcedony, hornfels, and rhyolite. The knapping methods selected were unidirectional, discoidal, *Levallois* recurrent and bipolar flaking. ~~, and bipolar flaking, typical of southern Africa contexts in the Late Pleistocene.~~ In this article we compare this new particle size distribution dataset ~~on Southern African raw materials and knapping methods~~ with the results obtained from previous experiments. We found that t~~T~~he s~~S~~outhern African dataset ~~on knapping we have produced~~ shows a wider size range distribution, which seems to be explained by d~~. D~~ifferences ~~among~~ in knapping methods and raw materials~~. seem to explain this variability. Finally, we show an~~ Our results show that there is overlap between the distribution~~s~~ of the s~~S~~outhern African experimental knapping dataset and the sorting experiment~~s~~ ~~carried out~~conducted by

~~A.~~ Lenoble on flint arte~~i~~facts in a runoff context~~s~~. This article shows that a particle size analysis ~~of a lithic assemblage must be combined with a technological analysis to specify its implementation and to properly interpret its results. We also show that~~In addition, the size distribution analysis of lithic ~~material~~ is not sufficient on its own to assess the perturbation of an archaeological assemblage and must be coupled with other analytical tools.

~~the size distribution analyseis of lithic material is not sufficient on its own to assess the perturbation of an archaeological assemblage and must be coupled with other analytical tools.~~

Introduction

Stone Age sites are complex sedimentary accumulations, the formation of which depends on a combination of anthropogenic and natural processes [1–4]. Natural processes, including sedimentary processes, have often altered the spatial organization, composition, and chronological signal of the archaeological record [5]. To measure this loss of information on sites, disturbance assessment studies need to be carried out. Any archaeological remains can undergo the effect of ~~the~~ sedimentary processes, governed by physical laws [4,6]. Thus, particle shape, size and density are the main physical criteria~~on~~ used to measure the degree of perturbation of the archaeological record [2]. The particle size analysis of lithic artefacts was first applied in Africa by K. Schick [2]. She pointed out that in many cases the sites are associated with sedimentary contexts affected by water ~~bodies~~ (alluvial environments, lake margins, basins, marine beaches or even spring deposits). In her seminal work, K. Schick [2] conducted experiments to understand the spatial dispersion and size-class distribution (maximum~~al~~ length of artefacts considered) of lithic industries affected by water flow. She concluded that water flow can substantially, if not radically, affect archaeological assemblages [7,8]. In addition, other natural processes are likely to have an impact on the particle size distribution of lithic assemblages, such as solifluction [9,10], trampling [11–18], ~~or~~ and aeolian processes [19].

Other studies have extended and followed a similar methodology to Schick’s work, applying it~~,~~ mainly~~,~~ to European Palaeolithic contexts, particularly ~~o~~in the ~~S~~southwest of France [4,20,21]. ~~To allow~~In order

for there [illegible]e a systematic approach~~es on~~ to the study of sites, A. Lenoble and P. Bertran ~~set up~~developed a method based on sieving instead of manually measuring all lithic art~~i~~efacts [4,20,21]. Based on the observation that rock knapping consistently cause~~d~~s the same particle size distribution, ~~A. Lenoble and P.~~ Bertran and colleagues [4,20,21] [illegible]posed to compare an experimental knapping assemblage~~s~~ (from [21] called “EU database” for ease of reading in this article) to an experimental one~~s~~ subjected to overland water flow (from [4] called “RUNOFF dataset” for ease of reading in this article) (see supplementary material 1(SM1)). The particle size distribution of these two sets of experiments: “EU dataset” and the “RUNOFF dataset” show separated distributions in a ternary plot~~,~~ (Fig 1). The knapping experiments were done mainly on flint and quartzite and included blade production, *Levallois* and d~~D~~iscoid flaking, and bifacial shaping. The authors also added microlithic reduction sequences inspired ~~of~~ by Pal*a*eo-~~E~~eskimo knapping methods from the ~~E~~eastern Canadian Artic (called “ESKI dataset” for ease of reading in this article, from [22])).

In all knapping experiments, the distribution sizes follow a Weibull ~~function~~ pattern, being long-~~-~~tailed and positively skewed [23] with a proportion of largest pieces tending towards 0 [2,4,20–23], whereas the results of the “RUNOFF dataset” highlight different kinds of distributions according to where one stands in the runoff system. This clear distinction ~~of~~ between both experiments in the final model- allows one to test the size sorting of lithic archaeological assemblages ([20,21], Figure 1). However, this experimental framework mainly includes debitage methods and raw materials typical of European contexts. Thus, the use of both datasets ~~in~~ to study African assemblages is questionable, as ~~other~~ different raw materials and knapping methods were used in Africa in the past.

Formatted: Not Highlight

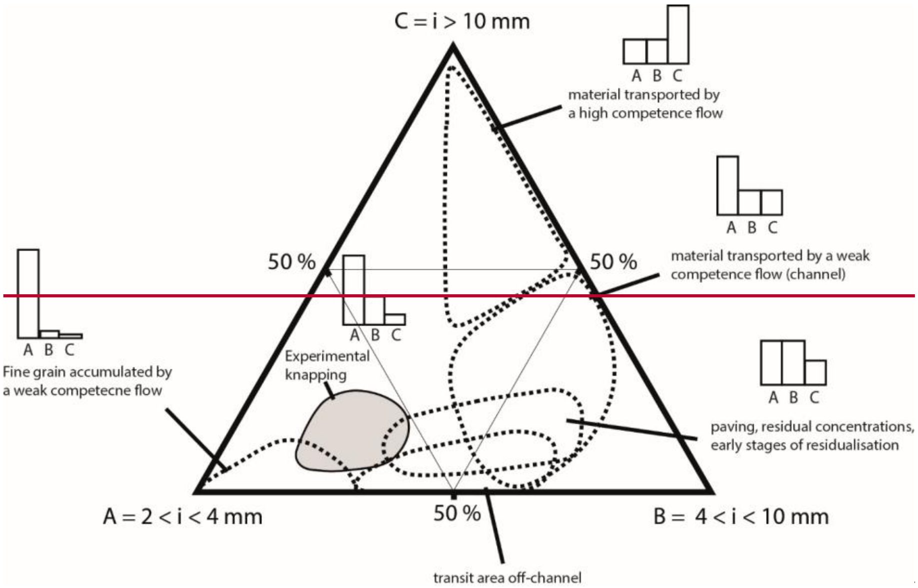

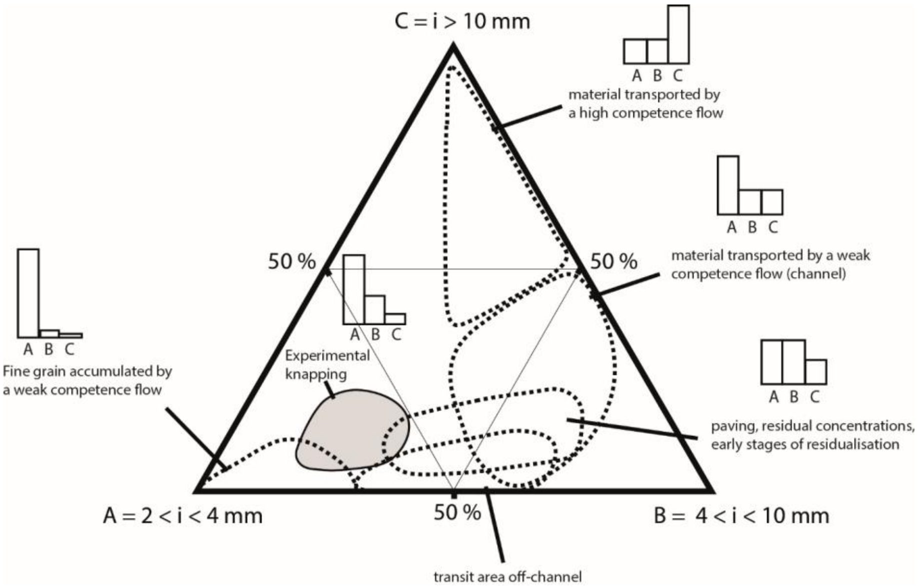

Fig 1: Particle size evolution of a lithic assemblage affected by overland water flow from the experiment of A. Lenoble [4]. The hatched light brown area is the distribution of the EU dataset [20,21]. The dashed areas correspond to the RUNOFF experiments size class distribution in the RUNOFF experiment [4]. The "A" triangle summit corresponds to the [2;4 [illegible] width size class percentage, the "B" triangle summit to the [4;10] mm width size class percentage and "C" to the [10;+∞[mm width size class percentage]. i = ?. From [4].

Formatted: Font: Not Bold

Formatted: Font: Not Bold

Questions and objective

In this paper, we ~~offer~~ contribute experimental research to the field of lithic archaeolog[illegible], focusing~~,~~ strictly~~,~~ on ~~S~~southern African knapping methods and raw materials. The reason for creating this specific experimental dataset was to assess if the previous experimental datasets created in Europe for Middle and Upper Palaeolithic archaeological contexts (such as EU and RUNOFF datasets [4,20,21]), and mainly based on flint (and to a lesser extend ~~also~~ quartzite) [4,20,21], were useful or not for the ~~comprehension~~ understanding of southern African archaeological lithic assemblages. We wanted to test if similar patterns of particle size distribution would be reproduced in southern African assemblages. The reason for this specific enquir~~y~~e is that in southern African late Pleistocene contexts there are different ~~frequency of~~ methods of debitage and raw materials from those used in Europe. We hypothesized that different knapping methods and raw materials would create different proportions of lithic particles. For this purpose, we performed a large experiment on particle size analysis focusing on s~~S~~outhern African rock varieties (automorphic and xenomorphic quartz, quartzite, jasper, chalcedony, hornfels and rhyolite) and knapping methods typical of the Middle and Later Stone Age records (unidirectional, bipolar knapping, discoid, recurrent *Levallois*). The knapping methods selected are ~~quite~~ relatively common in Middle and Later Stone Age assemblages (*vid.* [24,25] *inter alia*). The raw materials are the most common rocks used for knapping in the interior parts of southern Africa for the Late Pleistocene. The ~~sole~~ exceptions to this selection ~~areis maybe~~ are dolerite and silcrete, which ~~that~~ were excluded ~~in~~ from this analysis because ~~. The reasonsreason for this exclusion is that~~ dolerite is an extremely difficult rock to knap~~p~~ and silcrete ~~is a rock which~~ does not occur in any of the sites that we currently study.

This new experimental dataset (called "SA dataset" in this article, see SM1) will be useful for our own research on Middle and Later Stone Age sites and offer a ~~shareable~~ feely available dataset for other archaeologists working o~~i~~n African material. Additionally, this new investigation serves as a control experiment that is comparable to previous European publications on this type of research [20,21]. Moreover, this new dataset will test if different raw materials and ~~new~~ knapping methods, such as bipolar knapping and unidirectional reduction, generate different results ~~to~~ from the previous

Formatted: Not Highlight

experiments ~~in flint for laminar, Discoid and *Levallois* exclusively~~ [20,21]. The new results and the comparison ~~to~~ with previous experiments will ultimately serve to study in depth and without bias the archaeological assemblages ~~of~~ from southern Africa ~~with a similar set of raw materials and debitage as the ones presented here~~. ~~Thus, the ultimate~~Our immediate goal is to compare the results of this experimental research ~~for~~ with archaeological lithic assemblages from sites such as Mwulu's Cave, Border Cave ~~or~~ and Marshill (studied by one of the authors, PdlP). ~~The~~ Our research questions are:

(1) What is the particle size distribution ~~of~~ specific to s~~S~~outhern African raw materials and knapping methods?

(2) Are there statistically significant differences regarding raw materials?

(3) Are there statistically significant differences regarding the knapping methods?

(4) Is the particle size distribution of the SA dataset different ~~to~~ from the EU dataset?

(5) Is the particle size distribution of the SA dataset different ~~to~~ from the RUNOFF dataset?

The null hypotheses are that there is no statistical difference between~~, on one hand,~~ the different raw material and knapping method datasets compared in this study, and~~, on the other hand, with~~ compared with previous published datasets [4,20,21].

Material and methods

One of the authors (PdlP) ~~did~~ conducted all of the experimental knapping~~s~~, having several years of stone tool making ~~competence~~experience. The experiment includes the most common s~~S~~outhern Africa rocks and lithic reductions used during the Middle and the Later Stone Age (*vid. supra)*. They are summarized in Table 1 with the number of cores reduced for each one of the knapping methods and raw material. In Fig 2, we show schematically all the knapping methods deployed in this analysis. In total 117 cores

were knapped for this experiment (see SM1). In Fig 3 the main raw materials are illustrated, alongside a selection of tools made during the experiments.

Formatted: Not Highlight

Table 1: Synthesis of the knapping methods and raw materials used in this experiment (A-quartz = Automorphic quartz, X-quartz = Xenomorphic quartz)

	Chalcedony	A-quartz	Hornfels	Jasper	Quartzite	Rhyolite	X-quartz	Total
Bipolar	8	4		10			10	32
Discoidal			10		10	10	12	42
Levallois	4		16					20
Unidirectional	10		8	5				23
Total	22	4	34	15	10	10	22	117

Formatted: Font: Italic

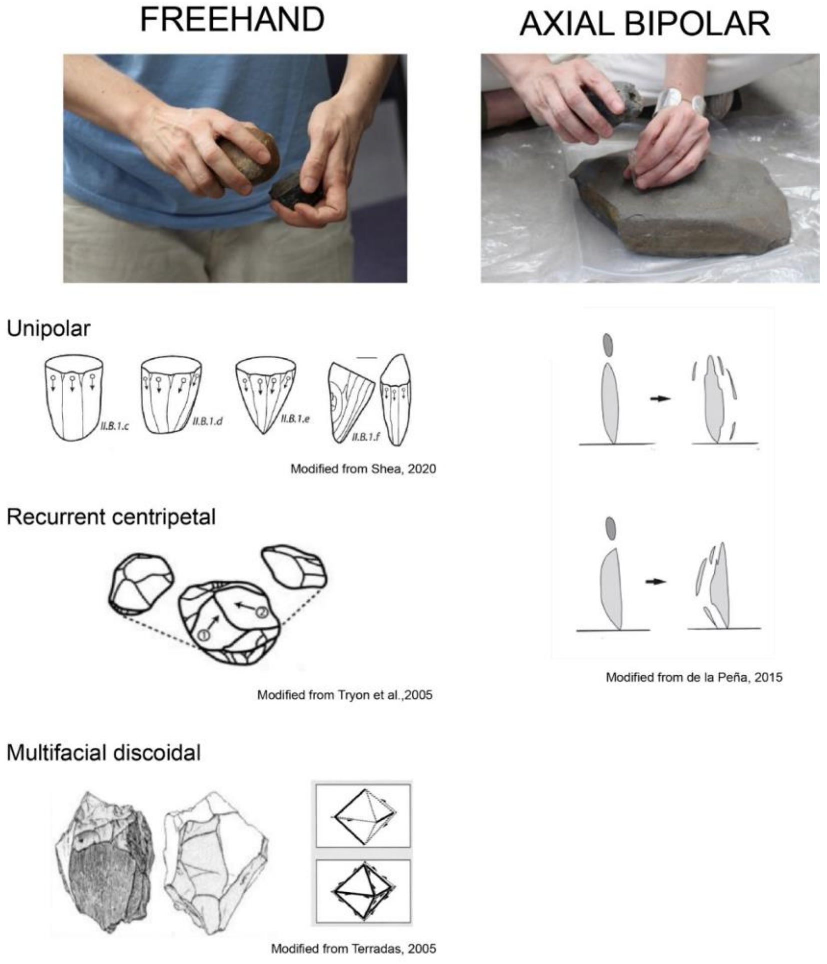

Fig 2: Examples of Middle and Later Stone Age reduction~~s~~ sequences ~~done~~ employed in this knapping experiment: discoidal, *Levallois* recurrent, unidirectional, and axial bipolar ~~knapping~~. The figures are modified from [15, 40, 48, 53]. On the left freehand variants and on the right bipolar axial variant towards miniaturization. Photos: Paloma de la Peña

Formatted: Font: Italic

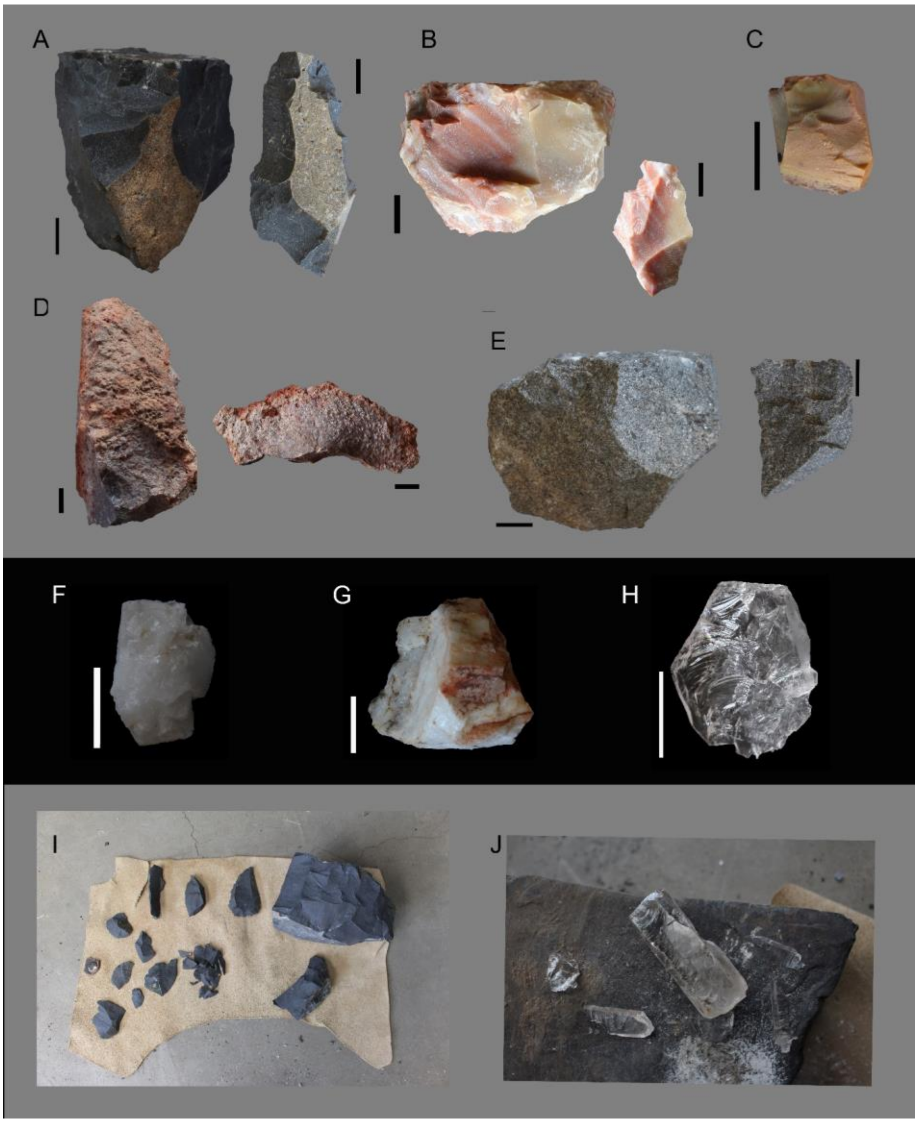

Fig 3: Raw materials used for this experiment. A. Hornfels (u~~U~~nidirectional core and flake). B. Jasper (u~~U~~nidirectional core and flake). C. Chalcedony (chunk). D. Rhyolite (core fragment) and flake (ventral view). E. Quartzite (d~~D~~iscoidal core and flake, dorsal view). F. Xenomorphic quartz (b~~B~~ipolar core). G. Xenomorphic quartz (core fragment). H. Automorphic quartz (b~~B~~ipolar core). I. Selection of blanks and cores in hornfels during one of the u~~U~~nidirectional reduction sequences. J. Core and blanks in the middle of the reduction through b~~B~~ipolar axial knapping of one of the automorphic quartz cores. All scales are 1 cm except for H, which is 5 mm. All the material presented is from this~~e~~ experiment~~s~~, except for C and H ~~rocks~~, which were previously produced. Photos: Paloma de la Peña.

During the experiments, we tried to reduce the core until exhaustion, in other words, until no more flakes could be extracted. For the knapping technique in this experiment, we used a hard mineral percussion with a banded iron pebble from Kwa~~-~~Zulu-~~-~~Natal and a soft mineral hammer, a sandstone pebble from the Eastern Cape.

The specific knapping methods selected are as follows:

- Bipolar knapping: ~~(i.e., knapping on an anvil) is a~~A method in which the core is placed on an anvil and held with the bare hand. The rock is ~~hit~~ struck from above with a hammer held in the other hand, causing blanks to fly off from the top and from the edge that is in direct contact with the anvil [26,27]. Bipolar knapping has been reported in the Middle Stone Age and Later Stone Age [25].
- Discoidal Knapping: In this experiment we used what [28[illegible]ned multifacial discoidal knapping (called "d~~D~~iscoidal knapping"), ~~but~~ which is equivalent to "c~~C~~entripetal knapping" according to ~~V. Mourre~~ [29]. In this sub-variant the knapper searches for 45 degree~~s~~ angles to obtain flakes until the core is exhausted, changing the striking platform almost every time and using the ~~previous~~ removals as such.
- Unidirectional knapping: In this method, we tried to exploit~~,~~ as much as possible~~,~~ from one striking platform ~~and~~ following a unidirectional sequence of removals. Once the knapping surface from the first striking platform wa~~i~~s exhausted, we rotated the core and continued knapping~~,~~ from a different striking platform, following the same strategy if possible.
- *Levallois* recurrent (also referred to in the African literature as 'prepared cores'): In our experiments we followed the guidelines explained by E. Boëda [30]. Sometimes during reduction, we could extract two series of *Levallois* flakes. In some cases, we finished the knapping of these cores using discoidal reduction. This was done to obtain the most out of the volume of the rock in the final stage, and only when there was no possibility to prepare two hierarchical knapping surfaces in a *Levallois* fashion.

The raw materials used for the experiments (Fig 3) ~~are~~include:

- Hornfels, ~~is~~ a fine- to medium-~~-~~grained metamorphic rock that is formed by contact metamorphism on the margins of igneous intrusions that crosscut the country rock [31]. The two varieties of hornfels used for this study come from two different dolerite dykes that intruded ~~on~~ shales, one in the Eastern Cape and another ~~one~~ in the Northern Cape. The variety used from the Eastern Cape was obtained from a river cutting of a massive hornfels contact metamorphism layer. ~~Big~~ Large chunks of the material were ~~obtained~~ extracted and broken into smaller blocks for the experiments. The hornfels ~~of~~ from the Northern Cape came in small slabs, usually of less than 10 cm for their longest axis.
- Chalcedony, ~~is~~ a cryptocrystalline quartz [31]. The one we used is not banded, and in South Africa it mostly occurs in white, gray, black, or blue shades. Chalcedonies fill cavities and line fissures in rocks. In the Drakensberg and Lebombo Group, they occur in both basalts and rhyolites. Sometimes chalcedony contains a monoclinic polymorph called moganite [31]. The chalcedony of this experiment comes entirely from Siberia Farm, near a r~~R~~ock a~~A~~rt site also known in the area as Franshoek. It is an outcrop with varieties of rocks ranging in colour from green to orange.
- Jasper, ~~is~~ a chert coloured by the presence of iron [31]. The variety used for this experiment was banded red jasper from the Eastern Cape.
- Automorphic quartz (c~~C~~rystal quartz, called A-quartz in this article, following [32]), ~~is~~ a euhedral mineral with the shape of a single large~~big~~ hexagonal ~~single~~ crystal terminating~~ed~~ alternately in~~by~~ major and minor rhombohedral faces [33]. Its flat faces are well developed around a symmetrical axis and bounded by rectilinear ridges [34,35]. The crystal quartz used for this experiment comes from a small vein in Limpopo Province, from the surroundings of Mwulu's Cave [36], specifically from the Black Reef F~~f~~ormation.

- Xenomorphic quartz (following [32], called X-quartz in this article), ~~is~~ a solid polycrystalline aggregate with an anhedral texture formed by a close-packed mass of crystals that are not usually bounded by their own crystal faces, but have their outlines ~~im~~pressed on them by the adjacent crystals [35,37]. All the xenomorphic quartz used for this experiment comes from the ~~surroundings of the~~ Cradle of Humankind (Gauteng) area, near ~~the famous~~ the site of Swartkrans ~~Cave~~.
- Quartzite, ~~is~~ a metamorphosed sandstone. All the Quartzite used in this experiment comes from the Black Reef F~~f~~ormation in the surroundings of Mwulu's Cave [36] in Limpopo Province.
- Rhyolite, ~~is~~ an extrusive igneous rock with a high silica content. Rhyolite is made up of quartz, plagioclase, and sanidine, with minor amounts of hornblende and biotite. Trapped gases often produce vugs in the rock. These often contain crystals, opal, or glassy material. The rhyolite used for this experiment comes from the immediate surroundings of Border Cave in the Lebombo mountains (Kwa~~-~~Zulu-~~-~~Natal, South Africa).

To ~~allow~~ enable comparison ~~with this~~of the s~~S~~outhern African experimental knapping dataset with the RUNOFF dataset [4] and the EU dataset [20,21] we have used~~keep~~ the same size classes. The previous experiment~~y~~ used mesh~~es~~ of 2, 4, 5, 10, 20, 31.5 and 50 mm. After gathering all the particles produced during the knapping process, w~~W~~e hand-~~-~~sieved the ~~experiments~~ material in the same way recommended ~~in~~ by Bertran et al. [20,21]. ~~after gathering all the particles produced during the knapping process.~~ As particle size analysis is time consuming, to speed up the counting for material-rich assemblages, the weights of small particles (2 and 4 mm) were obtained using mass/count ratios. We thus counted all the particles per size class and weighed them.

To describe our dataset and ~~to~~ compare it with previous data we used ternary plots made with Past3. [38]. Additional statistics were made ~~thanks to~~using the method developed by Weaver et al. [39] to obtain 95% confidence intervals in ternary plots (Fig 4). To compare the different datasets, we use Principal Component Analysis (PCA) performed under R using the FactoMineR package [40]. The

MANOVA tests ~~have been done on~~were run using Past3. The statistical descriptions of the PCA analysis are available in SM2 and SM3. Raw material and reduction strategies choices ~~has been realized to stick~~were made to follow as ~~much~~ closely as possible ~~to~~ the ~~s~~Southern African archaeological context (*vid. supra*). All knapping methods are not applied to all raw materials, as for example there are certain knapping methods that never appear ~~in~~ on certain raw materials in the southern African archaeological record. For instance, to the best of our knowledge, there is no *Levallois* reduction ~~for~~ on automorphic or xenomorphic~~-~~ quartz in archaeological assemblages, and in the same vein ~~that~~ there is no record~~s~~ of bipolar knapping on anvil for hornfels~~ in archaeological sites. Furthermore,~~ T~~t~~he number of experiments for each one of the variants explored per raw material is not consistent, as in some cases during the experiment~~ation~~ the bad quality of the raw material (because of flaws and natural fractures) did not allow us to complete a reduction sequence. We tried ~~at least~~ to have at least five examples for each one of the raw materials and debitage variants and, ideally, to get 10 examples~~ at least~~. This was not possible for all the variants selected (Table 1) because of the reasons explained. Thus, we assume statistical bias in the comparisons we performed as each raw material has~~ve~~ not been knapped through each knapping method. Thus~~en~~ it is not possible to measure the exact effect of these two last variables on particle size distribution. The aim being to compare archaeologically compatible options.

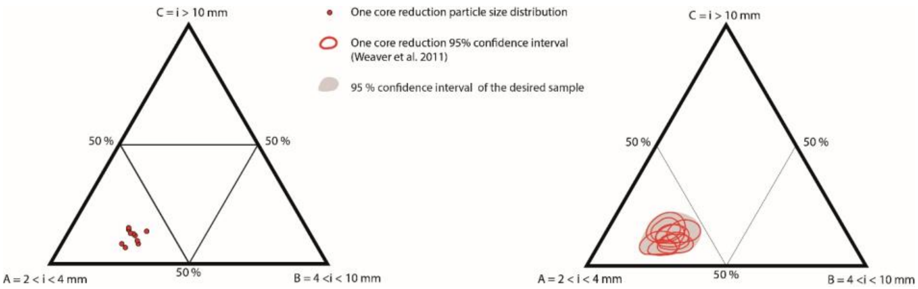

Fig 4: Illustration of how we applied the Weaver et al. (2011) method to create 95% confidence ellipse~~s~~ intervals in the ternary plots.

Results

What is the particle size distribution ~~of S~~resulting from southern African raw materials and knapping methods?

In ~~the -~~SM1 we present the particle size composition of the SA dataset, describing the size classes and their weights~~ counterpart~~. ~~As particle size analysis is time consuming, to speed up the counting for material rich assemblages, weights of small particles (2 and 4 mm) are useful to obtain mass/counts ratios. We counted all the particle per size class and weighed them.~~ ~~In this article we use only counts in the statistics and in the figures.~~ The histogram of the percentages per size class,~~es~~ considering knapping methods and raw materials, clearly shows that for all the raw materials and knapping methods there is a gradual decrease ~~of~~ in particle numbers, as size classes increase. In all the series, the 5 mm particle class is slightly more abundant than the 4 mm size class (Fig 5).

In all of the experiments, ~~either~~ whether considering raw material or knapping method, the size classes 2 to 4 mm constitute~~s~~ around 40-60% of the particles (Fig 6).

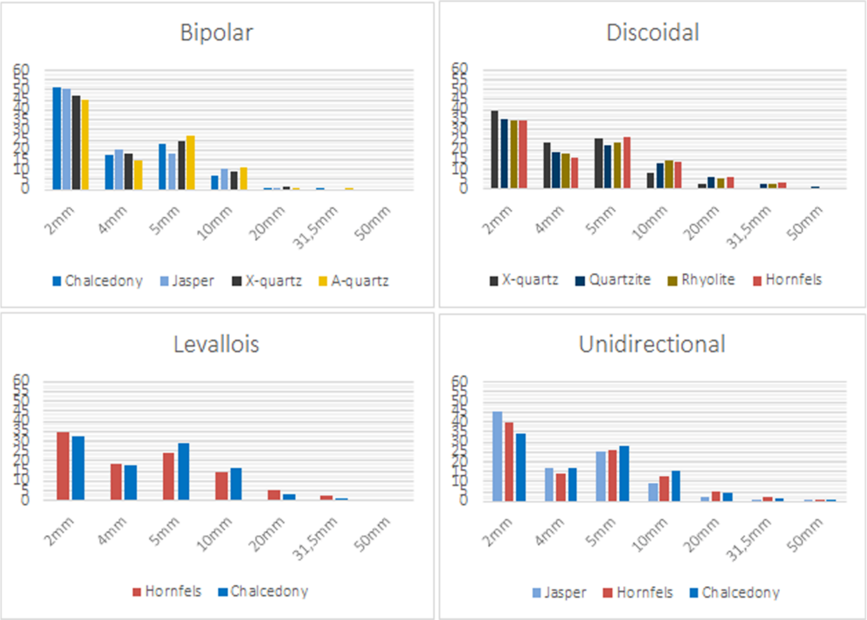

Fig 5: Histograms comparing mean distributions of percentage of particles per size class (counts).

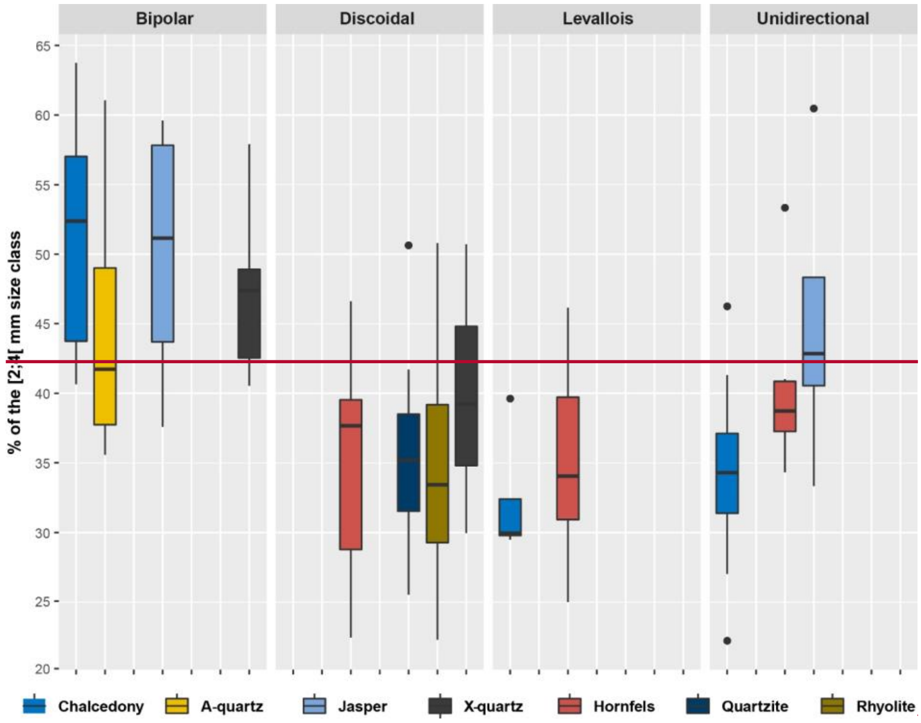
Bipolar
Discoidal
Levallois
Unidirectional
% of the [2;4[mm size class
65
60
55
50
45
40
35
30
25
20
Chalcedony
A-quartz
Jasper
X-quartz
Hornfels
Quartzite
Rhyolite

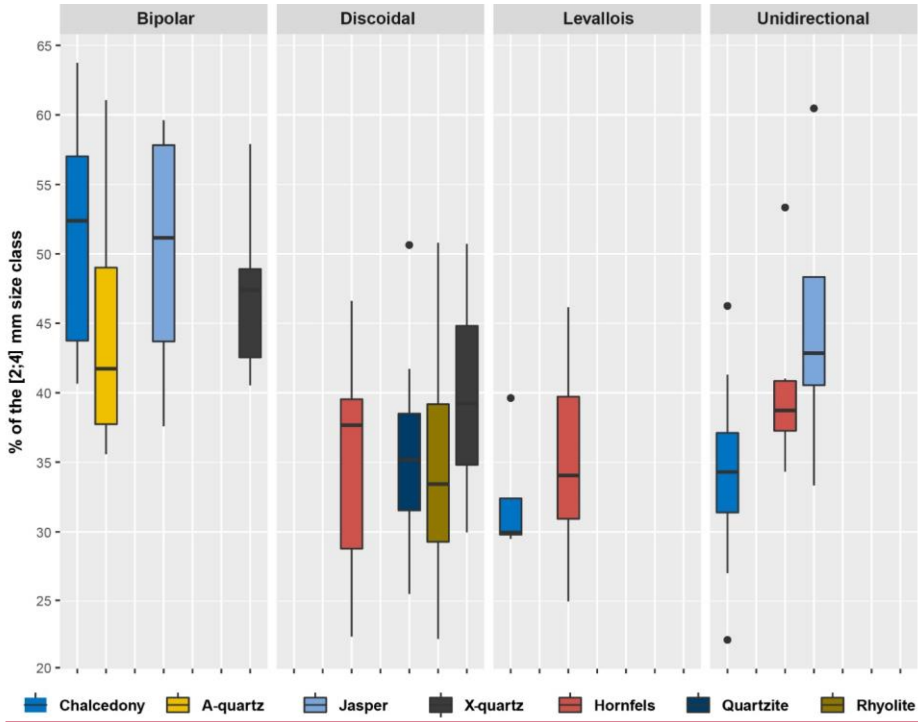

Fig 6: Box plots comparing the proportion of the [2:4] mm size class (counts) depending on the raw material and the knapping method.

The ternary diagrams (Fig 7) show a broad distribution of the SA dataset on experimental debitage size class distribution, ~~which are~~ predominantly composed of particles between 2 and 4 mm and between 4 and 10 mm. The 95% confidence interval calculated for each debitage group considerably increases the range of distribution for each raw material and knapping method.

In the ternary plots (Fig 8) rhyolite, quartzite and hornfels have a similar distribution towards the centre~~r~~ of the charts. Regarding knapping methods, unidirectional, discoidal, and *Levallois* seem to partially overlap~~ partially~~, ~~independently~~ irrespective of the raw material considered (Fig 9). Furthermore, bipolar ~~seems to~~ knapping produces the smallest fractions (between 2 and 4 mm) ~~independently~~ irrespective of the raw material. Freehand knapping (unidirectional, discoidal and *Levallois*) and bipolar knapping distribute~~s~~ slightly separately in the ternary plot.

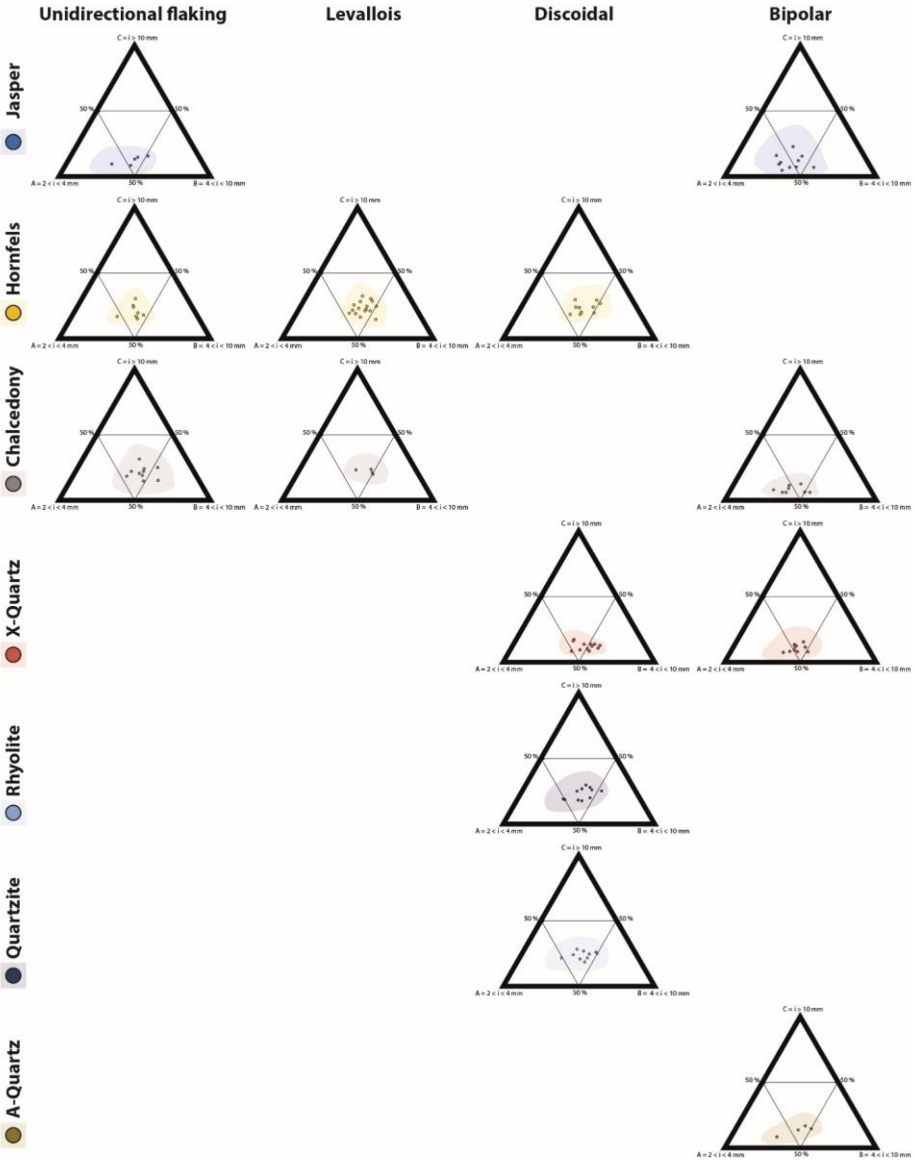

Fig 7: Ternary plots per raw material and knapping methods ~~of~~ in the SA dataset (counts). Shaded areas are 95% confidence intervals.

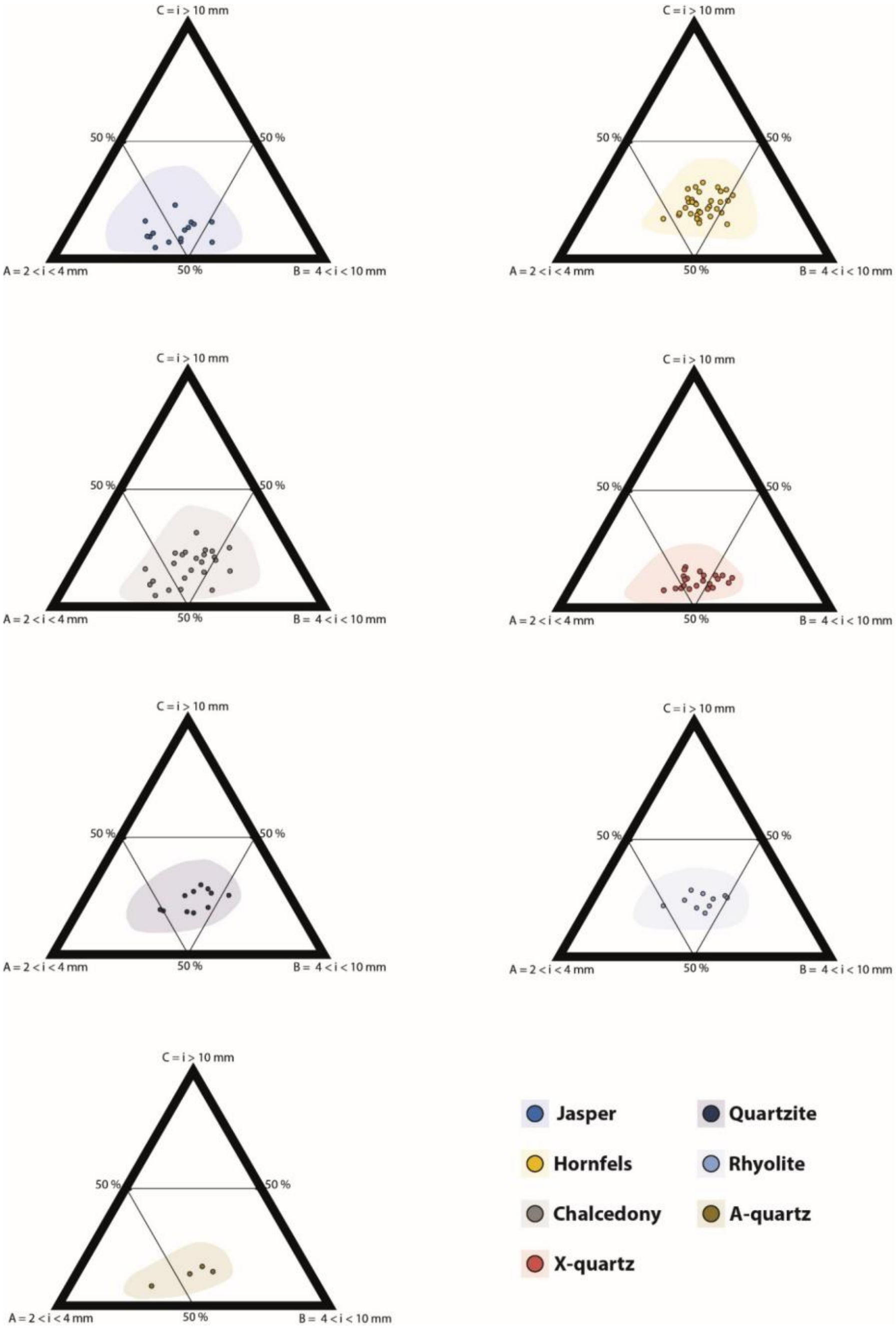

Fig 8: Ternary plots of the particle size distribution per raw material (counts) in the~~of~~ SA dataset ~~per raw material (counts)~~. Shaded areas are 95% confidence intervals.

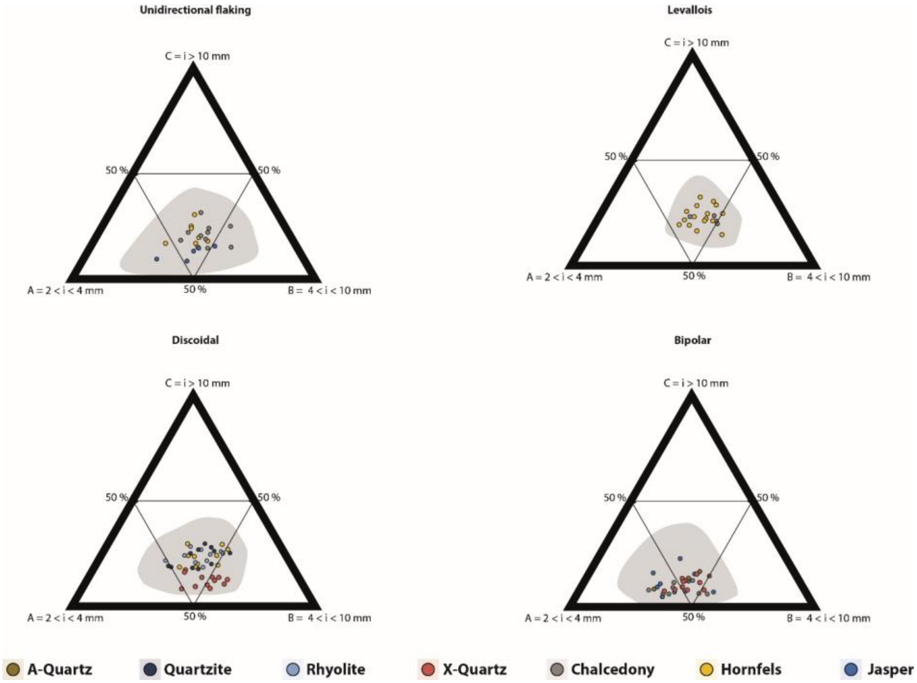

Fig 9: Ternary plots of the particle size distribution per knapping method (counts) in ~~of~~ the SA dataset. ~~per knapping method (counts).~~ Shaded areas are 95% confidence intervals.

Are there statistically significant differences ~~regarding~~ between the raw materials? ~~compare~~

The factorial space of the "Raw material" principal component analysis (Fig 10 and SM2) shows a clear opposition (mostly first axis, 39%) between, on one hand, chalcedony, jasper, X-quartz and A-quartz, and, on the other hand, hornfels, quartzite and rhyolite. The latter produced~~ing~~ larger pieces, but have not been reduced through the bipolar technique. These differences are confirmed by a MANOVA test (using a pairwise comparison) with the raw material as an independent variable and the size distributions as dependent variables (Table 2). A group composed of chalcedony, jasper and x-quartz differs ~~to~~ from hornfels, quartzite and rhyolite.

Table 2. Results of the pairwise comparison from the MANOVA test. Raw material is the independent variable, while s~~S~~ize classes are the depende~~a~~nt variables.

	Chalcedony	A-Quartz	Jasper	X-quartz	Hornfels	Quartzite	Rhyolite
Chalcedony		18,09	0,16	1,51	*0,00*	*0,00*	*0,02*
A-Quartz	18,09		11,61	12,17	0,14	1,22	3,50
Jasper	0,16	11,61		9,63	*0,00*	*0,00*	*0,01*
X-quartz	1,51	12,17	9,63		*0,00*	*0,00*	*0,00*
Hornfels	*0,00*	0,14	*0,00*	*0,00*		*0,01*	13,83
Quartzite	*0,00*	1,22	*0,00*	*0,00*	*0,01*		9,40
Rhyolite	*0,02*	3,50	*0,01*	*0,00*	13,83	9,40	

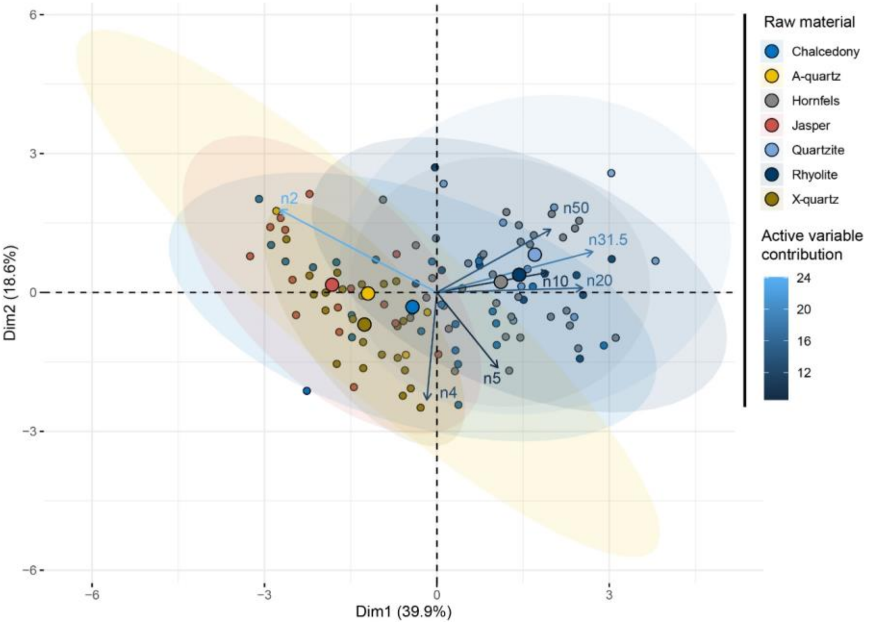

Fig 10: PCA of the SA dataset with the raw material as a supplementary variable.

Are there statistically significant differences ~~regarding~~ between the knapping methods?

The factorial space of the "Knapping method" principal component analysis (Fig 11 and SM2) shows a clear opposition on the first dimension (39% of the variability) between ~~industries~~ methods producing smaller particles (2 to 4 and 4 to 5 mm width size class) and ~~industries~~ those producing larger particles (over 10 mm wide). Bipolar knapping is best described by the size range variables 2 to 4 mm and 4 to 5 mm, ~~and~~ as ~~are~~ opposed to *Levallois*, unidirectional and discoidal methods, the distribution of which seem~~s~~ similar and produce~~s~~ larger ~~remains~~pieces. These differences are confirmed by a MANOVA test with "~~Kanpping~~Knapping method" as an independent variable (Table 3). Bipolar differs ~~to~~ from all the other knapping methods, which have a size distribution that tends to be the same.

Table 3. Results of the pairwise comparison from the MANOVA test. Knapping method is the independent variable, while s~~S~~ize classes are the depende~~a~~nt variables.

	bipolar	discoidal	Levallois	unidirectional
bipolar		*0,00*	*0,00*	*0,00*
discoidal	*0,00*		2,48	0,11
Levallois	*0,00*	2,48		2,41
unidirectional	*0,00*	0,11	2,41	

When combining knapping methods and raw material (Fig 12 and SM2), three groups appear i~~o~~n the factorial space. The first one is best described by small particles (2 to 4 mm), and is composed ~~by~~ of X-quartz d~~D~~iscoidal knapping, all b~~B~~ipolar on anvil, and all jasper knapping experiments. The second group is best described by larger particles. It is composed of all hornfels, quartzite and rhyolite raw materials regardless of the associated knapping method. A third group, composed of chalcedony associated ~~to~~ with *Levallois* and unidirectional flaking, is described ~~by~~ as intermediate width particles (5 to 10 mm size class).

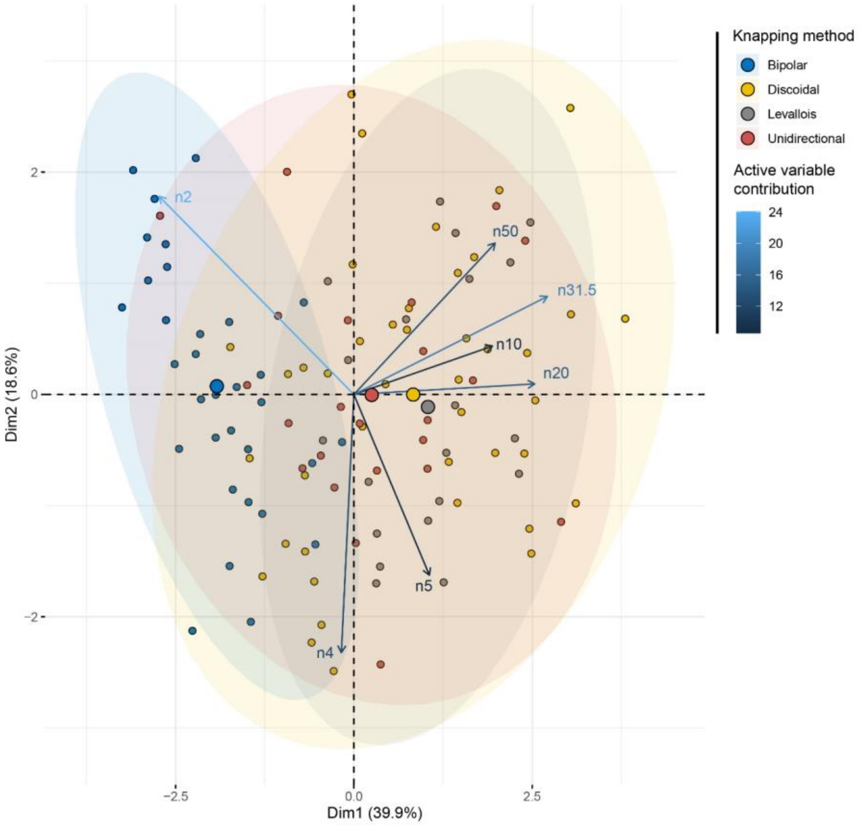

Formatted: Font: 22 pt

Fig 11: PCA of the SA dataset with the knapping method as a supplementary variable.

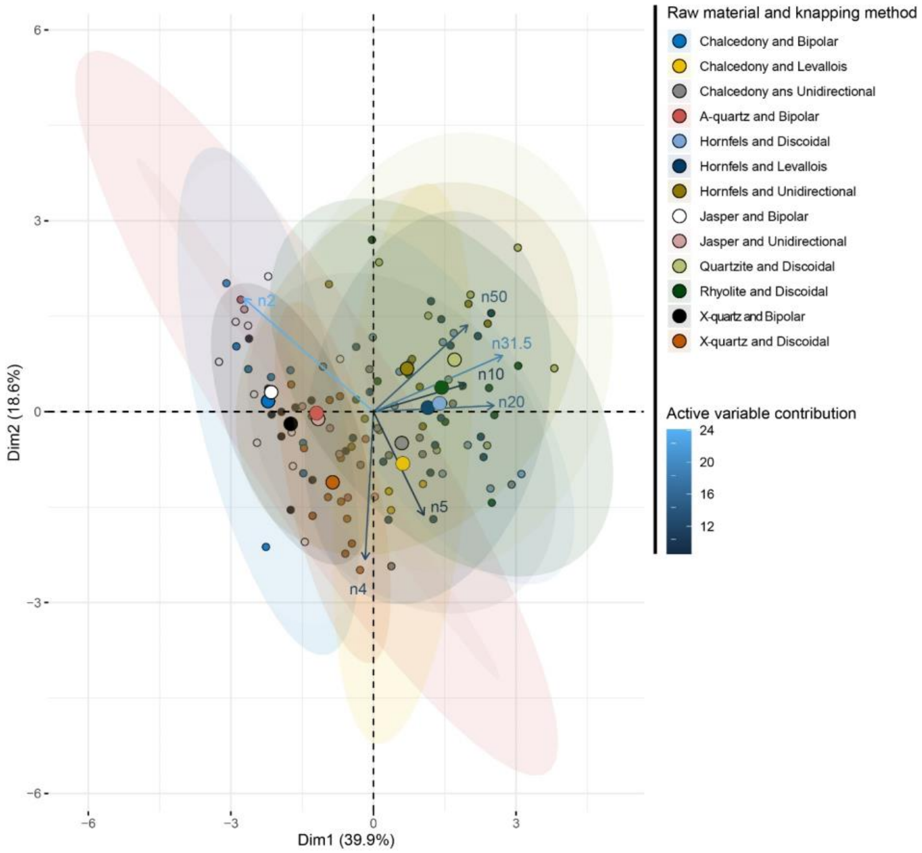

Fig 12: PCA combining knapping and raw material as a supplementary variable (SA dataset).

Is the particle size distribution of the SA dataset different ~~to~~ from the EU dataset?

~~O~~In the ternary diagram we see great differences between the three experimental knapping datasets on particle size distribution~~s~~ (Fig 13). This variability is confirmed by the principal component analysis including the EU dataset on particle size distribution ~~from the European archaeological context~~ [20,21] and the SA dataset (Figs 14 and 15). When considering the knapping methods (Fig 14 and SM2), three main groups appear ~~o~~in the factorial space. The first one is correlated ~~to~~ with small particles (i.e., 2 to 4

mm) and is composed of ~~B~~bifacial shaping and ~~L~~laminar knapping systems. A second group, best described ~~by~~ as intermediate particle sizes, is composed of ~~B~~bipolar blank production. The third group is best described ~~by~~ as having larger particle sizes and is composed of the flaking systems (d~~D~~iscoidal, *Levallois* and u~~U~~nidirectional ~~flaking~~).

When considering raw materials as a supplementary variable (Fig 15 and SM2), flint is opposed i~~o~~n the factorial space to A-quartz, X-quartz, jasper, and chalcedony, which are all opposed to a group composed of rhyolite, quartzite and hornfels. These three clusters are best described respectively by their small particle sizes (2 to 4 mm), intermediate sizes (4 to 10 mm) and larger sizes (20 to 50 mm).

When adding the ESKI dataset in the principal component analysis with the data divided according to type of reduction, such as “knapping”, “shaping” and “retouching” (Fig 16 and SM2), we see a clear opposition i~~o~~n the factorial space between “retouching”, producing smaller remains, and “knapping” producing larger remains. “Shaping” falls between the~~se~~ two.

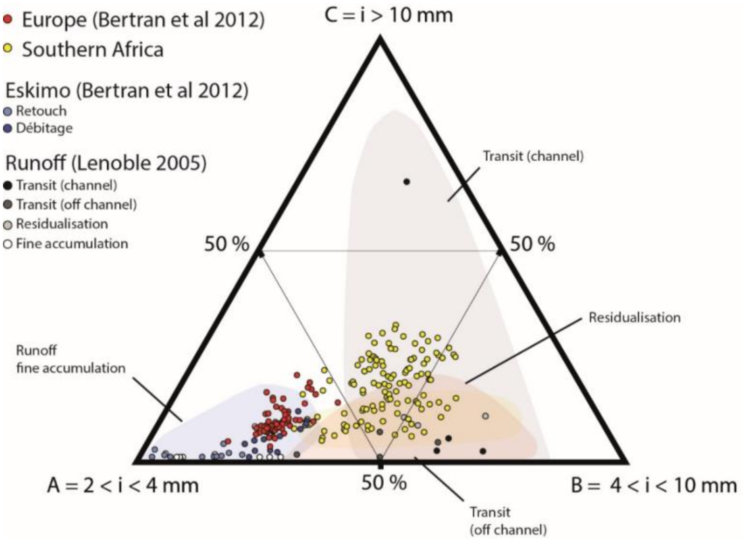

Fig 13: Ternary plots comparing the SA dataset presented in this paper, the EU and the ESKI datasets published in Bertran et al. [20,21] and the RUNOFF dataset [4]. The ternary plots contain 95% confidence intervals.

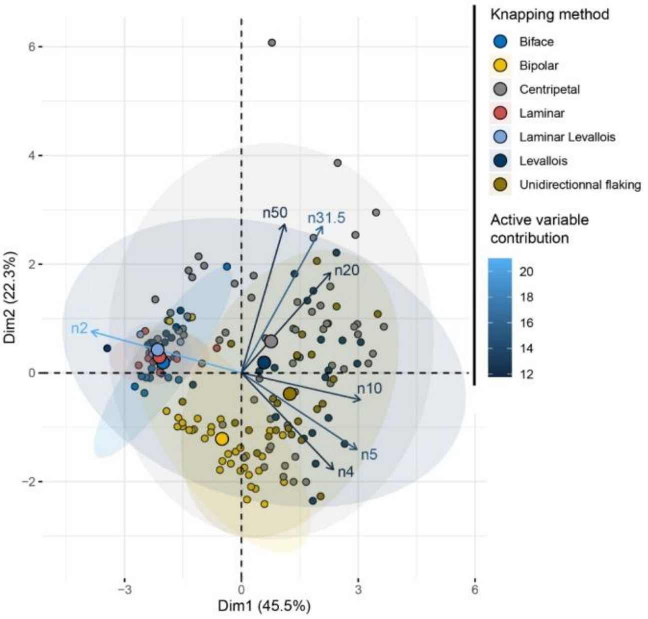
Knapping method
Biface
Bipolar
Centripetal
Laminar
Laminar Levallois
Levallois
Unidirectionnal flaking
Active variable contribution
20
18
16
14
12
n50
n31.5
n20
n2
n10
n5
n4
Dim2 (22.3%)
Dim1 (45.5%)
6
4
2
0
−2
−3
0
3
6

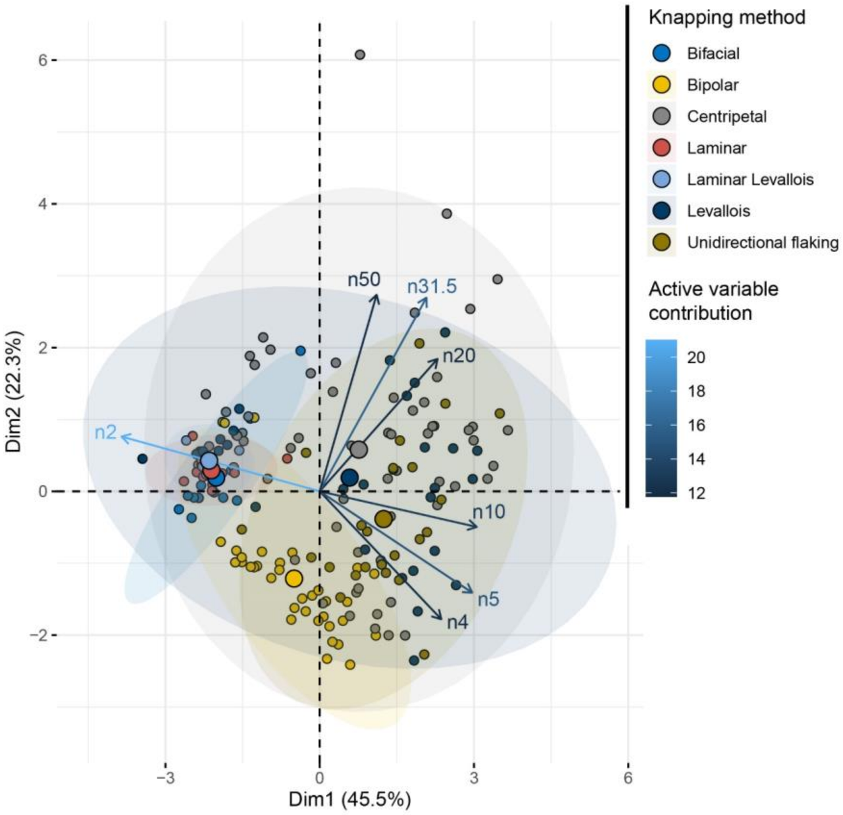
Knapping method
Bifacial
Bipolar
Centripetal
Laminar
Laminar Levallois
Levallois
Unidirectional flaking
Active variable contribution
20
18
16
14
12
n50
n31.5
n20
n2
n10
n5
n4
Dim2 (22.3%)
Dim1 (45.5%)
6
4
2
0
−2
−3
0
3
6

Fig 14: PCA of the SA dataset compared to the EU dataset [20,21]. The PCA has been made considering the knapping method as a variable.

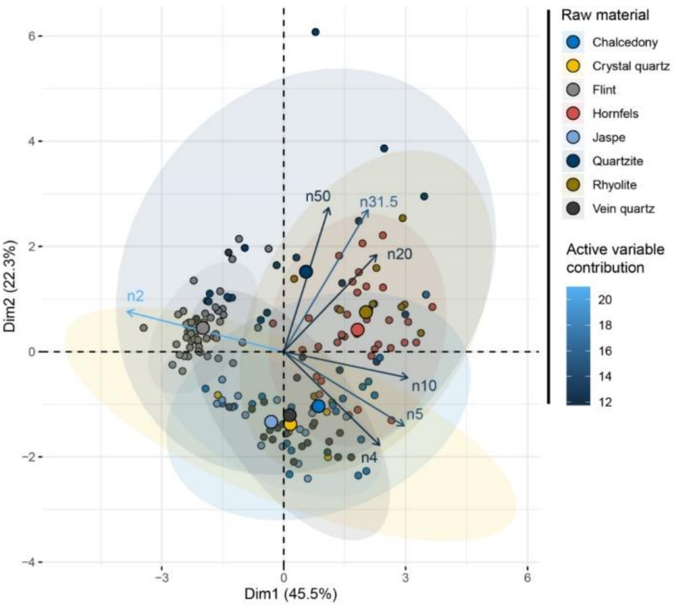

Fig 15: PCA of the SA dataset and the EU dataset [20,21], having 'raw material' as a supplementary variable.

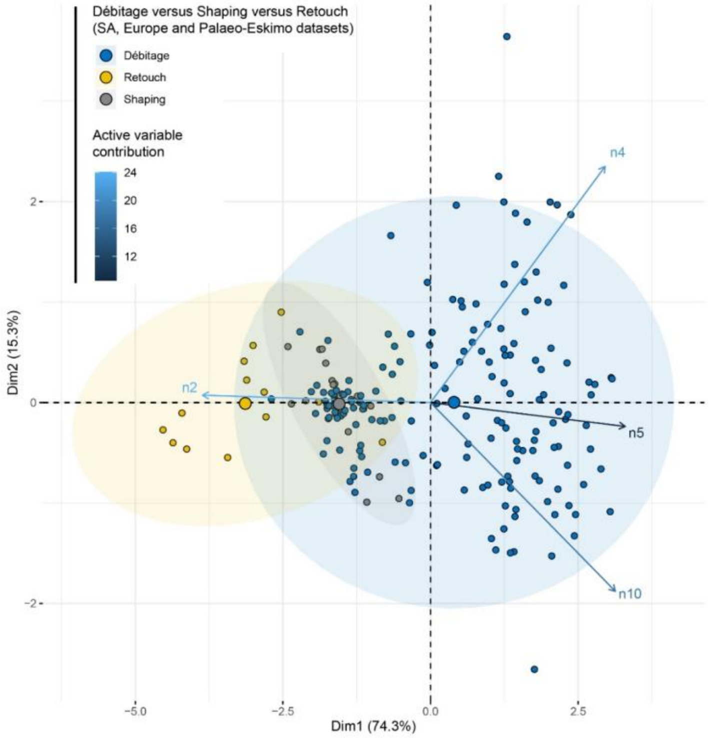

Fig 16: PCA comparing debitage, shaping and retouch from the SA dataset, ~~presented in this paper and the~~ EU dataset, and ~~the~~ ESKI dataset [20,21].

Is the particle size distribution of the SA dataset different ~~to~~ from the RUNOFF dataset?

The factorial space of the SA dataset compared to the RUNOFF model [4] (Fig 17) shows a clear opposition on the first dimension (53% of the variability) between, on one hand, the fine accumulation area composed of smaller remains (2 to 4 mm size class) and, on the other hand, ~~industries~~ knapping methods (SA dataset) and runoff areas as residual and transit (in and off channel) described by particles over 4 mm wide. The SA experiments overlap with most of the runoff accumulations of large particles.

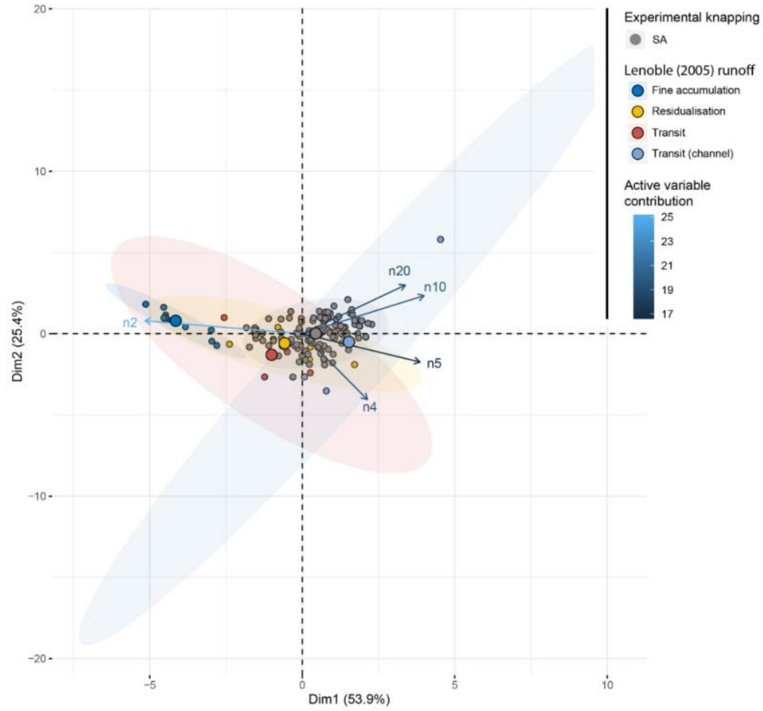

Fig 17: PCA comparing the SA dataset to the RUNOFF dataset [4].

Discussion

Variability within the SA dataset

Patterson [41]: p. 550 highlighted the need ~~of~~ for ‘simple analytical technique~~e~~s’ for ~~analysing~~ examining lithic assemblages that can be used ~~for~~ by any archaeologist (and not only lithic specialists). This still seems ~~still today~~to be a valid point today as lithic assemblages have the advantage of preservation over organic remains, and they ~~suppose~~ provide the most evident continuous cultural record ~~still~~ available from the past [42–44].

The results of our experiment highlight two main points within the SA dataset: a distinction between raw materials and ~~the~~ a difference between debitage produced by freehand and bipolar ~~sets~~knapping techniques.

Concerning the raw materials, we see, on the one hand, that in our experimental sample chalcedony, X-quartz, A-quartz, and jasper group~~s~~ more or less together and produce smaller size particles; on the other hand, we find~~ound~~ rhyolite, quartzite and hornfels producing wider size ranges (Figs 8 and 10).

Other authors like K. Schick [2] have also highlighted differences regarding raw materials. Our results prove that experiments ~~with~~ using specific raw materials ~~of~~ from the ~~areas of study of~~regions associated with archaeological assemblages are needed to understand particle size ~~analysis~~distribution in Stone Age lithic assemblages.

Regarding the distinction between freehand and bipolar ~~sets~~knapping methods, from a particle size analysis point of view, it seems possible to distinguish between these two ~~big variants~~types of reduction. This is in accordance with previous characterizations made to ~~distinguish~~ quantitatively distinguish freehand from bipolar axial knapping [13,45,46]. This should be tested in future archaeological analyses where freehand and bipolar knapping have been documented within the same assemblage, ~~like~~ such as the Howiesons Poort of Sibudu [47,48], ~~or~~ and the miniaturization strategies at B~~l~~oomplaas [49]. It would be interesting to test if ~~only from the multivariate analysis of the particle size~~ this distinction between freehand and bipolar is feasible considering that the distinction has been informed by the multivariate analysis of the particle size. The reason ~~to~~ we mention ~~those~~ previous technological analyses is that the main way of identifying bipolar knapping in those studies was through the cores, as it seems the easier way to recognize ~~bipolar~~ the reduction technique [50]. If ~~this gets~~distinguishing between freehand and bipolar knapping by means of particle size is confirmed, this experiment would have made a significant contribution to the field. ~~would be a major contribution of this experimental set.~~

Regarding the different freehand knapping methods in our sample, in the factorial space, it does not seem possible to differentiate unidirectional, discoidal and *Levallois* flaking systems. However, as we have not knapped each raw material through all knapping methods, it remains difficult to know what

variable (either the knapping method or the raw material) causes these differences. Our results show that the size distribution in ~~S~~southern African archaeological assemblages is knapping method specific and raw material specific.

Due to the variability expressed by the SA dataset, the selection of a single raw material and knapping method appropriate to the site context provides a more accurate model for performing a particle size analysis. Moreover, when combining a specific raw material and a specific knapping method, the distribution areas tend to reduce (with and without 95% confidence intervals) in both~~,~~ ternary plots~~,~~ and principal component analyse~~i~~s.

Differences between SA, EU and ESKI datasets

Southern African lithic industries produce a greater proportion of bigger particles whatever the raw material and the knapping method (Fig 13). In the s~~S~~outhern African dataset, the smallest particles (2 to 4 mm) always constitute 50 to 60% of the reduction sequences. In the EU dataset [20,21] (see Fig 13 therein) the percentage of this fraction was slightly higher, between 60-70% in all their variants.

It seems that our knapping and raw material particle size distribution differs from the ones published in Bertran and colleagues [20,21]. In the case of the EU dataset [20,21], the different experiments overlap remarkably in the multivariate analysis and in the ternary plots (Fig 11), whereas our dataset has a much wider distribution (Fig 13).

The EU, ESKI and SA datasets are clearly separated from each other. The reasons for these different groupings might be due to different aspects. With the evidence at hand, we can only propose some hypotheses that should be tested in future experiments.

Firstly, some technical behaviours produce more small particles. This is particularly the case of microlithic production and retouching activities (ESKI dataset) and to a lesser degree, for bipolar

knapping on anvil (SA dataset) and shaping (EU and ESKI datasets). Similar results have been pointed out in other experimental studies [2,20,21,41].

Secondly, the differences between Bertran and colleagues [20,21] and our sample regarding freehand variants could be ~~a~~ the result of the level of ~~the~~ skill of the knappers, due to ~~the~~ specific knapper gestures and procedures. In other words, it should not exclude the hypothesis that knapper ~~ability~~ competence and experience have an impact on particle size distribution. Nonetheless, these different levels of knapping performance enrich the models, as it is expected that~~n~~ in the past there would also have been variability in knapping skills within cultural traditions and periods. Moreover, despite similar knapping goals, knapper-specific gestures, habits, and procedures (such as platform preparation or removing striking platform overhang) could also affect size distribution. Again, these variables would require~~est~~ additional experiments.

Thirdly, raw material specificities also induce different patterns in terms of particle size distribution. Whereas flint knapping experiments have a narrow distribution, other types of raw material induce specific distributions with various dispersion~~s~~ features. Regardless of the knapping method, flint tends to produce finer particles than A-quartz, X-quartz, jasper, and chalcedony, which in turn produce finer particles than quartzite, rhyolite and hornfels. Finer raw materials (i.e., flint, jasper, and chalcedony), A-quartz and X-quartz produce finer grain sizes in contrast to coarser metamorphic and volcanic raw materials like quartzite, hornfels and rhyolite.

Finally, considering previous knapping datasets [20,21] and our knapping experiments, it seems clear that blank transformation (“retouching”) produces smaller particles than “shaping” and blank production (debitage). The intensity of retouch and site function [34] also influence the particle size distribution of industries as ~~it~~ is already shown in Bertran et al. [20–22]. When a great proportion of the blank is transformed on ~~a~~ site, we ~~could~~ would expect a greater proportion of the small fraction (2 to 4 mm).

SA knapping dataset versus RUNOFF experiments

The comparison of the SA dataset distribution and the RUONFF model [4] (for flint) shows a remarkable amount of overlapping.

While taken as a whole, the SA dataset shows a broad distribution (contra EU dataset) (Fig 13 and 17). At a site scale, it is nevertheless possible to reduce that distribution by considering only a single knapping method on a single raw material. This will reduce the overlapping with the RUNOFF model and allow a more accurate comparison to assess the degree of perturbation in the archaeological record. perturbation degree. The overlapping between the SA and the RUNOFF dataset could also be also due to the different densities of the rockraw materials. Igneous rocks, such as rhyolite are denser that sedimentary rocks and this could have an impact on this general comparison. This overlapping is also pointing out the need for further experimentation that clarifies the potential reasons for this situation. The new experiments should take into account the geomorphological frequent scenarios for site formation deposits in southern Africa assemblages.

Finally, the overlapping between the SA and the RUNOFF datasets is pointing out the need offor further experimentation that clarifies the potential reasons for this situation. The new experiments should take into account the geomorphological common scenarios for site formation deposits in southern Africa assemblages.

Our results are also a call of attention to the fact that particle size analyses although useful, should be complemented with other taphonomical methodologies in order to fully comprehend site formation processes [5].

[Sorry, but this sentence above and the following sentences are introducing a completely different topic related to vertebrate taphonomy, and if you go there, there is a LOT of literature that needs to be cited, and to be honest, it doesn't add to your research, it only introduces problems. My advice is to delete it. If the reviewers requested that you add something like this, then let me know and I will work on it]

Formatted: Not Highlight

Formatted: Not Highlight

Formatted: Highlight

Formatted: Highlight

Formatted: Highlight

Also, wWhen a lithic assemblage is sorted because of a water flow, an extreme caution needs to be taken when considering faunal remains that are more mobile in these contexts [51]. Faunal remains have a higher transport capacity due to their density and their shape, more compact compared to the platy shape of most of the lithic remains (flakes, blades and, bladelets). That means, if one gets a sorted lithic assemblage on a site, a greater impact (sorting) must be expected on the associated faunal assemblage, sometimes, almost all the faunal assemblage has been eroded [51]. (Thomas et al, in progress). Unfortunately, the fragility of bone remains does not allow to predict their size distribution a similar approach, as experimental models would be extremely diverse for bones (e.g., dramatic effect of fire activities or trampling on the bone fragmentation.

While our experiment has produced very concrete results, the size distribution analysis of lithic material is not sufficient on its own to assess the perturbation of an archaeological lithic assemblage and must be coupled with other analytical tools. In this veinregard, along a sedimentary analysis [3], fabric analysis [52–54], refitting [17,55–58]-, spatial analysis [59,60], material proportions [51], faunal analysis in terms of taphonomy [Stratford et al. 202262], and or dating [5] are all tools that can help characterize the impact of natural processes on archaeological assemblages. When all of them are combined [51,59], we can reach a very detailed site formation model andby which to gauge the degree of perturbation of the its associated lithic assemblage. perturbation degree.

The overlapping between the SA and the RUNOFF dataset could be also due to the different densities of the rock materials. Igneous rocks, such as rhyolite are denser that sedimentary rocks and this could have an impact on this general comparison.

Finally, the overlapping between the SA and the RUNOFF datasets is pointing out the need of further experimentation that clarifies the potential reasons for this situation. The new experiments should take into account the geomorphological common scenarios for site formation deposits in southern Africa assemblages.

Concluding remarks

Formatted: Not Highlight
Formatted: Not Highlight
Formatted: Not Highlight
Formatted: Not Highlight
Formatted: Not Highlight
Formatted: Not Highlight
Formatted: Highlight
Formatted: Not Highlight
Formatted: Not Highlight
Formatted: Not Highlight
Formatted: Not Highlight
Formatted: Not Highlight
Formatted: Not Highlight
Formatted: Not Highlight

The creation of a new experimental knapping dataset focusing ion southern Africa debitage and raw materials and its comparison to previous datasets have shown a more complex picture for particle size analysis than previously thought. There are two main aspects that do not match with recent investigations on particle size analysis in Euroorpe: on the one hand the wider distribution of our experimental knappingthe huge overlap between our SA dataset and the RUNOFF dataset, on the other hand the wider distribution of our experimental knapping the huge overlap between our SA dataset and the RUNOFF dataset. Our results highlight the need to perform more experimentation to clarify and further support the preliminary distinctions proposed here and the contradictions regarding previous experimental sets.

Our results are also a call of attention to the fact that particle size analyses although useful, should be complemented with other taphonomical methodologies in order to fully comprehend site formation processes.

In this vein, along a sedimentary analysis [49], fabric analysis [22, 4, 28] refitting [10, 11, 45, 54, 55], spatial analysis [18, 26] material proportions [51] or dating [6] are all tools that can help characterize the impact of natural processes on archaeological assemblages. When all of them are combined [18, 51], we can reach a very detailed site formation model and its associated assemblage perturbation degree.

Conclusion

Our results highlight the need to perform more experimentation to clarify and further support the preliminary distinctions proposed here and the contradictions with previous experimental work.

While all knapping experiments show the same general pattern of size distribution (following a Weibull function, i.e., proportion decreasing as size increases [23]), we have demonstrated that the knapping method, blank transformation activity intensity, and raw material, explain most of the variability. The creation of a new experimental knapping dataset focusing on southern African debitage and raw materials, and its comparison towith previous datasets have shown a more complex picture for particle

Formatted: Not Highlight

Formatted: Not Highlight

Formatted: Not Highlight

Formatted: Not Highlight

size analysis than was previously thought. There are two main aspects that do not match with recent investigations on particle size analysis in Europe: on the one hand the wider distribution of our experimental knapping, on the other hand the huge overlap between our SA dataset and the RUNOFF dataset. ~~Our results highlight the need to perform more experimentation to clarify and further support the preliminary distinctions proposed here and the contradictions regarding previous experimental sets. While all knapping experiments show the same general pattern of size distribution (following a Weibull function, i.e., proportion decreasing as size increases [23], we have demonstrated that the knapping method, blank transformation activity intensity, and raw material explain most of the variability.The wide distribution of the SA dataset leads to a significant overlap with the RUNOFF database.~~ To reduce this overlap, the experimental debitage model compared to the RUNOFF dataset should be knapping method and raw material specific. This means that the performance of a particle size analysis at a site must be done in combination with a technological study of the lithic material. The latter guides the choice of the knapping method and raw material to be included in the comparison model between experimental knapping and runoff experiences on sorting ~~[4]~~. Both analyses are clearly dependent on one another (i.e., particle size distribution and technology). The first conditions the interpretation of the second and the second makes the first more accurate ~~[51,61]~~.

Formatted: Not Highlight

Acknowledgements

~~This research was supported by a xxx grant.....~~

Formatted: Font: Not Bold

We thank Gary Trower and Ben Maclennan for indicating to us raw material outcrops and providing us with some of the raw material we ~~have~~ used in our knapping experiments. Our thanks also go to ~~Dr.~~ Lucinda Backwell, Tammy Hodgskiss, Matt Caruana and Matt Lotter, whose reading and correction of the article ~~was~~ were very valuable ~~to us~~.

References

1. Schiffer MB. Toward the identification of formation processes. American Antiquity. 1983;48: 675–706.
2. Schick KD. Stone Age sites in the making: experiments in the formation and transformation of archaeological occurrences. BAR Publishing; 1986.
3. Texier J. A propos des processus de formation des sites préhistoriques/About prehistoric site formation processes. Paléo, Revue d'Archéologie Préhistorique. 2000;12: 379–386.
4. Lenoble A. Ruissellement et formation des sites préhistoriques: référentiel actualiste et exemples d'application au fossile. British Archaeological Report; 2005.
5. Bertran P, Todisco D, Bordes J-G, Discamps E, Vallin L. Perturbation assessment in archaeological sites as part of the taphonomic study: a review of methods used to document the impact of natural processes on site formation and archaeological interpretations. PALEO Revue d'archéologie préhistorique. 2019; 52–75.
6. Bishop WW. Paleogeomorphology and continental taphonomy. Fossils in the Making: Vertebrate Taphonomy and Paleoecology, Midway Reprint. 1980; 20–37.
7. Petraglia MD, Nash DT. The impact of fluvial processes on experimental sites. Natural formation processes and the archaeological record. BAR; 1987. pp. 108–130.
8. Petraglia MD, Potts R. Water flow and the formation of Early Pleistocene artifact sites in Olduvai Gorge, Tanzania. Journal of Anthropological Archaeology. 1994;13: 228–254.
9. Bertran P, Klaric L, Lenoble A, Masson B, Vallin L. The impact of periglacial processes on Palaeolithic sites: the case of sorted patterned grounds. Quaternary International. 2010;214: 17–29.
10. Lenoble A, Bertran P, Boulogne S, Masson B, Vallin L. Évolution des niveaux archéologiques en contexte périglaciaire. Apport de l'expérience Gavarnie. Les Nouvelles de l'archéologie. 2009; 16–20.
11. Bordes F, Bourgon M. Le complexe moustérien: Moustériens, levalloisien et tayacien. l'Anthropologie. 1951;55: 1–23.

Formatted: Font: 11 pt

Formatted: Line spacing: 1.5 lines

Formatted: Font: (Default) +Headings (Calibri Light)

Formatted: Justified, Line spacing: 1.5 lines

Formatted: Font: (Default) +Headings (Calibri Light), Spanish (Spain)

Formatted: Font: (Default) +Headings (Calibri Light)

12. de la Peña P, Witelson D. Trampling vs. retouch in a lithic assemblage: a case study from a middle stone age site, steenbokfontein 9KR (Limpopo, South Africa). Journal of Field Archaeology. 2018;43: 522–537.

13. Eren MI, Durant A, Neudorf C, Haslam M, Shipton C, Bora J, et al. Experimental examination of animal trampling effects on artifact movement in dry and water saturated substrates: a test case from South India. Journal of Archaeological Science. 2010;37: 3010–3021.

14. Gifford DP, Behrensmeyer AK. Observed formation and burial of a recent human occupation site in Kenya. Quaternary Research. 1977;8: 245–266.

15. Gifford DP. Ethnoarchaeological observations of natural processes affecting cultural materials. Explorations in Ethno-archaeology. 1978; 77–101.

16. Marwick B, Hayes E, Clarkson C, Fullagar R. Movement of lithics by trampling: an experiment in the Madjedbebe sediments, northern Australia. Journal of Archaeological Science. 2017;79: 73–85.

17. Villa P, Courtin J. The interpretation of stratified sites: a view from underground. Journal of Archaeological Science. 1983;10: 267–281.

18. Yellen JE. Archaeological approaches to the present: Models for reconstructing the past (Studies in archeology). 1977.

19. Sitzia L. Chronostratigraphie et distribution spatiale des dépôts éoliens quaternaires du bassin aquitain. PhD Thesis, Université Sciences et Technologies-Bordeaux I. 2014.

20. Bertran P, Claud É, Detrain L, Lenoble A, Masson B, Vallin L. Composition granulométrique des assemblages lithiques. Application à l'étude taphonomique des sites paléolithiques. PALEO Revue d'archéologie préhistorique. 2006; 7–36.

21. Bertran P, Lenoble A, Todisco D, Desrosiers PM, Sørensen M. Particle size distribution of lithic assemblages and taphonomy of Palaeolithic sites. Journal of Archaeological Science. 2012;39: 3148–3166. doi:10.1016/j.jas.2012.04.055

22. Todisco D, Bhiry N, Resrosiers P. Paleoeskimo Site Taphonomy: An Assessment of the Integrity of the Tayara Site, Qikirtaq Island, Nunavik, Canada. Geoarchaeology: An International Journal. 2009;24: 743–791.

23. Stahle DW, Dunn JE. An analysis and application of the size distribution of waste flakes from the manufacture of bifacial stone tools. World Archaeology. 1982;14: 84–97.

Formatted: Font: (Default) +Headings (Calibri Light), Spanish (Spain)

Formatted: Font: (Default) +Headings (Calibri Light)

24. Pargeter J. Early microlithic technologies and behavioural variability in Southern Africa and South Asia. Taylor & Francis; 2017.

25. Wadley L. Those marvellous millennia: the Middle Stone Age of southern Africa. Azania: Archaeological Research in Africa. 2015;50: 155–226.

26. Crabtree DE. An introduction to Flintworking. Occasional Papers of the Idaho State University Museum. 1972.

Formatted: Font: (Default) +Headings (Calibri Light), Spanish (Spain)

27. de la Peña Alonso P. Sobre la identificación macroscópica de las piezas astilladas: propuesta experimental. Trabajos de Prehistoria. 2011;68: 79–98.

Formatted: Font: (Default) +Headings (Calibri Light)

28. Terradas X. Discoid flaking method: conception and technological variability. BAR International Series. 2003;1120: 19–32.

29. Mourre V. Discoïde ou pas discoïde? Réflexions sur la pertinence des critères techniques définissant le débitage discoïde. BAR International Series. 2003;1120: 1–18.

Formatted: Font: (Default) +Headings (Calibri Light), Spanish (Spain)

30. Boëda E. Le concept Levallois: variabilité des méthodes. Archéo éditions; 1994.

31. Cairncross B. Field guide to rocks & minerals of Southern Africa. Penguin Random House South Africa; 2011.

Formatted: Font: (Default) +Headings (Calibri Light)

Formatted: Font: (Default) +Headings (Calibri Light), Spanish (Spain)

32. Mourre V. Les industries en quartz au Paléolithique. Terminologie, méthodologie et technologie. Paléo, Revue d'Archéologie Préhistorique. 1996;8: 205–223.

33. Sunagawa I. Crystals: growth, morphology, & perfection. Cambridge University Press; 2007.

34. Rodríguez-Rellán C. La anisotropía y el clivaje del cuarzo automorfo y sus posibles efectos sobre la talla: Una revisión bibliográfica. Journal of Lithic Studies. 2015;2: 189–207.

Formatted: Font: (Default) +Headings (Calibri Light)

35. Rodríguez-Rellán C. Variability of the rebound hardness as a proxy for detecting the levels of continuity and isotropy in archaeological quartz. Quaternary International. 2016;424: 191–211.

36. de la Peña P, Val A, Stratford D, Colino F, Esteban I, Fitchett J, et al. Revisiting Mwulu's Cave: new insights into the Middle Stone Age in the southern African savanna biome. Archaeological and Anthropological Sciences. 2019;11: 3239–3266.

37. Allaby M. Dictionary of earth sciences. Oxford University Press; 2008.

38. Hammer Ø, Harper DA, Ryan PD, others. PAST: Paleontological statistics software package for education and data analysis. Palaeontologia electronica. 2001;4: 9.

39. Weaver TD, Boyko RH, Steele TE. Cross-platform program for likelihood-based statistical comparisons of mortality profiles on a triangular graph. Journal of Archaeological Science. 2011;38: 2420–2423.

40. Lê S, Josse J, Husson F. FactoMineR: an R package for multivariate analysis. Journal of statistical software. 2008;25: 1–18.

41. Patterson LW. Characteristics of bifacial-reduction flake-size distribution. American Antiquity. 1990;55: 550–558.

42. Shea JJ. Prehistoric stone tools of Eastern Africa: a Guide. Cambridge University Press; 2020.

43. Shott MJ. Size and form in the analysis of flake debris: review and recent approaches. Journal of archaeological method and theory. 1994;1: 69–110.

44. Stout D. Stone toolmaking and the evolution of human culture and cognition. Philosophical Transactions of the Royal Society B: Biological Sciences. 2011;366: 1050–1059.

45. Pargeter J, Eren MI. Quantifying and comparing bipolar versus freehand flake morphologies, production currencies, and reduction energetics during lithic miniaturization. Lithic Technology. 2017;42: 90–108.

46. Pargeter J, de la Peña P. Milky quartz bipolar reduction and lithic miniaturization: experimental results and archaeological implications. Journal of Field Archaeology. 2017;42: 551–565.

47. de la Peña P, Wadley L. Quartz knapping strategies in the Howiesons Poort at Sibudu (KwaZulu-Natal, South Africa). PloS one. 2014;9: e101534.

48. de la Peña P. The interpretation of bipolar knapping in African Stone Age studies. Current Anthropology. 2015;56: 911–923.

49. Pargeter J. Lithic miniaturization in late Pleistocene southern Africa. Journal of Archaeological Science: Reports. 2016;10: 221–236.

50. Diez-Martín F, Sánchez P, Domínguez-Rodrigo M, Mabulla A, Barba R. Were Olduvai hominins making butchering tools or battering tools? Analysis of a recently excavated lithic assemblage from BK (Bed II, Olduvai Gorge, Tanzania). Journal of Anthropological Archaeology. 2009;28: 274–289.

51. Thomas M, Discamps E, Gravina B, Texier J-P. Analyse taphonomique et spatiale de palimpsestes d'occupations moustériennes de l'abri inférieur du Moustier (Dordogne, France). PALEO Revue d'archéologie préhistorique. 2019; 278–299.

52. Bertran P, Lenoble A. Fabriques des niveaux archéologiques : méthode et premier bilan des apports à l'étude taphonomique des sites paléolithiques. PALEO Revue d'archéologie préhistorique. 2002; 13–28. doi:10.4000/paleo.1383

53. Isaac GL. Towards the interpretation of occupation debris: some experiments and observations. Kroeber Anthropological Society Papers. 1967;37: 31–57.

54. McPherron SP. Additional statistical and graphical methods for analyzing site formation processes using artifact orientations. PLOS ONE. 2018;13: e0190195. doi:10.1371/journal.pone.0190195

55. Bordes J-G. Lithic taphonomy of the Châtelperronian/Aurignacian interstratifications in Roc de Combe and Le Piage (Lot, France). The chronology of the Aurignacian and of the transitional technocomplexes Dating, stratigraphies, cultural implications. 2003; 223–244.

56. Bordes J-G. À propos de taphonomie lithique: fondements épistémologiques et outils analytiques. Réflexions à partir du thème de la transition du Paléolithique moyen au Paléolithique supérieur. Université de Bordeaux. 2012.

57. Staurset S, Coulson S. Sub-surface movement of stone artefacts at White Paintings Shelter, Tsodilo Hills, Botswana: Implications for the Middle Stone Age chronology of central southern Africa. Journal of Human Evolution. 2014;75: 153–165.

58. Villa P. Conjoinable pieces and site formation processes. American Antiquity. 1982;47: 276–290.

59. Discamps E, Bachellerie F, Baillet M, Sitzia L. The use of spatial taphonomy for interpreting Pleistocene palimpsests: an interdisciplinary approach to the Châtelperronian and carnivore occupations at Cassenade (Dordogne, France). Paleoanthropology. 2019;2019: 362–388.

60. Lenoble A, Texier J-P. Processus géologiques de formation du site d'Isturitz (Sud-Ouest de la France). Implications archéologiques. PALEO Revue d'archéologie préhistorique. 2016; 235–252.

61. Thomas M, Gravina B. Analyse techno-économique d'un assemblage Discoïde du Moustérien récent de l'abri inférieur du Moustier (Dordogne, France). PALEO Revue d'archéologie préhistorique. 2019; 300–317. doi:10.4000/paleo.4933

Formatted: Font: (Default) +Headings (Calibri Light), Spanish (Spain)

Formatted: Font: (Default) +Headings (Calibri Light)

Formatted: Font: (Default) +Headings (Calibri Light), Spanish (Spain)

Formatted: Spanish (Spain)

62. Stratford, D., Clark, J.L., Wojcieszak, M., Wadley, L., d'Errico, F., de la Peña, P. et al. Geoarchaeology and zooarchaeology of Border Cave, South Africa: Initial multiproxy considerations of stratigraphy and site formation processes from the Backwell et al. excavations. Quaternary Science Reviews. 2022; p107618.

Formatted: Font: (Default) +Headings (Calibri Light), 11 pt, Font color: Auto, Spanish (Spain), Pattern: Clear

Formatted: Normal, Justified

Formatted: Spanish (Spain)

Formatted: Font: (Default) +Headings (Calibri Light), 11 pt, Font color: Auto, Spanish (Spain), Pattern: Clear

Formatted: Font: (Default) +Headings (Calibri Light), 11 pt, Font color: Auto, Pattern: Clear

Formatted: Font: (Default) +Headings (Calibri Light)

Dear Editor,

Thank you for letting us revise the manuscript. We are also grateful for the constructive comments of the two reviewers. We have addressed all the comments and problems highlighted in the review.

Yours sincerely,

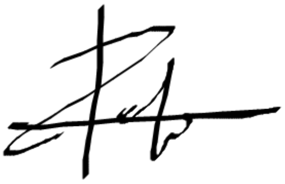

Dr. Paloma de la Peña

In Johannesburg, 11th August 2022

Response to reviewers

Reviewer #1

Have the authors made all data underlying the findings in their manuscript fully available?

We incorporated our database into the supplementary material (SM1) and added some statistical information on the implementation of PCA (SM2) and an R script that allows us to reproduce all PCA analyses and obtain more statistics (SM3).

Is the manuscript presented in an intelligible fashion and written in standard English?

The manuscript was revised in the first and second submission by Dr Tammy Hodgskiss and in the second revision by Dr Lucinda Backwell, both of them are native English-speaking persons very familiar with archaeology vocabulary as they regularly publishes on Middle and Later Stone Age Archaeology.

My biggest criticism concerns the methodical setup of the experimental knapping. There is little explanation or context for what the initial set-up was: Why were 117 cores knapped (how to get to that number)? How was the distribution across raw materials and reduction methods arrived at? Was there any bigger experimental design behind it? Looking at the distribution of cores in Table 1, it honestly looks a bit unsystematic. The reasoning that not all knapping methods were not applied to all raw materials due to the Southern African archaeological context (lines 210-212) is not convincing, as there is evidence for all of the knapping methods on all raw

materials of this study (admittedly much rarer for some raw materials, e.g. Levallois on quartz is rare). This then also compromises some statistical analyses of the study as the authors themselves acknowledge (lines 213-214). Ideally, the study would incorporate some more reduction of different raw materials/knapping methods or be at the very list much clearer about the description of the study design in the Materials and Methods section!

Thank you for this comment. The reviewer is right that the experiment seems a bit unsystematic. The number of experiments is odd because if the core fragmented during the knapping process, we did not include those experiments. Moreover, it has also been influenced by the covid pandemic. The experiment was done in late 2020 and early 2021 when it was not possible to access raw material outcrops due to driving and transport restrictions, so we operate with the material available at the laboratory of Archaeology at the Evolutionary Studies Institute.

Finally, the reason for some raw materials and knapping varieties not represented is that there are some knapping methods that are not reported in the archaeological record (Middle and Later Stone Age). For instance, to the best of our knowledge there is no evidence of *Levallois* recurrent centripetal reduction on automorphic and xenomorphic quartz or, on the other hand, there is no documentation on bipolar knapping on hornfels. It would have no archaeological sense to include those varieties in the experimental collection.

This has been clarified in the presentation of the methodology experimentation.

While the results are well-done and well-presented, the discussion is somewhat lacking substance and not yet sufficient for publication. Most importantly, the authors should at least provide one test application (i.e. case study) showing the value of their experimental study and the particle size distribution in one MSA or LSA assemblage from southern Africa. This would clearly show the analytical value of the current study for archaeology, which is so far only assumed but not shown. Particularly, this could show with empirical data why their database and not the EU dataset should be used for such an analysis. Additional discussion on the potential insights that can be gained from the experimental database for MSA/LSA studies from a general potential would also much add to the relevance of the current study.

We have reinforced and re-done the discussion adding new ideas and an in depth criticism of our results (see the changes in the final section)

We think it is a very good idea to compare our experimental results with archaeological collections and, indeed, we would like to do another research paper only for that comparison. We wanted first to publish the experimental material and to compare it to other experimental studies such as Schick (1986, 1987) or Bertran et al. (2006, 2012), which are valuable contributions for African and European assemblages respectively.

Finally, some parts of the manuscript such as the Introduction (lines 48-54) and several parts later om need some further polishing of the English language, potentially by the additional help of a native speaker.

The manuscript was revised in the first and second submission by Dr Tammy Hodgskiss and in the second revision also by Dr Lucinda Backwell, both of them are native English speaking persons very familiar with archaeology vocabulary as they regularly publishes on Middle and Later Stone Age Archaeology.

Reviewer #2

The paper would benefit of a more accurate description of the reason why a South African dataset has been compared with European datasets.

The authors assess that particle size distribution variability is strongly affected by raw materials and knapping methods, however it is not clearly explained if to demonstrate this diversity was the main reason behind their choice to perform comparative analyses between different contexts and different raw materials. I think that much benefit will be provided by clarifying since the beginning of the paper what is the main purpose of this research.
It is true that raw material variability strongly affects the results of these type of analysis, thus my question is if it is worth it to compare different débitage methods performed on different raw materials from different geographical contexts. It would be useful if the authors will dedicate a section in the paper to explain this main decision.

The only available datasets made thanks to the Bertran et al methodology describe mainly European archaeological context. That was the main reason of this comparison and doing these experiments and the datasets on particle size. Moreover, particle size analysis in southern Africa archaeological collections have not been applied before. To have a valid framework it needs to be addressed from the most frequent raw materials and the knapping methods in this chronology.

The main purpose of the paper is to offer a framework to understand the particle size distribution on Southern African sites of the Late Pleistocene. Following this reviewer advice we have reinforced this in the manuscript (see the abstract, the introduction and the “Questions and objectives” sections.

Furthermore, the statistical analyses are performed in the correct way. However, it is quite difficult to navigate through the PCA results without knowing any information regarding the loadings and the scores that each principal component provides. In this case, the graphs alone it is not enough to understand the degree of variance of the analyzed variables.
I would appreciate an additional table with variables used for comparative analyses,

including the variables used from the datasets from previous experimental analyses. At this point, there are no information regarding the normalization of the data between the different datasets

We have included the statistics information associated to the PCA in SM 2 and 3.

We have also included in SM1 the datasets of previous experimental analyses together with our own experimentation, everything studies and formatted in the same way (sieving with the same screen mesh for at least 4 size class).

...

We have also modified figures 1, 6 and 14 as there were some typos.

Finally, we would like to change our title to: *Particle size distribution: an experimental study using southern African reduction methods and raw materials*
