# Supplementary material for: Particle size distribution: An experimental study using southern African reduction methods and raw materials
Source: PLoS One. 2022 Dec 30;17(12):e0278867. doi: 10.1371/journal.pone.0278867 (PMC9803312; doi:10.1371/journal.pone.0278867)
Supplement: Attachment — Submitted filename: Rebuttal letter 24_8_22.docx [file pone.0278867.s006.docx]

Dear Editor,

Thank you for letting us revise the manuscript. We are also grateful for the constructive comments of the two reviewers. We have addressed all the comments and problems highlighted in the review.

Yours sincerely,

Dr. Paloma de la Peña

In Johannesburg, 11^th^ August 2022

**Response to reviewers**

**Reviewer #1**

*Have the authors made all data underlying the findings in their manuscript fully available*?

We incorporated our database into the supplementary material (SM1) and added some statistical information on the implementation of PCA (SM2) and an R script that allows us to reproduce all PCA analyses and obtain more statistics (SM3).

*Is the manuscript presented in an intelligible fashion and written in standard English?*

The manuscript was revised in the first and second submission by Dr Tammy Hodgskiss and in the second revision by Dr Lucinda Backwell, both of them are native English-speaking persons very familiar with archaeology vocabulary as they regularly publishes on Middle and Later Stone Age Archaeology.

*My biggest criticism concerns the methodical setup of the experimental knapping. There is little explanation or context for what the initial set-up was: Why were 117 cores knapped (how to get to that number)? How was the distribution across raw materials and reduction methods arrived at? Was there any bigger experimental design behind it? Looking at the distribution of cores in Table 1, it honestly looks a bit unsystematic. The reasoning that not all knapping methods were not applied to all raw materials due to the Southern African archaeological context (lines 210-212) is not convincing, as there is evidence for all of the knapping methods on all raw materials of this study (admittedly much rarer for some raw materials, e.g. Levallois on quartz is rare). This then also compromises some statistical analyses of the study as the authors themselves acknowledge (lines 213-214). Ideally, the study would incorporate some more reduction of different raw materials/knapping methods or be at the very list much clearer about the description of the study design in the Materials and Methods section!*

Thank you for this comment. The reviewer is right that the experiment seems a bit unsystematic. The number of experiments is odd because if the core fragmented during the knapping process, we did not include those experiments. Moreover, it has also been influenced by the covid pandemic. The experiment was done in late 2020 and early 2021 when it was not possible to access raw material outcrops due to driving and transport restrictions, so we operate with the material available at the laboratory of Archaeology at the Evolutionary Studies Institute.

Finally, the reason for some raw materials and knapping varieties not represented is that there are some knapping methods that are not reported in the archaeological record (Middle and Later Stone Age). For instance, to the best of our knowledge there is no evidence of *Levallois* recurrent centripetal reduction on automorphic and xenomorphic quartz or, on the other hand, there is no documentation on bipolar knapping on hornfels. It would have no archaeological sense to include those varieties in the experimental collection.

This has been clarified in the presentation of the methodology experimentation.

*While the results are well-done and well-presented, the discussion is somewhat lacking substance and not yet sufficient for publication. Most importantly, the authors should at least provide one test application (i.e. case study) showing the value of their experimental study and the particle size distribution in one MSA or LSA assemblage from southern Africa. This would clearly show the analytical value of the current study for archaeology, which is so far only assumed but not shown. Particularly, this could show with empirical data why their database and not the EU dataset should be used for such an analysis. Additional discussion on the potential insights that can be gained from the experimental database for MSA/LSA studies from a general potential would also much add to the relevance of the current study.*

We have reinforced and re-done the discussion adding new ideas and an in depth criticism of our results (see the changes in the final section)

We think it is a very good idea to compare our experimental results with archaeological collections and, indeed, we would like to do another research paper only for that comparison. We wanted first to publish the experimental material and to compare it to other experimental studies such as Schick (1986, 1987) or Bertran et al. (2006, 2012), which are valuable contributions for African and European assemblages respectively.

*Finally, some parts of the manuscript such as the Introduction (lines 48-54) and several parts later om need some further polishing of the English language, potentially by the additional help of a native speaker.*

The manuscript was revised in the first and second submission by Dr Tammy Hodgskiss and in the second revision also by Dr Lucinda Backwell, both of them are native English speaking persons very familiar with archaeology vocabulary as they regularly publishes on Middle and Later Stone Age Archaeology.

**Reviewer #2**

The paper would benefit of a more accurate description of the reason why a South African dataset has been compared with European datasets.

The authors assess that particle size distribution variability is strongly affected by raw materials and knapping methods, however it is not clearly explained if to demonstrate this diversity was the main reason behind their choice to perform comparative analyses between different contexts and different raw materials. I think that much benefit will be provided by clarifying since the beginning of the paper what is the main purpose of this research.
It is true that raw material variability strongly affects the results of these type of analysis, thus my question is if it is worth it to compare different débitage methods performed on different raw materials from different geographical contexts. It would be useful if the authors will dedicate a section in the paper to explain this main decision.

The only available datasets made thanks to the Bertran et al methodology describe mainly European archaeological context. That was the main reason of this comparison and doing these experiments and the datasets on particle size. Moreover, particle size analysis in southern Africa archaeological collections have not been applied before. To have a valid framework it needs to be addressed from the most frequent raw materials and the knapping methods in this chronology.

The main purpose of the paper is to offer a framework to understand the particle size distribution on Southern African sites of the Late Pleistocene. Following this reviewer advice we have reinforced this in the manuscript (see the abstract, the introduction and the “Questions and objectives” sections.

Furthermore, the statistical analyses are performed in the correct way. However, it is quite difficult to navigate through the PCA results without knowing any information regarding the loadings and the scores that each principal component provides. In this case, the graphs alone it is not enough to understand the degree of variance of the analyzed variables.
I would appreciate an additional table with variables used for comparative analyses, including the variables used from the datasets from previous experimental analyses. At this point, there are no information regarding the normalization of the data between the different datasets

We have included the statistics information associated to the PCA in SM 2 and 3.

We have also included in SM1 the datasets of previous experimental analyses together with our own experimentation, everything studies and formatted in the same way (sieving with the same screen mesh for at least 4 size class).

...

We have also modified figures 1, 6 and 14 as there were some typos.

Finally, we would like to change our title to: *Particle size distribution: an experimental study using southern African reduction methods and raw materials*
